# Mapping Large-Scale Networks Associated with Action, Behavioral Inhibition and Impulsivity

**DOI:** 10.1523/ENEURO.0406-20.2021

**Published:** 2021-02-23

**Authors:** L. Fakhraei, M. Francoeur, P. Balasubramani, T. Tang, S. Hulyalkar, N. Buscher, C. Claros, A. Terry, A. Gupta, H. Xiong, Z. Xu, J. Mishra, D. S. Ramanathan

**Affiliations:** 1 Mental Health Service, VA San Diego Healthcare System, La Jolla, CA 92161; 2 Department of Psychiatry, University of California San Diego, La Jolla, CA 92093

**Keywords:** behavioral inhibition, brain mapping, impulsivity, local field potentials, orbitofrontal cortex, oscillations

## Abstract

A key aspect of behavioral inhibition is the ability to wait before acting. Failures in this form of inhibition result in impulsivity and are commonly observed in various neuropsychiatric disorders. Prior evidence has implicated medial frontal cortex, motor cortex, orbitofrontal cortex (OFC), and ventral striatum in various aspects of inhibition. Here, using distributed recordings of brain activity [with local-field potentials (LFPs)] in rodents, we identified oscillatory patterns of activity linked with action and inhibition. Low-frequency (δ) activity within motor and premotor circuits was observed in two distinct networks, the first involved in cued, sensory-based responses and the second more generally in both cued and delayed actions. By contrast, θ activity within prefrontal and premotor regions (medial frontal cortex, OFC, ventral striatum, and premotor cortex) was linked with inhibition. Connectivity at θ frequencies was observed within this network of brain regions. Interestingly, greater connectivity between primary motor cortex (M1) and other motor regions was linked with greater impulsivity, whereas greater connectivity between M1 and inhibitory brain regions (OFC, ventral striatum) was linked with improved inhibition and diminished impulsivity. We observed similar patterns of activity on a parallel task in humans: low-frequency activity in sensorimotor cortex linked with action, θ activity in OFC/ventral prefrontal cortex (PFC) linked with inhibition. Thus, we show that δ and θ oscillations form distinct large-scale networks associated with action and inhibition, respectively.

## Significance Statement

Using multisite local-field potential (LFP) recordings, we have identified large-scale brain networks involved in action and inhibition in segregated brain regions. Successful inhibition is predicted most strongly by the strength of functional coupling particularly between motor cortex, orbitofrontal cortex (OFC), and ventral striatum.

## Introduction

Impulsivity has been recognized as a key dimension of behavior that co-occurs with various neuropsychiatric disorders (such as bipolar disorder, ADHD, and brain injury; Moeller et al., 2001). Greater impulsivity can lead to worse clinical outcomes in such disorders, including an increased propensity for addiction, suicide, and poor decision-making more generally (Swann et al., 2005; Dalley and Robbins, 2017). While pharmacologic agents can ameliorate some forms of impulsivity (Moeller et al., 2001), a better understanding of the underlying brain systems associated with impulsivity is a key first step toward developing treatments targeted at this particular dimension of psychopathology (Dalley and Robbins, 2017). Progress has been made through the careful development of behavioral tasks that measure specific aspects of behavioral inhibition and thus impulsivity.

In this study, we focused on identifying physiological correlates of action-postponement (waiting). Prior work in this area, using various tasks, has suggested that motor and prefrontal cortex (PFC) and striatum are involved in decisions to either “go” or “wait.” Single-unit electrophysiological studies have shown that motor cortex [primary motor cortex (M1)] and anterior parts of secondary motor cortex (M2) may be involved in decisions to act (in particular, to act more impulsively and wait less; Murakami et al., 2014, 2017; Hardung et al., 2017). Neurons in M2 (Murakami et al., 2014), prefrontal and striatal cortex (Narayanan, 2016; Kim et al., 2017) show ramp-to-threshold activity directly linked with timing or waiting decisions. This suggests that these areas may be directly involved in the decision to act. In addition to these motor regions, activation of the nucleus accumbens shell (NAcS) via DBS (Sesia et al., 2008, 2010; or modulation of dopamine receptors in this area, Besson et al., 2010) increases impulsive responding, linking action with dopaminergic circuits involved in appetitive/approach behavior (Murphy et al., 2008; Basar et al., 2010).

Regions previously shown to be involved in waiting, by contrast, include medial PFC (mPFC), orbitofrontal cortex (OFC), and ventral striatum (in particular, nucleus accumbens core). Single-unit activity in mPFC (along with θ oscillations in this brain region) have been linked with the ability to accurately perform a temporal-based timing task (requiring animals to wait a certain period of time between responses). mPFC may accomplish this via regulation of motor/premotor cortex (Narayanan and Laubach, 2006; Narayanan et al., 2013; Emmons et al., 2017; Hardung et al., 2017; Kim and Narayanan, 2019). Inactivation of the dorsomedial PFC results in more impulsive actions and less ability to wait (Narayanan et al., 2006; Hardung et al., 2017). Neurons in the OFC also seem to play a key role in waiting (Lak et al., 2014; Xiao et al., 2016; Hardung et al., 2017). In addition to prefrontal brain systems involved in this form of behavioral inhibition, pharmacologic and lesion studies have implicated ventral striatum and nucleus accumbens with improved ability to wait (Murphy et al., 2008; Basar et al., 2010; Dalley et al., 2011), and lesions of afferents to these brain regions, including ventral PFC (Chudasama et al., 2003), anterior insula (Belin et al., 2016), and ventral hippocampus/amygdala (Chudasama et al., 2003; Abela et al., 2013) can lead to problems waiting and impulsive early responses.

In total, these studies suggest that distributed brain networks are involved in pushing animals to act optimally in accordance with both internal goals (appetitive/reward-based systems such a ventral striatum, accumbens), external rules (dorsal PFC/premotor cortex) and value tracking (OFC) to maximize the attainment of rewards. Specific decisions related to timing of actions related to a complex interplay between these regions and lower-level motor circuits. However, simultaneous measurements of physiological activity in all of these brain regions have not been done. It is technically challenging to measure wide-spread brain activity in rodents during complex decision-making tasks at a cellular resolution. Local-field potentials (LFPs) are thus a useful tool to probe large-scale, mesoscopic circuit dynamics that can bridge the micro/macro levels of analysis (Hultman et al., 2016, 2018; Pesaran et al., 2018). Similar approaches to using multisite LFP have proven useful in characterizing brain activity/connectivity involved in both slower-changes in behavioral states in mice (Hultman et al., 2016, 2018) and humans (Kirkby et al., 2018), as well as more rapid dynamics associated with cognition and behavior in primates (Markowitz et al., 2011; So et al., 2014; Fiebelkorn et al., 2018; Pesaran et al., 2018). Here, we used simultaneous multisite recordings of LFPs in key brain regions noted above as animals performed a waiting task, and probed large-scale physiological correlates related to action and inhibition. We compared results from animals with data gathered from electroencephalography (EEG) recordings in humans on a similar task.

## Materials and Methods

### Animal methods

Eleven male Long–Evans rats obtained from Charles River Laboratories were used for these experiments. Rats were approximately one month old weighing 150 g when received, and started training approximately two weeks after arrival. Rats were housed in pairs during training before electrode implantation, and individually housed postimplantation in a standard rat cage (10 × 10.75 × 19.5 inches) with free access to food and on a standard light cycle (lights on at 6 A.M./off at 6 P.M.). During behavioral training, animals underwent water scheduling (free access to water for 2 h/d) to maintain motivation for water reward in the task. Rats were weighed weekly to ensure that water scheduling did not lead to reduced food intake. Water was given *ad libitum* on days with no behavioral training. Repeated measurements were taken in animals across days, for a total sample size of 60 sessions.

#### Operant chamber and training

The custom-designed operant chamber used for this study consists of five noseports (NPs), each of which has an LED, IR sensor and metal cannula that delivers water rewards. The chamber also contains two auditory tone generators, a house-light, a screen to display visual stimuli, and five peristaltic stepper motors/water pumps that deliver the water rewards into NPs. The chamber was controlled using Simulink (MathWorks) installed directly onto a Raspberry Pi system. The operant chamber is synchronized with electrophysiological signals using lab-streaming-layer, a protocol designed to integrate multiple behavioral and physiological streams into a common timing (Ojeda et al., 2014, 2020). The design, operation and software control of this chamber has been described in more detail previously (Buscher et al., 2020; Ojeda et al., 2020). Before training on the behavioral task, animals first went through a pretraining period (∼5–10 sessions), in which they learned that a NP with an LED on signaled an available response port; that responding in such a port would trigger a water reward (after a delay of ∼400 ms), and finally that there was a sequential nature to the task (animals start a “trial” by first entering the middle NP 3, after which they could use either of the neighboring ports (2/4) to respond and collect an immediate reward). This “standard” pretraining paradigm is used for all types of behavioral paradigms in our lab, allowing us to develop self-triggered tasks (animals “trigger” the trial to start by entering the middle NP). Animals advanced to the next stage of training when they are able to consistently perform at least 100 trials in 60 min.

#### Go–wait (action delay) task

The rodent version of the self-paced go/wait task was implemented in a custom-designed Raspberry-PI controlled operant chamber (Buscher et al., 2020). Animals begin a trial by entering the middle NP, ensuring animals are in an identical position on every trial when the visual stimulus appears. Visual stimuli displayed for animals as shown in Figure 2*A*. After a short, fixed delay of 30 ms, a visual stimulus appears on the screen denoting that trial to be either a go trial (animal required to respond before 2 s to attain a reward) or wait trial (animal required to respond after 2 s to attain a reward). The stimulus remains on the screen until the animal responds, thus minimally taxing attentional resources. If animals respond correctly, a water reward is delivered after a delay of 400 ms. If animals respond incorrectly, the house-light flashes for a 5-s “time-out” period and no reward are given. Rewards consist of 20 μl of water delivered over a 2-s period using a stepper-motor (which, when activated, is associated with a loud sound providing an instantaneous cue regarding reward delivery). Training on this task proceeded in two stages. In the first stage, animals were trained with a distribution of 75% go and 25% wait trials in order for them to learn to “respond” on certain trial types to attain reward. Once animals achieved >75% accuracy on go trials (typically approximately two weeks), they proceeded to the next stage: a distribution of 25% go/75% wait trials. The ratio was changed to encourage animals to learn adequate waiting, and the bulk of subsequent training was performed at this ratio (we found animals would not learn to wait unless the majority of trials were structured as waiting trials). Animals were trained on this distribution of trials for an additional 12 weeks when behavior typically stabilized (animals generally showed >80% accuracy on go trials by this point with more variable performance on the more difficult wait trials), after which they were implanted with LFP-electrodes as described below. After implantation we waited two weeks to allow animal to recover from surgery before water-scheduling and then performed re-training on the task for an additional one to two weeks before recording. Recordings were typically conducted at least two times per week and were performed with the 25% go/75% wait trial distribution. Our analyses are based on data from 60 recording sessions from 11 rats. All procedures were approved before the start of the study by the Animal Welfare and Ethics committee of the VA. Sessions used for physiological analysis had an average of 272 ± 20.4 (SEM) trials (Extended Data Fig. 1-1*A* for distribution of trial numbers/session).

**Figure 1.**
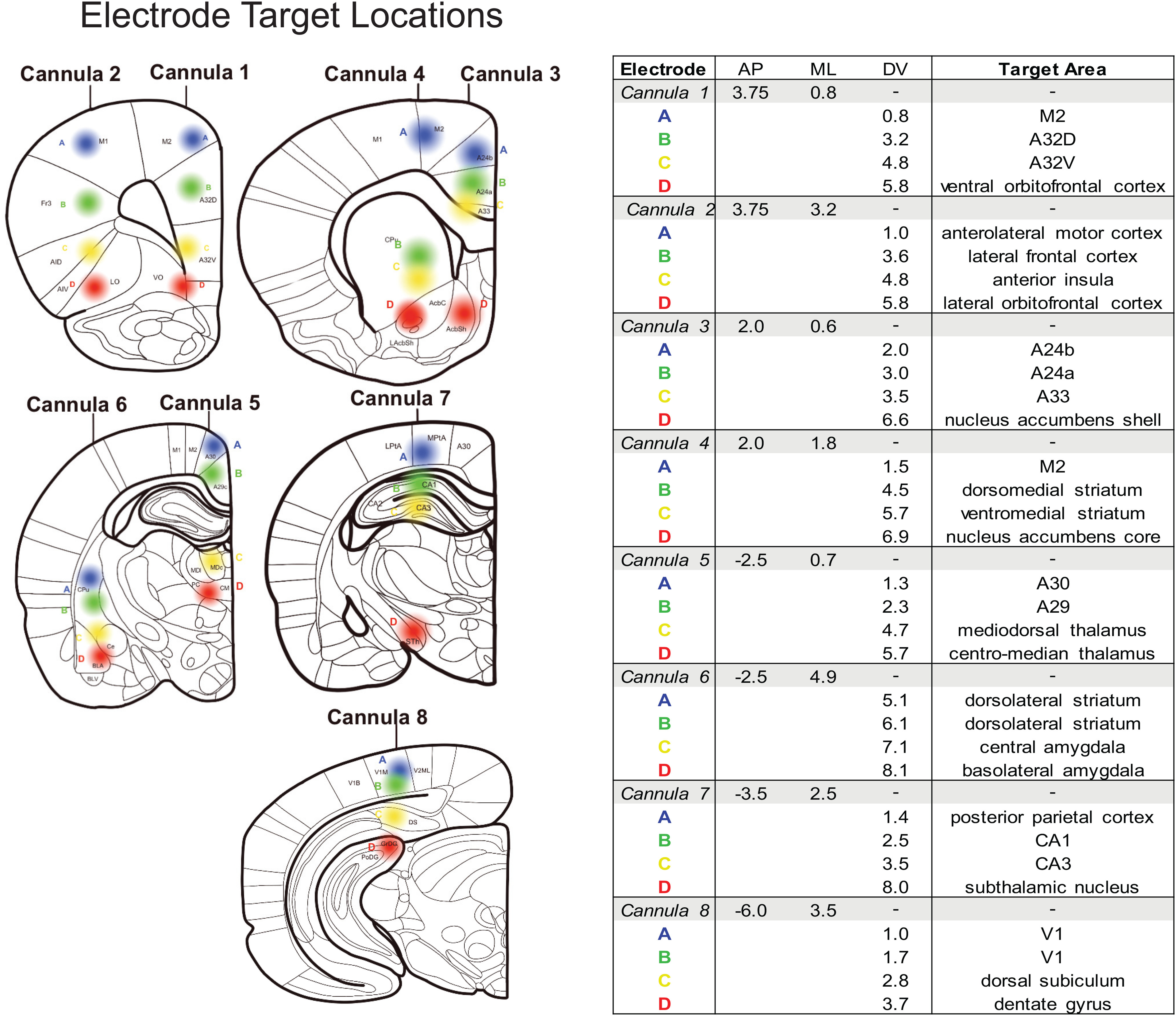
Target electrode locations. Eight cannula each housing four microwires were implanted into the brain at different A/P and M/L locations. Each wire was measured and precut to reach a unique D/V location. This configuration provides 32 target locations within one hemisphere of the brain to record LFPs. All coordinates are calculated and shown relative to bregma (Paxinos and Watson, 2006). Each cannula (1–8) and each electrode wire (A–D) are shown on coronal rat brain sections modified from Paxinos and Watson (2006). Target sites are color coded by depth for reference (A = blue; B = green; C = yellow; D = red). The table contains AP, ML, DV and target location names for all 32 electrodes.

10.1523/ENEURO.0406-20.2021.f1-1 Extended Data Figure 1-1 A. Data preprocessing pipeline, and number of trials for each session included in our analysis. B. Examples of histology related to the multi-site probes. Download Figure 1-1, EPS file.

#### Surgery

Surgery was performed with the “sterile tip” method and all instruments were autoclaved before start. Surgeries were conducted under isoflurane anesthesia (SomnoSuite, Kent Scientific), with a body-temperature controlled heating mat (VWR). Animals received a single dose of Atropine (0.05 mg/kg) to diminish respiratory secretions during surgery and a single dose of dexamethasone (0.5 mg/kg) to decrease inflammation before surgery. A local anesthetic, lidocaine (max 0.2 ml), was injected under the skin at the incision site while the animal was anesthetized but before surgery initiation. Implantation of electrodes was performed under stereotactic control. Using a microdrill (Stoelting), holes were drilled at eight predetermined stereotactic locations for electrodes, ground screw implanted over cerebellum, and a minimum of three anchor screws attached for headstage stability. Electrodes were implanted as “bundles” of four 50-μm tungsten wires (California Fine Wire) that were precut to the appropriate length and secured within a 30-gauge, 8-mm-long metal cannula (Mcmaster-Carr) before surgery. Thus, the depth (D/V position) of each electrode was fixed relative to the four other electrodes in the bundle. The electrode positions were secured with superglue (Loctite) and thus able to be positioned with minimal flexing during surgery. Electrodes were measured such that the cannula did not enter the brain.

Eight different bundles of electrodes were implanted were positioned across various A/P sites (Fig. 1). All coordinates are described relative to bregma (Paxinos and Watson, 2006), and brain targets are likewise named as currently described in the latest edition of Paxinos and Watson. In this nomenclature, to help standardize rodent and non-rodent brain regions, subdivisions of anterior cingulate cortex are used to describe mid-line brain regions rather than rodent-specific names (Laubach et al., 2018; Vogt and Paxinos, 2014). Thus, prelimbic cortex is now described as A32D, infralimbic cortex is A32V, and mid-line regions that have been described previously as M2 or frontal orienting fields (FOFs; Barthas and Kwan, 2017), we describe as A24a, A24b, and A33. Electrode sites were chosen to balance targeting as many potential brain regions of interest (ROIs) across the D/V axis while minimizing the total number of cannulas implanted. Electrode bundles were initially secured with superglue (Loctite) in the craniotomy and on skull, followed by metabond (Parkell). The entire headstage apparatus was held to the skull with dental cement (Stoelting). Once all eight cannulas were placed and cemented to the skull, wires were threaded through the holes of a 36-channel EIB board (Neuralynx) and bolted down using gold pins (Neuralynx). Placing the EIB board above the electrodes, dental cement was used to create a smooth headpiece encapsulating all the wires. This headpiece allowed attachment of the rat to a headstage (Intan) and a commutator (Plexon) inside the behavioral box. At the conclusion of surgery, rats received a single dose (1 mg/kg) of buprenorphine SR for pain management. Rats recovered from surgery on a heating pad to control body temperature and received SMZ-TMP in their drinking water (60 mg/kg/d for 8 d) to prevent infections.

#### Electrophysiology

Electrophysiology data were recorded using a 32-channel RHD headstage (Intantech Part C3324) coupled to a RHD USB interface board (Intantech, Part C3100) with an SPI interface cable. We used plug-in GUI (Open Ephys) software to acquire data. Data were recorded at 1 Khz, with a bandpass filter set at 0.3–999 Hz during acquisition. Physiology data were integrated with operant chamber behavioral data using a lab-streaming-layer protocol (Ojeda et al., 2014), implemented with a customized plug-in written for plug-in GUI (https://github.com/aojeda/plugin-GUI), as described previously (Buscher et al., 2020; Ojeda et al., 2020).

#### LFP analysis

As for preprocessing steps (Extended Data Fig. 1-1*A*), to measure neural activity in brain regions linked to tasks, we conducted standard preprocessing and time frequency (TF) analyses using custom MATLAB scripts and functions from EEGLAB (Ramanathan et al., 2018). (1) Data epoching: we first extracted time points for events of interest during the task (trial start and response). Time-series data were extracted for each electrode, from 2 s before to 5 s after each behavioral marker for each trial and organized into a 3D matrix (electrodes, times, trials). (2) Artifact removal: noisy trials were removed. Trials with >4× the SD in activity (measured across the time dimension) were treated as artifact and discarded. (3) Median reference: activity was then median referenced. At each time point, the “median” activity was calculated across all electrodes and subtracted from each electrode. (4) TF decomposition: a trial by trial TF decomposition (TF decomposition) was calculated using a complex wavelet function implemented within EEGLAB (newtimef function, using Morlet wavelets, with cycles parameter set to: [2, 0.7], frequency window of between 2 and 70 Hz and otherwise default settings used; Delorme and Makeig, 2004). We calculated the analytic amplitude of the signal (using the abs function). (5) Baseline normalization: to measure evoked activity (i.e., change from baseline) we subtracted, for each electrode at each frequency, the mean activity within a baseline window between 1000 and 750 ms before the start of the trial. (6) Trial averaging: we next calculated the average activity across trials for specific trial types (go correct, wait correct or wait incorrect) at each time point and frequency for each electrode, thus creating a 3D matrix (time, frequency, and electrode) for each behavioral session. (8) Comparison across animals: before averaging across sessions/animals, we “z-scored” the data recorded from each behavioral session. This was accomplished by subtracting the mean and dividing by the SD of activity in each electrode (at each frequency) over time. Z-scoring was helpful for normalizing activity measured from different animals before statistical analysis. These preprocessing steps resulted, for each session used in our data analysis, in a 3D TF-electrode (TFE) matrix of dimensions 200 × 139 × 32, which was used for further statistical analyses as described below.

We performed two main types of statistical analyses on the whole-brain TFE data. (1) Trial-type mean: we analyzed the mean and evaluated statistical significance of this mean for each trial type (go correct, wait correct, wait incorrect) for each electrode. Mean was calculated at each time and frequency point for each electrode across the 60 behavioral sessions. Statistical significance was estimated with a one-sample, two-sided *t* test (*t* test function in MATLAB), compared with the null hypothesis of Z = 0. Because we had already performed a “baseline” subtraction (as described above), this analysis was essentially capturing whether there was a significant increase or decrease in activity compared with baseline. False discovery rate (FDR)-correction was applied to the entire TF0-electrode matrix (32 electrodes, 200 time points and 137 frequencies) to evaluate statistical significance at this level. (FDR-corrected *p* value threshold set to 0.05). To visualize significant TF activations or de-activations, non-significant values were set to 0. 2). Trial-type contrast: for many analyses in this article, we were interested in the “contrast” of activity between trial-types (i.e., [go–wait] or [wait–go]). Contrasts were performed by subtracting the mean TFE data matrix for different trial types estimated from each session. We then calculated the average increase between trial types across sessions for either [go–wait] or [wait–go]. Statistical significance was performed with a one-sample, two-sided *t* test applied to this contrast, using a null-hypothesis of 0 (i.e., no difference between trial types). FDR-correction was performed across all times-frequencies-electrodes for whole-brain correction.

For many of these contrasts, we were interested in understanding where there were significant differences, but only in electrodes that show a significant activation for one trial-type in question. For this reason, when evaluating the [go–wait] contrast, we thresholded the activations based on those time/frequency/electrode points that were also significant in go trials alone. Similarly, for the [wait–go] contrast, the whole-brain activations were thresholded based on those time/frequency/electrode points that were also significant in the wait trials. This enabled the difference maps to be constrained to identify patterns of activation that were significantly related to a trial type in question. We performed this step during the initial stages of identifying significant patterns of activation that seemed most relevant to the behavior at hand (i.e., as a means of screening out significant patterns in the contrast that may be less relevant to the behavior in question).

We calculated the mean analytic amplitude for each electrode across a specified time window and frequency band of interest (described in paper for different analyses). The mean/SEM of that data were calculated across sessions, and we used a one-sample, two-sided *t* test (again compared with the null hypothesis that Z = 0) to evaluate significance of the means. We applied a Bonferroni correction to the *p* values from this data (across 32 comparisons if only performed at one frequency band; or across 32*5 if performed across all five frequency bands, as described in results). Results from these follow-up analyses were reported in Extended Data, and were also performed (without correction) at the level of animals (11 animals).

#### Weighted phase-lag index (wPLI) analysis

wPLI was calculated as described in prior studies (Stam et al., 2007; Vinck et al., 2011; Bastos and Schoffelen, 2016), using the Fieldtrip analytic toolbox. The w set of functions was used to calculate the evoked wPLI. We computed the cross-spectrum *C*(*f*) = *X*(*f*)Y*(*f*), of two real signal of x and y, which X and Y are Fourier transform x and y, and * indicates the complex conjugate. Then WPLI was computed using the magnitude of the imaginary component by the following Equation 1:

$$WPLI=\frac{\left|E(ℑ(Z))\right|}{E(\left|\left(ℑ\left(Z\right)\right)\right|)},$$

where Z indicates the complex nondiagonal part of *C* . wPLI was computed within each behavioral session separately for each trial type (go correct, wait correct, wait incorrect). As with the TF analyses, because we were primarily interested in changes in connectivity that occur during the task, we subtracted wPLI estimated from a baseline period before the visual stimulus (similar to the TF analyses) before performing statistical analyses. Statistical analysis was identical to that performed for TF analyses: one-sample *t* test at each TFE point, followed by FDR-correction across the entire 3D TFE matrix, with the null hypothesis being a wPLI = 0.

#### Electrode visualization

Visualization of electrodes was loosely organized based on prior evidence suggesting involvement in cognitive/behavioral operations relevant to the task: cognitive control [dorso-medial and ventro-mPFC, dorsomedial striatum, mediodorsal thalamus (MDT), central thalamus], sensorimotor motor [M1, M2, anterolateral motor cortex (ALM), FOFs (including A24a, A24b, A33), and dorsolateral striatum (DLS)], reward processing (OFC, nucleus accumbens, ventromedial striatum, and basolateral/central amygdala), visual processing (visual cortex), and putative default-mode regions [hippocampus (CA1/CA3), dentate gyrus, medial septum, parietal cortex, and retrosplenial cortex (A29c/A30c); Fig. 1].

#### Logistic regression/machine learning models

Logistic regression was performed using the MATLAB function glmfit across sessions. We first tested a univariate regression model comparing θ frequency band (4–7 Hz) activity averaged from correct versus incorrect wait trials at every TF point for each electrode shown (11 electrodes). The mean activity for correct wait and incorrect wait trials from each session was used as the dependent variable and the trial-type (correct or incorrect) as the independent variable. The output was FDR-corrected across times and electrodes. We next performed a similar analysis on the wPLI data. For the wPLI data, we first averaged the wPLI from each pair-wise region across time (500–900 ms poststimulus) and frequency (θ band). We then performed a regression analysis, as described above, for each pair-wise relationship. This analysis was also FDR-corrected across the entire 32 × 32 matrix. We displayed a select group of brain regions (11) selected because they showed high θ power/wPLI. This analysis was followed up with a multivariate logistic regression analysis, performed for the brain region with the highest univariate relationship to behavior (M1), using connectivity with the other 10 brain regions selected. Finally, to complement the multivariate analysis, we treated the identification of incorrect wait and correct wait trials as a classification problem and used a linear support-vector model (SVM) approach, applied at each time point across the trial (from –500 to 2000 ms from stimulus onset). We performed two forms of cross-validation for this. We used a 75/25 split of the data for training/testing, with 10-fold cross validation to estimate accuracy of each SVM model. This was repeated 10 times (with a different random 75/25 split of the data) to estimate the mean/SEM displayed in results.

#### Histology

At completion of recording sessions wire tips were marked by passing 12-μA current for 10 s through each electrode (Nano-Z, Neuralynx; Extended Data Fig. 1-1*B*). Rats were killed under deep anesthesia (100 mg/kg ketamine, 10 mg/kg xylazine, i.p.) by transcardiac perfusion of physiological saline followed by 4% formalin. Brains were extracted and immersed in 4% formalin for 24 h and then stored in 30% sucrose 4% formalin until ready to be sectioned. Tissue was blocked in the flat skull position, and sectioned frozen in the coronal plane at 50 μm. Brain slices of interest were Nissl stained using thionin to identify the course of the electrode tracks. Coarse electrode tracks (location of entire bundle of wires) were easily visualizable, although specific DV sites for each electrode within the bundle were less clearly visualizable. Because the DV sites were fixed before implantation, however, bundle location was used to make sure that electrodes were at least within the approximate AP/ML location.

### Human methods

#### Task

Participants accessed a game-like task structured similarly to the animal task. The basic task framework was modeled after the standard test of variables of attention (Greenberg and Waldman, 1993). In this two-block task, visual stimuli of colored rockets appeared in either the upper or lower central visual field. The task sequence consisted of a central fixation “+” cue for 500 ms, followed by a rocket stimulus of either blue target color or other iso-luminant nontarget color, presented for 100 ms. For blue rocket targets, participants were instructed to press the spacebar on the laptop keyboard as quickly as possible (go trials). For non-target color rockets (iso-luminant brown, mauve, pink, purple, teal), the participant was instructed to withhold their response until the fixation + cue flashed briefly on the screen, at 2 s for 100-ms duration (wait trials). Response feedback was provided for accuracy as a smiley or sad face emoticon presented 200 ms postresponse for 200-ms duration, followed by a 500-ms intertrial interval (ITI). Both task blocks lasted 5 min and consisted of 90 trials per block with 30/60 target/nontarget ratio in block 1 and 60/30 ratio in block 2 (blue rockets). Stimuli were presented in a shuffled order. Four practice trials preceded the first task block, and participants received a percent block accuracy score at the end of each block with a series of happy face emoticons (up to 10). All other assessments described below also used the same trial and block summary emoticon feedback specifications as in this task, to promote task engagement.

#### Subjects

Sixty-six adult human subjects (mean age 24.5 ± 7.3 years, range 18–53 years, 41 females) participated in the *BrainE* neurocognitive assessment study. They had no current diagnosis for a psychiatric disorder and/or current/recent history of psychotropic medications. All participants provided written informed consent for the study protocol (#180140) approved by the local institutional review board (IRB). All participants reported normal/corrected-to-normal vision and hearing and no participant reported color blindness. Approximately 95% of them were right-handed.

#### EEG

EEG data were collected simultaneous to the go–wait cognitive flexibility processing assessment using a 24-channel SMARTING device with a semi-dry and wireless electrode layout following standard 10/20 system. Data were acquired at 500-Hz sampling frequency at 24-bit resolution. Cognitive event markers were integrated using LSL and data files were stored in xdf format.

#### Behavioral analysis

Data were analyzed for each type of stimulus, i.e., go and wait. For each stimulus, signal detection sensitivity was computed as d’ = *z*(hits) – *z*(false alarms) (Heeger and Landy, 2009). All d’ values were divided by max theoretical d’ of 4.65 to obtain scaled d’ in the 0–1 range. Response times (RTs) are reported in seconds.

#### Neural analysis

We applied a uniform processing pipeline to all EEG data acquired simultaneous to the cognitive tasks (Balasubramani et al., 2020). This included: (1) data preprocessing, (2) computing event-related spectral perturbations (ERSPs) for all channels, and (3) cortical source localization of the EEG data filtered within relevant δ (1–4 Hz) and θ (4–7 Hz) frequency bands. (1) Data preprocessing: data preprocessing was conducted using the EEGLAB toolbox in MATLAB (Delorme and Makeig, 2004). EEG data were resampled at 250 Hz, and filtered in the 1- to 45-Hz range to exclude ultraslow DC drifts at <1 Hz and high-frequency noise produced by muscle movements and external electrical sources at >45 Hz. EEG data were average referenced and epoched to the emotional task stimuli as informed by the LSL time stamps in the −1.5 s to +1.5 s stimulus time window. Epoched data were cleaned using the autorej function in EEGLAB to remove noisy trials (>5sd outliers rejected over max 8 iterations; 8.1 ± 5.1% of trials rejected per participant). EEG data were further cleaned by excluding signals estimated to be originating from non-brain sources, such as electrooculographic, electromyographic or unknown sources, using the sparse Bayesian learning (SBL) algorithm (https://github.com/aojeda/PEB) explained below (Ojeda et al., 2018, 2019). (2) ERSP calculations: we performed TF decomposition of the epoched data using the continuous wavelet transform (cwt) function with the analytic Morlet (Gabor) wavelet in MATLAB’s signal processing toolbox. Baseline TF data in the −750- to −550-ms time window before stimulus presentation were subtracted from the epoched trials (at each frequency) to observe the event-related synchronization (ERS) and event-related desynchronization (ERD) modulations (Pfurtscheller, 1999). Here, we computed the sign of ERSP differences between go and wait stimuli, and they were statistically corrected across subjects using *t* test (*p* < 0.05). We used only correct accuracy trials for this finding. (3) Cortical source localization: cortical source localization was performed to map the underlying neural source activations for the ERSPs using the block-SBL algorithm (Ojeda et al., 2018, 2019) implemented in a recursive fashion. This is a two-step algorithm in which the first-step is equivalent to low-resolution electromagnetic tomography (Pascual-Marqui et al., 1994). LORETA estimates sources subject to smoothness constraints, i.e., nearby sources tend to be co-activated, which may produce source estimates with a high number of false positives that are not biologically plausible. To guard against this, SBL applies sparsity constraints in the second step wherein blocks of irrelevant sources are pruned. Source space activity signals were estimated and the root mean square signals were partitioned into cortical ROIs and artifact sources. ROIs were based on the standard 68 brain region Desikan–Killiany atlas (Desikan et al., 2006) using the Colin-27 head model (Pascual-Marqui et al., 1994). Activations from artifact sources contributing to EEG noise from non-brain sources such as electrooculographic, electromyographic or unknown sources, were removed to clean the EEG data. Cleaned subject-wise correct trial-averaged EEG data were then specifically filtered in δ and θ frequency bands, separately source localized in each of the frequency bands, and baseline subtracted to estimate their band-specific cortical ROI source signals. The source signal envelopes were computed in MATLAB (envelop function) by a spline interpolation over the local maxima separated by at least one time sample; we used this spectral amplitude signal for all neural analyses presented here. We focused on time periods between 350 and 650 ms on wait trials (θ activity) and 200–300 ms poststimulus for identifying go activity in δ frequency bands. Go > Wait and Wait > Go neural activations were compared across subjects using *t* tests (*p* < 0.05). 4) WPLI analysis were performed for the correct go and wait trials and their contrast in δ and θ frequency bands was FDR-corrected similar to the rodent data methods to evaluate significance of the wPLI.

## Results

**Figure 2.**
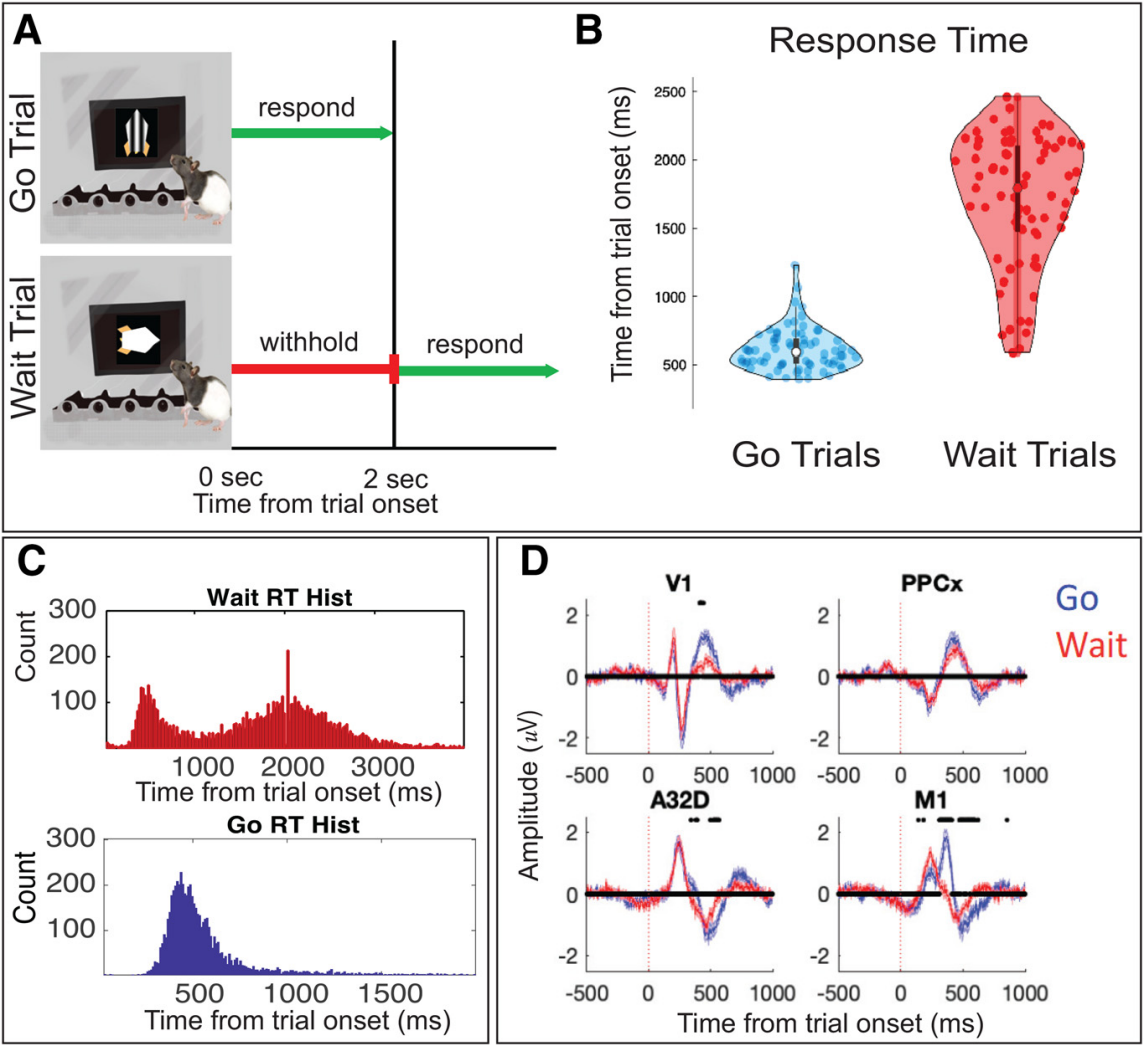
Task design and electrophysiological approach. ***A***, The behavioral paradigm we used consisted of a visual stimuli instructing animals to respond immediately (go trial, denoted with an upward-facing striped rocket) or after specified delay (wait trial, denoted with a horizontal-facing white rocket). On go trials, animals had to respond within 2 s to earn a reward (20 μl of water). On wait trials, animals had to wait 2 s and then respond to collect a reward. ***B***, A violin plot representation of the RT for go and wait trials across behavioral sessions (single dots represent individual behavioral sessions across 60 sessions). Animals could distinguish visual stimuli and showed appropriate behavior (waiting longer on wait compared with go trials). ***C***, Histogram of all trials (from all sessions), demonstrating the difference in reaction time for wait and go trials, showing clear discrimination and attempts to wait. ***D***, Average event-related potentials (ERPs) from a few selected brain regions: V1, M1, PPCx, and dmPFC from go and wait trials (*n* = 60 sessions). Data were broad-band filtered (0.5–500 Hz), and baseline normalized before averaging. Shaded-error plots show mean/SEM. There are significant differences between ERPs for go (blue) and wait (red) trials; *significant differences at *p* < 0.05, as estimated using a paired *t* test, followed by FDR-correction performed across the entire 2D time and electrode matrix. Extended Data Figure 2-1 shows preprocessing steps, number of trials/session, and a different view of electrode placement along histology.

Task-structure (Fig. 2*A*) is described in the methods. In sessions used for physiological analysis in this study (i.e., those with good LFP recordings) accuracy was 97 ± 1% correct on go trials and 54 ± 12.4% on wait trials, with an average d’ of 1.67 across behavioral sessions. This task was self-paced: animals had to enter a NP to trigger the visual stimulus denoting the start of the trial. The mean RT, calculated as the time between the onset of the visual stimulus and the animal’s subsequent response, was 588 ± 15 ms on go trials and 1770 ± 55 ms on wait trials (*p* < 0.001, paired *t* test between trial-types, *n* = 60 sessions/11 animals; Fig. 2*B*). The overall distribution of wait trial times across all trials (Fig. 2*C*) demonstrates a bi-modal distribution of trials (i.e., incorrect waiting may occur because of incorrect decision-making as well as inability to wait the full 2 s). Animals showed a strong bias toward responding and have difficulty waiting the full 2 s. While the analyses in this paper focus on TF decompositions of brain activity gathered from different brain sites, we first characterized average, broad-band (0.3–500 Hz) event-related potentials from four selected brain regions on go trials: V1, M1, dmPFC (A32D), and posterior parietal cortex (PPCx). Each region showed clear, temporally distinct event-related potentials. Large differences in the evoked response for go versus wait trials occur within M1 (Fig. 2*D*, significant time points between trial time points marked on figure with stars, *p* < 0.05 FDR-corrected), while more subtle differences between trial types were observed in V1 and A32D. Parietal cortex showed similar activity patterns. This analysis of the evoked potentials demonstrates that different electrodes show distinct patterns of activity during the task associated with different behaviors (go vs wait).

### Low-frequency activity in motor circuits associated with action

**Figure 3.**
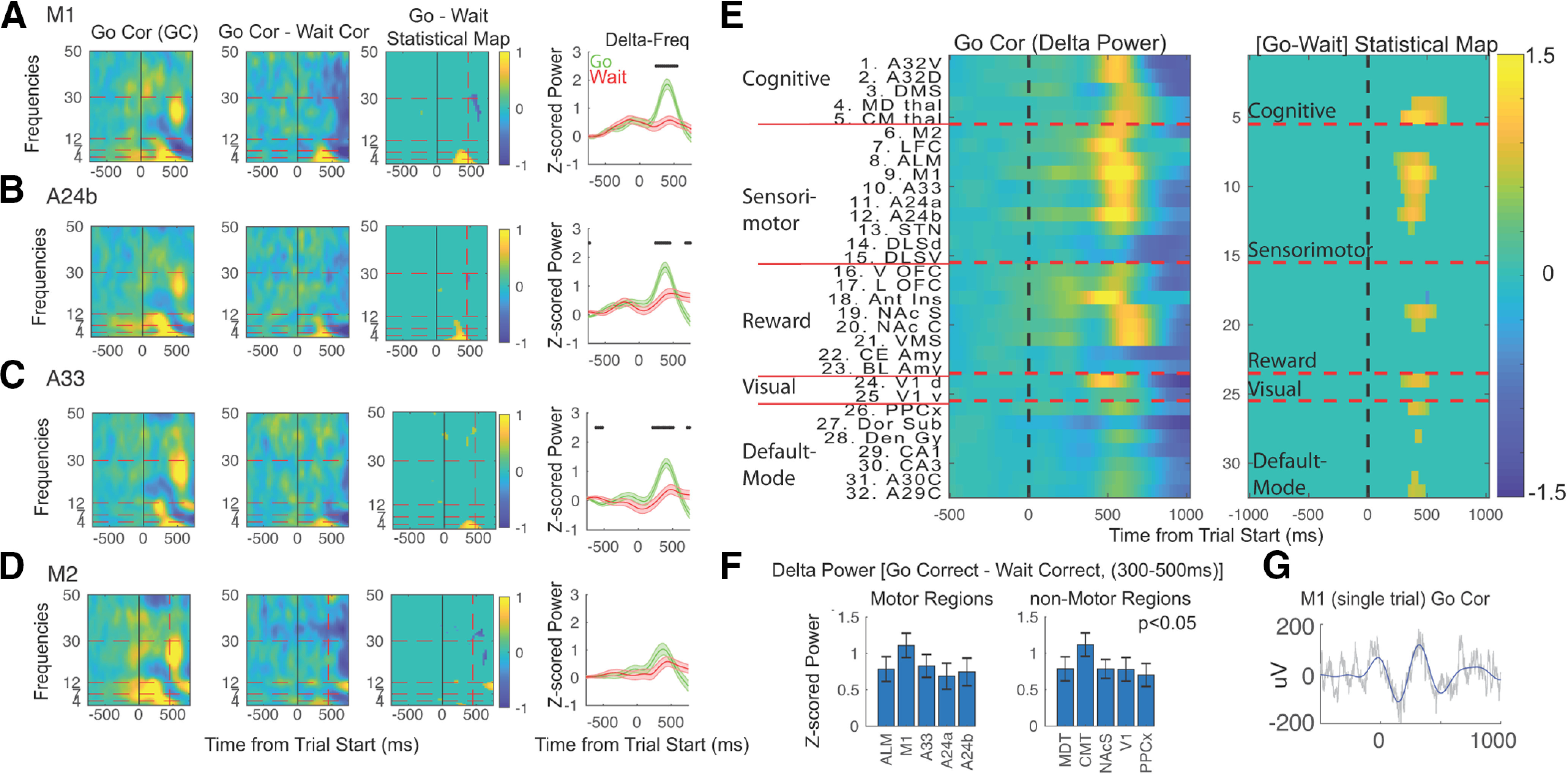
Low-frequency activity linked with sensory-evoked responses. Activity in three key sensory-response mapping regions was investigated. ***A–D***, TF activity from four motor regions was plotted: M1, parts of the FOF (A24b, A33), and anterior portion of M2. For each region we estimated average activity for correct go trials and the difference from correct go and correct wait trials (*p* < 0.05, FDR-corrected across all electrodes, times and frequencies). The third column shows the significant [go–wait] contrast at TF points that are also significant on go trials alone (statistical thresholded maps, FDR-corrected for each analysis separately before thresholding). Finally, for each region, we also plotted the mean/SEM trace within the δ frequency range for go (green) and wait (red) trials (time points with significant differences between the two denoted with *). ***E***, Mean δ frequency activity across brain regions for go correct trials, and the statistically thresholded contrast [go–wait] map (thresholded for significant time points in the contrast that are also significant on go trials alone). Significant activations were observed in sensorimotor regions (ALM, M1, A33, A24a, A24b), along with thalamus, visual cortex, sensorimotor cortex, and NAcS. ***F***, Mean/SEM of [go–wait] contrast averaged across δ frequencies from 300 to 500 ms poststimulus. All regions displayed are highly significant (adjusted *p* < 0.05, Bonferroni-correction for 32 electrodes). Extended Data Figure 3-1 includes extended mean/SEM and adjusted *p* values for all electrodes, and Extended Data Figure 3-2 shows results when calculated at the level of animals. ***G***, Single trial example of δ activity from M1 to demonstrate what task-evoked δ oscillations looks like.

Our first set of analyses was focused on identifying physiological activity linked with immediate actions (i.e., those related to the go cue). One challenge with this multidimensional data were to isolate activity associated specifically with cued-actions. We took the following approach: we measured TF activity gathered from each electrode for go-trials, and for the contrast between go and wait trials (Fig. 3*A****–****D*, first two panels). We then used two procedures to isolate cued-action related activity (Fig. 3*A–D*, third panel). First, we applied a *t* test on both the “go-correct” and the “go–wait difference” maps across all times/frequencies/electrodes, each of which was then FDR-corrected. We used the significance maps from both analyses to mask the [go–wait] contrast. The resulting activations shown were thus significant for both analyses. This approach (using both go and the go – wait contrast to filter results) introduces certain biases in our results, but we believe it is helpful in revealing electrophysiological activity with the strongest connection to the behavior in question.

10.1523/ENEURO.0406-20.2021.f3-1 Extended Data Figure 3-1 Mean activity from all brain regions filtered in delta frequencies (1-4 Hz) within the time window from 300-500ms post stimulus. p-values listed came from a two-sided, one-sample t-test, with null hypothesis (0), followed by Bonferonni adjustment of p-values (32 regions). Adjusted p-values > 1 were automatically set to 1. Data was estimated at the level of sessions (60). Download Figure 3-1, DOCX file.

10.1523/ENEURO.0406-20.2021.f3-2 Extended Data Figure 3-2 Mean activity from all brain regions filtered in delta frequencies (1-4 Hz) within the time window from 300-500ms post stimulus. p-values listed came from a two-sided, one-sample t-test, with null hypothesis (0). Data was estimated at the level of animals (n=11). Download Figure 3-2, DOCX file.

This approach allowed us to identify low-frequency activity (δ and θ) within specific motor regions as associated with cued-actions (i.e., it was significantly active during go-trials and it was significantly greater for go versus wait trials (Fig. 3*A–D*, last two panels). Motor cortex (including both primary and secondary motor areas) in rodents overlaps with sensory cortex and spans a vast area from at least –1 mm posterior to bregma to 4 mm anterior to bregma, and from 1 to 5 mm lateral to bregma. We implanted electrodes in many different parts of this large area (Fig. 1*A*). Anterior motor areas targeted (3.75 mm anterior from bregma) included M2 and anterior part of M1 (we call this region antero-lateral motor cortex, or ALM) and lateral frontal cortex (an amorphous area between M1 and insula). In addition, we implanted electrodes in a posterior part of motor cortex (2 mm anterior to bregma) that included the FOFs (a part of rodent secondary motor area thought to be involved in sensory-response mapping, comprised of A24a, A24b, A33; Vogt and Paxinos, 2014; Barthas and Kwan, 2017) and M1 (Ramanathan et al., 2006). Many parts of the motor system (examples shown include M1, A24b, A33) showed action-specific activity, while a few (e.g., M2) did not (Fig. 3*A-D*). Across the brain, we found significant action-related δ frequency activity in multiple brain regions (Fig. 3*D*). To summarize results across time/frequencies, we calculated the mean/SEM difference between go and wait trials within the time-window from 300 to 500 ms after the stimulus (when the peak activation occurred for most brain regions) within δ frequencies for the brain regions noted above (we used a one-sample, two-tailed *t* test with a null hypothesis that [go–wait] difference = 0, followed by FWE-adjusting of *p* values; Fig. 3*E*; Extended Data Fig. 3-1 for all regions). Sensorimotor regions showing significant action-related δ activity differences for go compared with wait included M1 (mean evoked contrast 1.1 ± 0.17, *p* = 4.8e-7), ALM (mean evoked contrast 0.78 ± 0.17, p = 8e-04; and parts of M2/FOFs (Brecht, 2011; Barthas and Kwan, 2017), including A24A (mean 0.68 ± 0.18, *p* = 0.01), A24B (mean 0.75, ±0.16, *p* = 0.008), and A33 (mean 0.83, ±0.16, *p* = 7.9e-5). Other regions also showed significant δ frequency contrasts for go compared with wait trials including: V1 (mean 0.78, ±0.16, *p* = 3e-4), PPCx (mean 0.7, ±0.156, *p* = 0.001), MDT (mean 0.79, ±0.17, *p* = 5e-4), centromedian thalamus (CMT; mean 1.1, ±0.16, *p* = 1.2e-7), and NAcS (mean 0.79, ±0.13, *p* = 4.2e-6).To ensure that results were not driven solely by high sample size achieved by performing statistics at the level of sessions, we confirmed that most of these results were still significant when performed at the level of animals, although without FWE adjustment (Extended Data Fig. 3-2).

**Figure 4.**
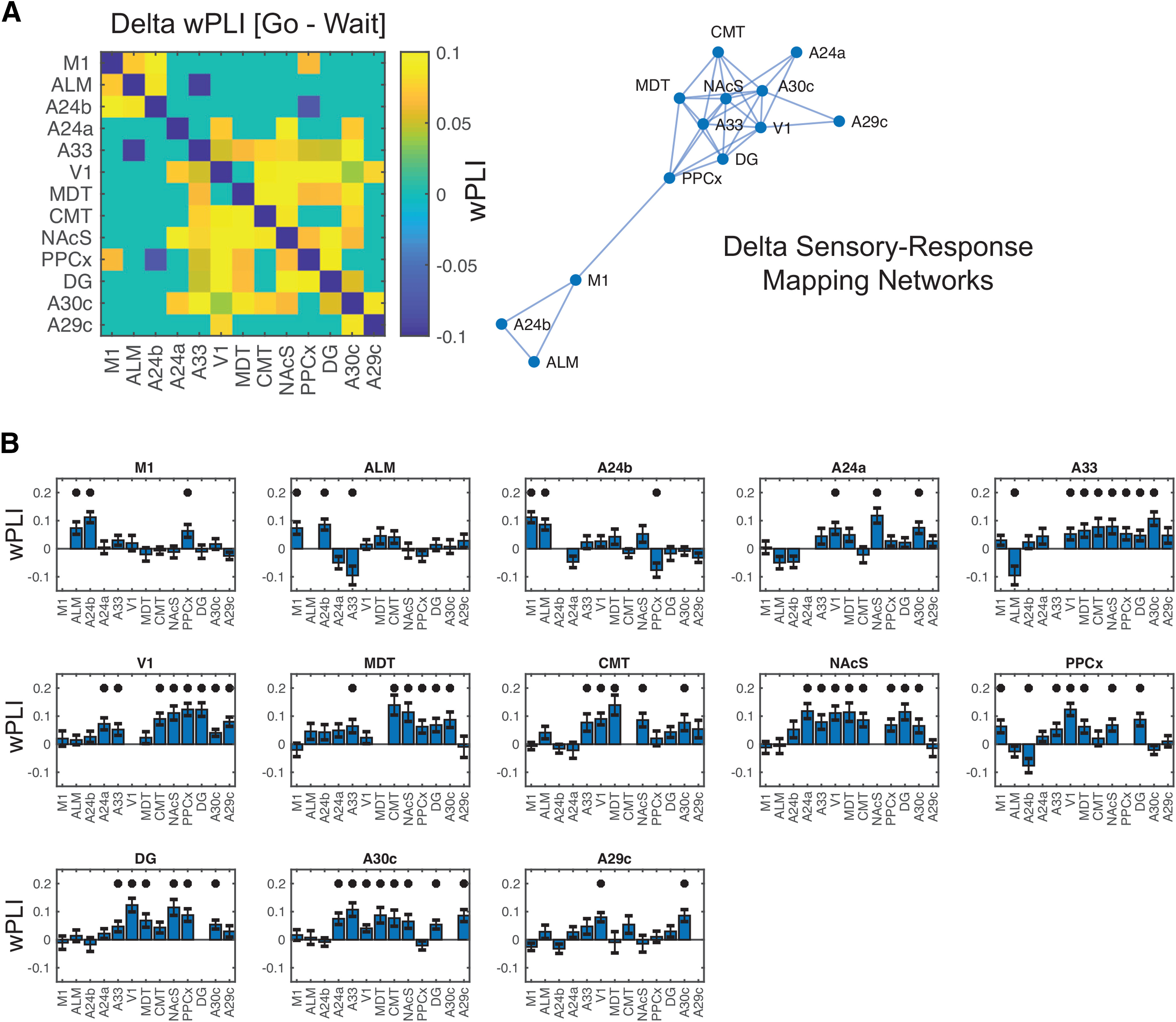
Low-frequency wPLI associated with action. The difference between go and wait trials in wPLI was calculated at δ frequencies, averaged over the period between 300 and 500 ms (analysis restricted to brain regions showing significant δ activity during this time period). ***A***, We found increased connectivity (wPLI) in two distinct subnetworks at δ frequencies during this time period for go compared with wait trials. The first was a distinct motor network comprising of M1 (ALM) and a dorsal part of the FOFs (A24b). The second network comprised of sensory, reward and memory brain regions. Graph of the significant connections on the right. ***B***, Bar-plots showing [go–wait] wPLI contrast between 300 and 500 ms poststimulus; *significant regions (FDR-correction applied across 11 × 11 matrix). Extended Data Figure 4-1 includes extended mean/SEM and adjusted *p* values for all electrodes. All bars show mean/SEM.

Common low-frequency activity observed across multiple brain regions could be a reflection of activity in one source, with volume conduction explaining the spatial spread. To better understand the degree to which this activity formed functional networks (instead of being driven by volume conduction), we estimated the wPLI between brain regions in a pair-wise fashion at δ frequencies. wPLI (Stam et al., 2007; Hardmeier et al., 2014; Cohen, 2015; Bastos and Schoffelen, 2016) is a conservative measure of functional connectivity that estimates the consistency of the non-zero phase-lag between two time series, and further weights this estimate by the magnitude of the imaginary coherence (thereby suppressing phase-lags that are close to zero, which show low values). This analytic method is known to be one of the most conservative approaches for measuring functional connectivity, and seems to be robust against volume-conduction (Vinck et al., 2011; Lau et al., 2012; Hardmeier et al., 2014). To measure the event-related wPLI during the task (mimicking the approach we take for ERSPs), we subtracted the baseline wPLI (calculated 700–500 ms before trial onset). We next calculated the difference in this baseline corrected wPLI between correct go and correct wait trials from 300 to 500 ms after the stimulus (the peak time-window for δ power) for the 13 regions noted above showing significant δ power (Fig. 4; Extended Data Fig. 4-1). The illustrated graph depicts the network using significance-value thresholds. This analysis demonstrated the presence of two functional networks associated with cued actions. The first was a motor network involving M1 and two anatomically distinct secondary motor regions: ALM and A24b. The second involved regions that appear to be associated with sensory-response mapping, and included NAcSh, V1, PPCx/retrosplenial cortex, and thalamus. Interestingly, these brain regions were highly interconnected with certain key nodes of the FOFs (particularly A33) and the entire network was connected with motor circuits at least in part through PPCx (wPLI and statistical tests shows in Extended Data).

10.1523/ENEURO.0406-20.2021.f4-1 Extended Data Figure 4-1 We calculated the pair-wise weighted phase-lagged-index from the above regions on at a session by session level for both correct wait and correct go trials. We then estimated the mean/SEM of the pair-wise wPLI difference at delta frequencies (1 -4 Hz) from 300-500ms post-stimulus onset. p-values were calculated using a one-sample two-tailed t-test on this difference (null hypothesis is that there was no difference). P-values were then adjusted using an FDR-correction. Download Figure 4-1, DOCX file.

**Figure 5.**
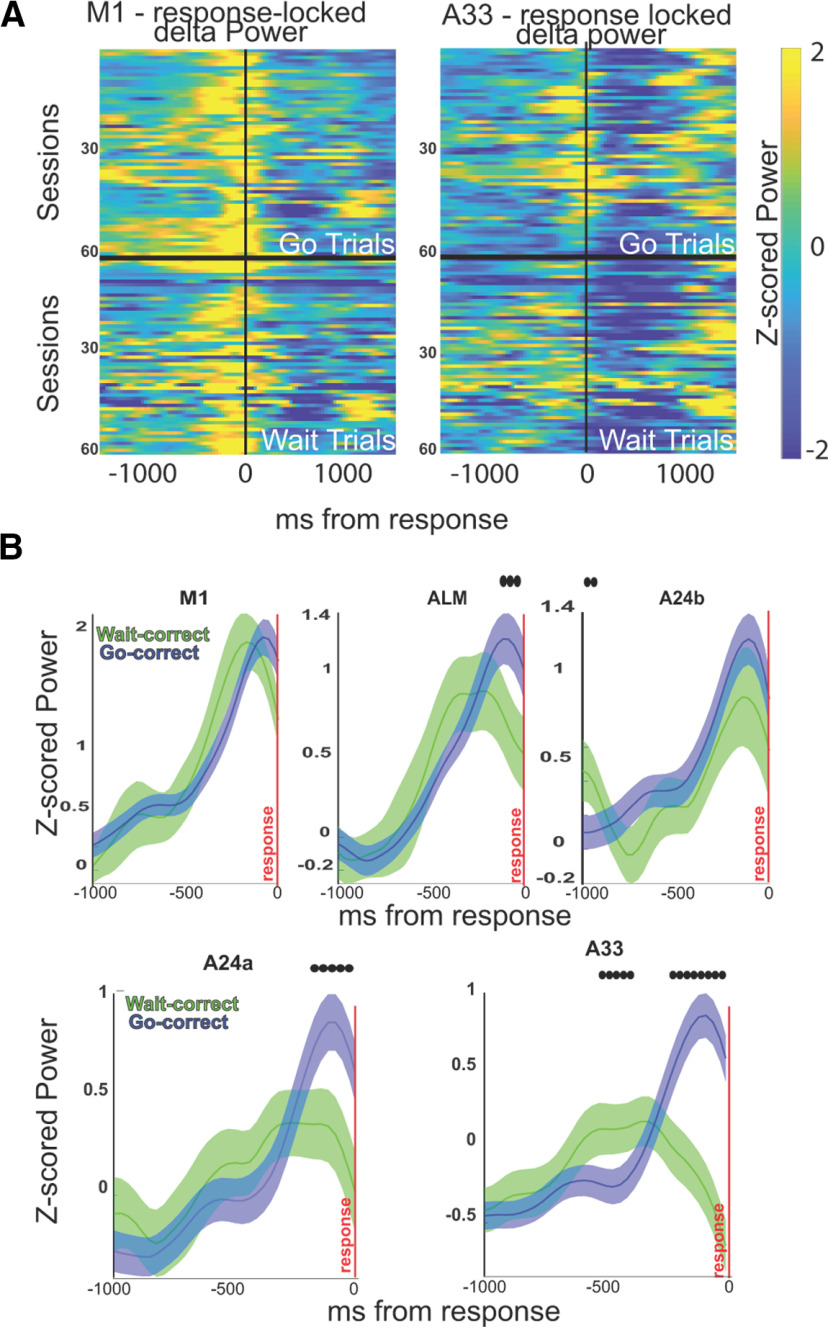
Response locked low-frequency activity differentiates motor brain regions. ***A***, Low-frequency activity from go and wait trials, time-locked to the response, is shown for two motor brain regions: M1 and A33. Average activity shown from each session, contrasting the go and wait responses. M1 showed qualitatively similar low-frequency activations preceding the response. A33 shows generally stronger low-frequency activity preceding the responses for go compared with wait trials. ***B***, Mean/SEM traces of the low-frequency activity for five motor regions involved in action displayed (M1, ALM, A24b, A24a, A33). A24a and A33 showed clearly larger differences in low-frequency activity for the delayed compared with immediate action trials. Significance marked, *p* < 0.05, FDR-corrected across time points.

Thus, despite similar appearing δ frequency activity observed across multiple brain regions, the wPLI data suggests that this δ activity segregates into two functional systems, a “motor” related one and a “sensory-response-mapping” region. If this is the case, we hypothesized that M1, ALM, and A24b may be involved, generally, in both cued and delayed motor actions, while deeper parts of the FOF (A24a, A33 in particular) which are highly interconnected with sensory/attention brain regions, would be more specifically involved in cued motor actions and consistent with a putative role in sensory-response mapping. To test this, we time-locked δ frequency activity to the response and estimated δ power before the response for both trial types (correct responses only) for each of the five sensorimotor regions noted. Across sessions we observed fairly similar response-related M1 activity for both go and wait-trial actions (Fig. 5*A*). By contrast, activity from A33, a key node of the sensory-response mapping network, showed strong δ activity for immediate, cue-related go actions but less strong activity on the delayed (wait-trial) responses (Fig. 5*A*,*B*). We found a clear difference between go versus wait trials within the 200-ms window before actions for A33 (*p* = 2 × 10^–6^, FWE-corrected for five regions) and almost significant results for another part of FOF, A24A (*p* =0.08, FWE-corrected for five regions). The other motor brain regions showed relatively similar action-related activity for immediate and delayed actions (M1, *p* > 0.9; ALM, *p* = 0.3033; and A24B, *p* > 0.9). Thus, the wPLI network-based analysis and event-locked TF analysis both indicate that motor brain regions segregate into two distinct low-frequency networks involved in different aspects of motor functioning: a “sensory-response” mapping network involving ventral portions of the FOF and a more general motor network involving M1 and more dorsal parts of the FOF.

### Distributed θ-based inhibition network

**Figure 6.**
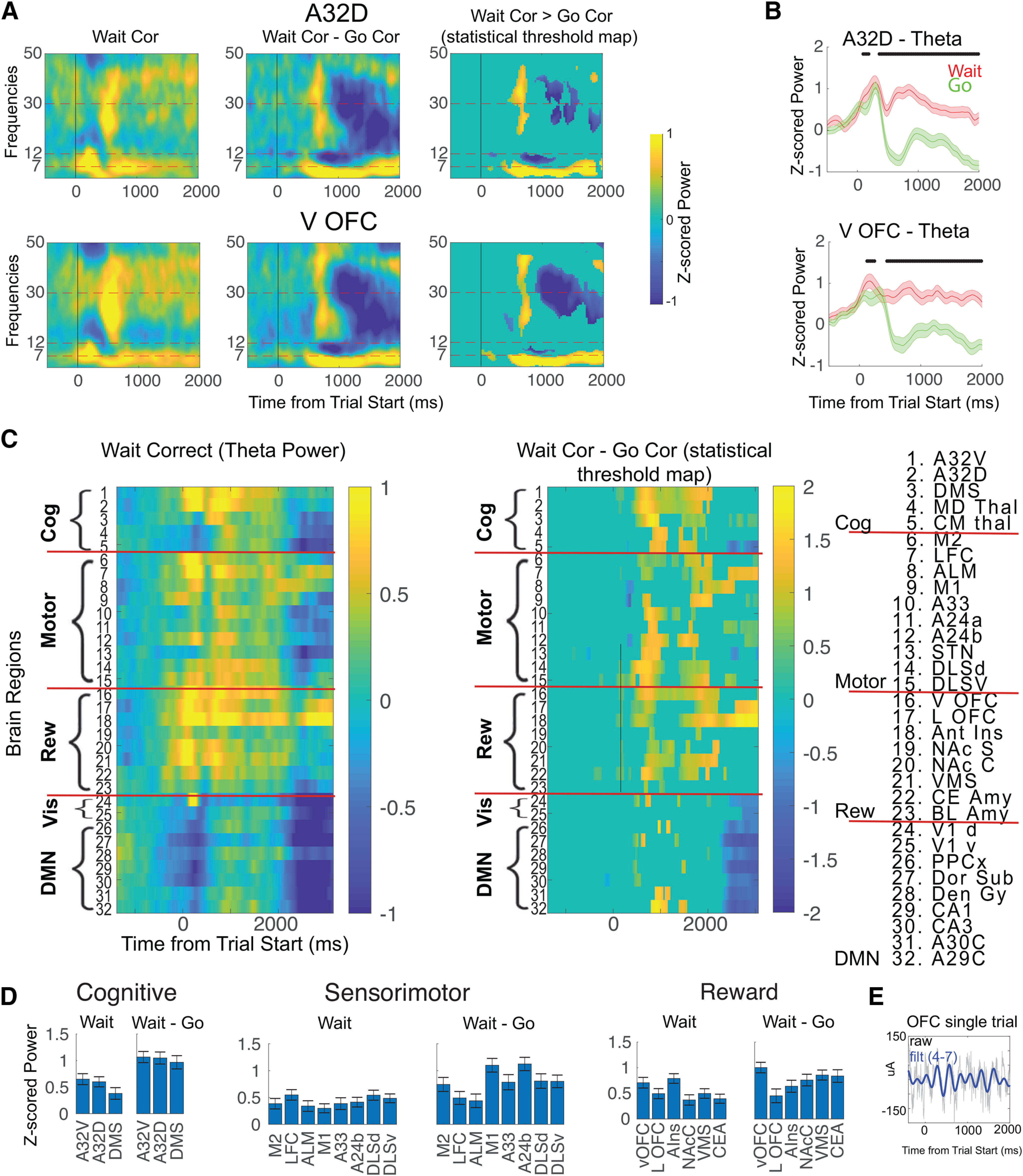
θ Oscillations linked with behavioral inhibition. ***A***, ***B***, TF activity was plotted for two brain regions previously linked with inhibition: A32D and vOFC. Mean activity from wait-trials (left panel) and from the wait–go contrast was followed by the statistical thresholded [wait–go] contrast map (thresholded for significant activations for go trials alone and a positive difference for the wait–go contrast, both FDR-corrected). The statistically thresholded map shows greater θ activity for wait compared with go trial types (FDR-corrected, *p* < 0.05, with non-significant points set to 0). Line plots of the mean/SEM activity in the θ frequency band was displayed for go and wait trials, showing time points where the two regions have significantly different θ activity for wait compared with go trials. ***C***, Whole-brain map of θ related to waiting, showing both mean wait-trial θ activity, and the significant, thresholded maps for wait–go (thresholded for significant θ activity for both wait trials and for the wait–go difference). ***D***, Mean/SEM of the θ activity averaged across the 500- to 2000-ms window. All brain regions shown have significant activity for both wait trials and for the [wait–go] contrast (*p* < 0.05, FWE-corrected across 32 electrodes). ***E***, Example trace of theta-filtered activity from OFC. Extended Data Figure 6-1 includes extended mean/SEM and adjusted *p* values for all electrodes, and Extended Data Figure 6-2 shows results when calculated at the level of animals.

We used a similar analytic approach to identify patterns of brain activity associated with waiting/action postponement. We started by analyzing activity in ventral OFC (vOFC) and dorsomedial PFC (A32D), regions that have some prior association with behavioral inhibition and motor impulsivity. For each region we calculated the average TF activity for wait trials, for the wait–go difference, and the statistical significance map for the wait–go difference (Fig. 6*A*,*B*, thresholded by the FDR-corrected significance maps from both of the wait and wait–go analyses.). This analysis identified sustained activity in θ frequencies associated with inhibition. We additionally observed a burst of β activity between 500 and 700 ms. As it was brief, we did not fully perform further analyses on the β activity in this study, although it appeared roughly time-locked to when animals respond on go-trials, and so thus may reflect some aspect of decision or reward-prediction that occurs in both trials around that time period. The sustained waiting-specific θ activity was observable across many other brain regions as well (Fig. 6*C*, showing both activity on wait-trials, and the significantly thresholded contrast analysis as described above). This waiting-related θ activity was broadly distributed across cognitive, sensorimotor and reward-related brain regions. Regions with significantly greater θ activity for wait compared with go (and significant activity in wait trials alone) included (averaged across the 500- to 2000-ms time window, FWE-corrected for 32 regions) included dorsomedial PFC (A32D, *p* = 1.9e-11 for the wait–go contrast) and ventromedial PFC (A32V, *p* = 5.7e-13 for the contrast), vOFC (*p* = 2.1e-12 for the contrast), lateral OFC (lateral OFC, *p* = 4.8e-2 for the contrast), anterior insula (*p* = 3.8e-05 for the contrast), ventromedial striatum (*p* = 1.2e-10 for the contrast), nucleus accumbens core (*p* = 3.5e-07 for the contrast), and parts of amygdala (CEA, *p* = 2e-07 for the contrast; Fig. 6*D*; Extended Data Fig. 6-1 for full details on mean/*p* values). A number of motor regions also showed waiting-related θ activity, including M1 (*p* = 1.8e-10), M2 (*p* = 1.6e-05), LFC (*p* = 4.7e-03), and A24b (*p* = 3.8e-11). To ensure that results were not driven solely by high sample size achieved by performing statistics at the level of sessions, we confirmed that most results still held when performed at the level of animals (Extended Data Fig. 6-2).

10.1523/ENEURO.0406-20.2021.f6-1 Extended Data Figure 6-1 Mean theta power from electrodes for correct wait trials alone, and from the difference (correct wait trials - correct go trials), data taken from 500-2000ms post-stimulus. Mean/SEM calculated at the level of sessions (60 sessions). We used a one-sample, two-sided t-test for both analyses (null hypothesis that power = 0). p-values were adjusted for multiple corrections using Bonferroni adjustment (32 regions). Bold names are highlighted that are significant for both analyses, suggesting involvement in inhibition. Download Figure 6-1, DOCX file.

10.1523/ENEURO.0406-20.2021.f6-2 Extended Data Figure 6-2 Mean theta power from electrodes for correct wait trials alone, and from the difference (correct wait trials - correct go trials), data taken from 500-2000ms post-stimulus. Mean/SEM calculated at the level of animals (11 animals). We used a one-sample, two-sided t-test for both analyses (null hypothesis that power = 0). Bold names are highlighted that are significant for both analyses, suggesting involvement in inhibition. Download Figure 6-2, DOCX file.

**Figure 7.**
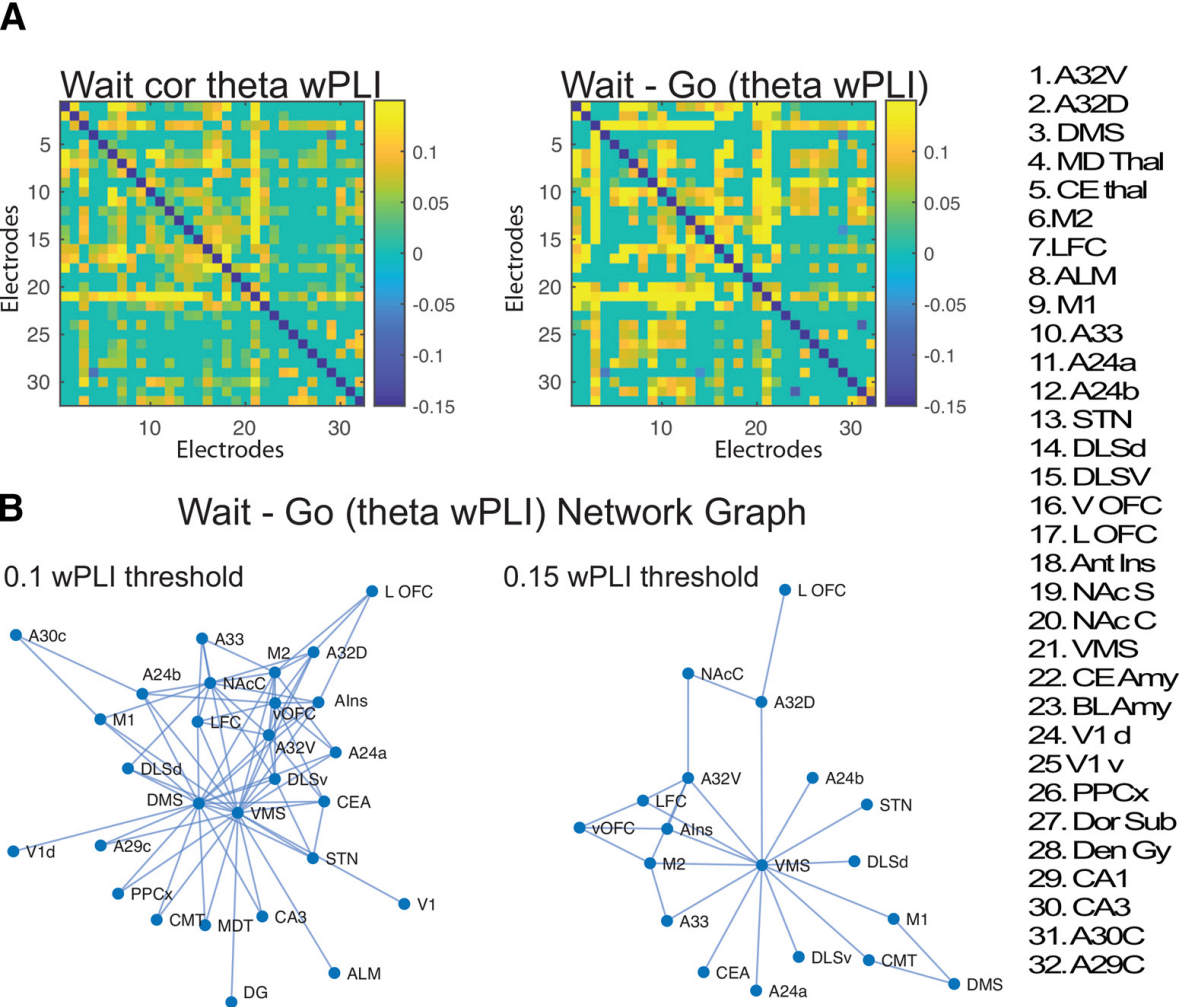
wPLI associated with behavioral inhibition. ***A***, We calculated the mean wPLI for each region to each other region for wait trials (thresholded using FDR-correction) and the [wait–go] difference (showing both the average wPLI and the FDR-corrected map, *p* < 0.05, with non-significant values set to 0). Extended Data Figure 7-1 plot the mean/SEM and *p* values for all electrodes. ***B***, Graph of the wait–go difference shown at two different network thresholds to illuminate the strongest pair-wise wPLI connections (all shown are significant). Extended Data Figure 7-2 plot the mean/SEM and *p* values for all electrodes.

We next conducted a wPLI analysis as a way to understand the degree to which this widespread activity represented functional networks or instead was a reflection of volume conduction artifact or truly represented widely distributed functional network. Our analysis was focused on the wPLI difference for correct wait compared with correct go trials. We calculated the average region-by-region wPLI during the waiting time period (500–2000 ms; Fig. 7*A*, *p* < 0.05, Bonferroni-corrected; Extended Data Figs. 7-1, 7-2**)**. This analysis demonstrated wide-spread wPLI at θ frequencies associated with inhibition. To better represent this, we graphed the [wait–go] network differences (using two thresholds, at 0.1 and 0.15 wPLI, with all connections shown significant).

10.1523/ENEURO.0406-20.2021.f7-1 Extended Data Figure 7-1 Data table for mean, SEM and p-values for pair-wise wPLI. Data taken at theta-frequencies between 500-2000ms post-stimulus. P-values adjusted for multiple comparisons using FDR-correction. Download Figure 7-1, DOCX file.

10.1523/ENEURO.0406-20.2021.f7-2 Extended Data Figure 7-2 Data table for mean, SEM and p-values for pair-wise wPLI for the [wait-go] contrast. Statistical test used was a one-sample t-test, mean Data averaged at theta-frequencies between 500-2000ms post-stimulus. P-values adjusted for multiple comparisons using FDR-correction. Download Figure 7-2, DOCX file.

### Brain connectivity correlations with successful waiting

**Figure 8.**
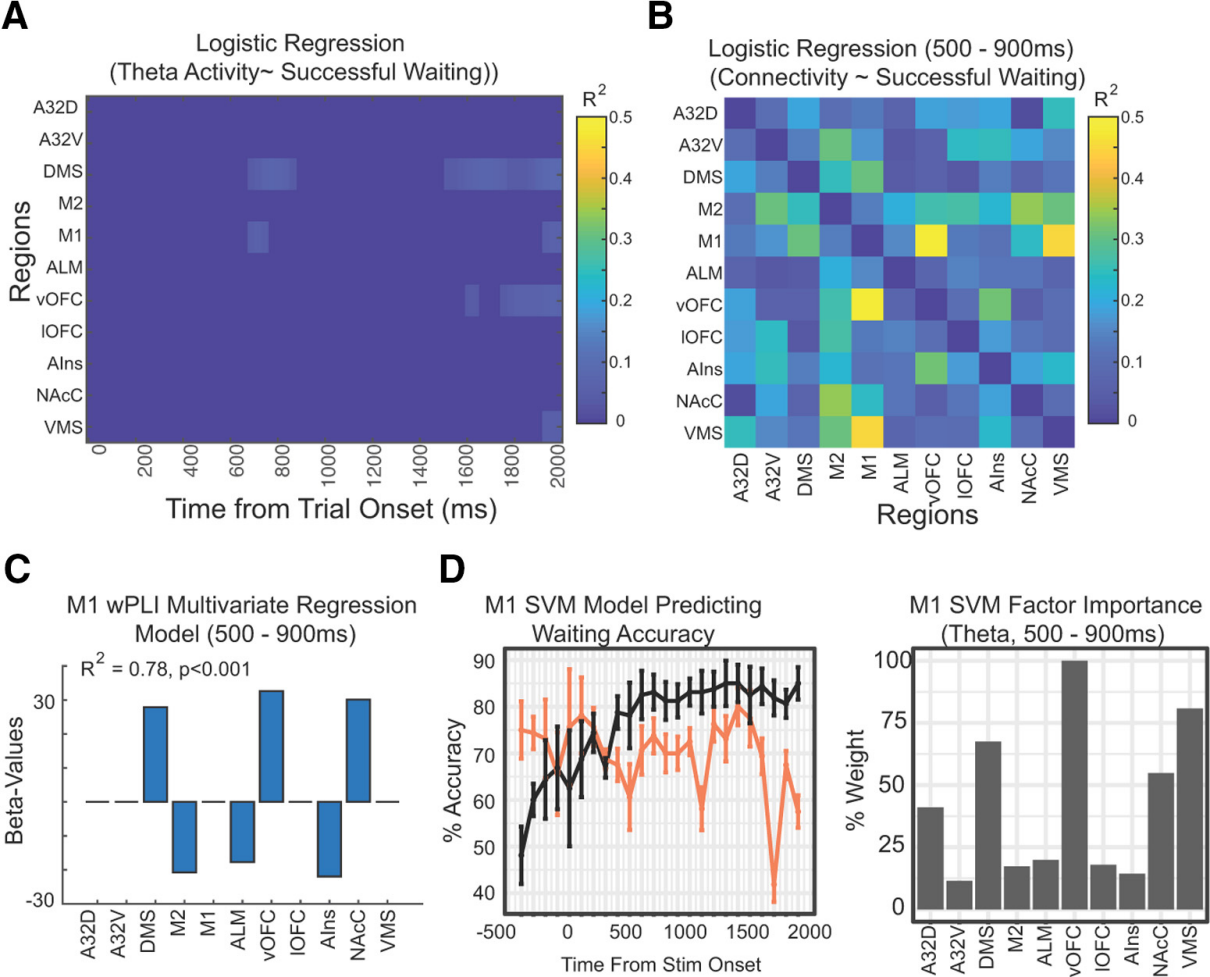
Correlation between network connectivity and successful inhibition. ***A***, Logistic regression was performed over time between θ activity (averaged for successful vs unsuccessful wait trials) and trial-type from each of the behavioral sessions (FDR-corrected/thresholded across all electrodes/time). We plotted activity in 11 selected regions showing strong θ activity or connectivity. We found significant activity in DMS and M1 correlated with successful inhibition but relatively weakly. ***B***, We performed a similar analysis using wPLI values averaged in an early period (between 500 and 900 ms after stimulus onset, FDR-corrected). We found highly significant and strong correlations with behavior using pair-wise connectivity. M1-OFC connectivity shows a strong and significant relationship with behavior. ***C***, Multivariate regression model was developed with connectivity from M1 to the 10 other brain regions (only significant β values in the model shown), demonstrating that connectivity with ventral striatum and OFC are associated with improved impulsivity while connectivity with motor regions are associated with diminished impulsivity. Overall, multivariate regression model shows a strong relationship with behavior, demonstrating that connectivity can be used to accurately classify trial types. ***D***, We next used an SVM ML model to measure classification of trial types using M1 connectivity at each time point for both θ and β activity. Model used a 75%/25% split (training vs test) with 10× cross-validation, and we randomized the initial 75/25 split ten times to produce a mean/SEM of the model. We performed this for wPLI from M1 for both theta (black) and Beta (orange) values. We found that the θ model achieves >80% performance by 500 ms poststimulus. We plotted the SVM Factor importance for the ML model (using time-points between 500-900ms post stim). We found connectivity values from vOFC, nAcC, VMS, DMS and A32D were particularly important in the prediction model.

A major goal of this study was to identify activity that was related to successful behavioral inhibition (i.e., waiting patiently for 2 s before responding). The average RT on incorrect (i.e., impulsive) wait trials was 1263 ± 290 ms, while the average RT on patient wait trials was 2513 ± 135. Based on the above results, we hypothesized that θ activity or θ wPLI connectivity between brain regions might correlate with this improved behavioral control. To test this we performed two logistic regression analyses, first with power and second with wPLI data. We started by calculating the average θ activity (between 4 and 7 Hz) for correct wait and incorrect wait trials across each session, and performing a logistic regression with θ activity as the dependent variable and trial type (successful or failed waiting) as the independent variable. We chose a select 11 brain regions for this analysis that showed the strongest θ activity/wPLI connectivity. We found only a few relatively weak differences between successful and unsuccessful inhibition trials related to θ power (Fig. 8*A*). We next performed a logistic regression using pair-wise θ wPLI (averaging activity from 500 to 900 ms poststimulus) from successful versus unsuccessful wait trials as our dependent measure and outcome as the independent measure. We found that pair-wise connectivity strongly differentiated successful versus unsuccessful inhibitory trials (Fig. 8*B*). Connectivity between M1 and vOFC was the strongest predictor of successful inhibition, with an overall *R*^2^ value of 0.53, adj. *p* < 1.8e-06. Because connectivity between these two regions showed the strongest pair-wise correlation with behavior (and prior studies have shown behavioral inhibition is related to connectivity with motor brain regions; Swann et al., 2012), we performed a follow-up multivariate model focusing on M1. For this model, we used connectivity (wPLI) values from M1 to the 10 other regions calculated within the 500- to 900-ms time period after trial onset. The M1 model had an overall *R*^2^ value of 0.784, *p* = 2.45e-23, with six significant predictors (Fig. 8*C*). There were three significant positive predictors of inhibition (i.e., regions in which greater connectivity was associated with improved inhibition): vOFC (β = 32.6, *p* = 0.0007), NA core (β = 30, *p* = 0.009), and dorsomedial striatum (β = 27.8, *p* < 0.05). Interestingly, there were also three regions in which connectivity had a negative β value (i.e., connectivity was associated with greater likelihood of responding impulsively): M2 (β = −20.7, *p* = 0.014), ALM (β = −17.7, *p* = 0.004), and anterior insula (β = −21.9, *p* = 0.04). Thus, we showed here that functional connectivity with M1 strongly differentiated correct (i.e., patient) versus incorrect (i.e., impulsive) activity for wait trials. Connectivity between M1 with ventral striatum and OFC was associated with improved inhibition while connectivity with other motor brain regions was associated with earlier/unsuccessful actions.

To complement the logistic regression model, we used an SVM model to assess how well we could classify trial-type across time simply with M1 connectivity (Fig. 8*D*). We also performed the analysis at both θ and β frequencies as a way to control for the specificity of our results for θ frequencies. We found that an SVM using θ frequencies could classify successful and unsuccessful wait sessions with an accuracy >80% starting at 600 ms after the stimulus and connectivity continued to predict successful inhibition above 80% for the entire waiting period after that. The model using β-frequencies never performed above 80%. Consistent with the logistic regression model, we found that wPLI from vOFC, medial striatum (including dorsal and ventral striatum and nucleus accumbens core) was useful for classification. Thus, using multiple analytic methods we show here that successful inhibition is associated with greater connectivity between M1 with ventral striatum and ventral orbitofrontal cortex.

### δ And θ oscillations measurable in human EEG on a parallel go/wait task

**Figure 9.**
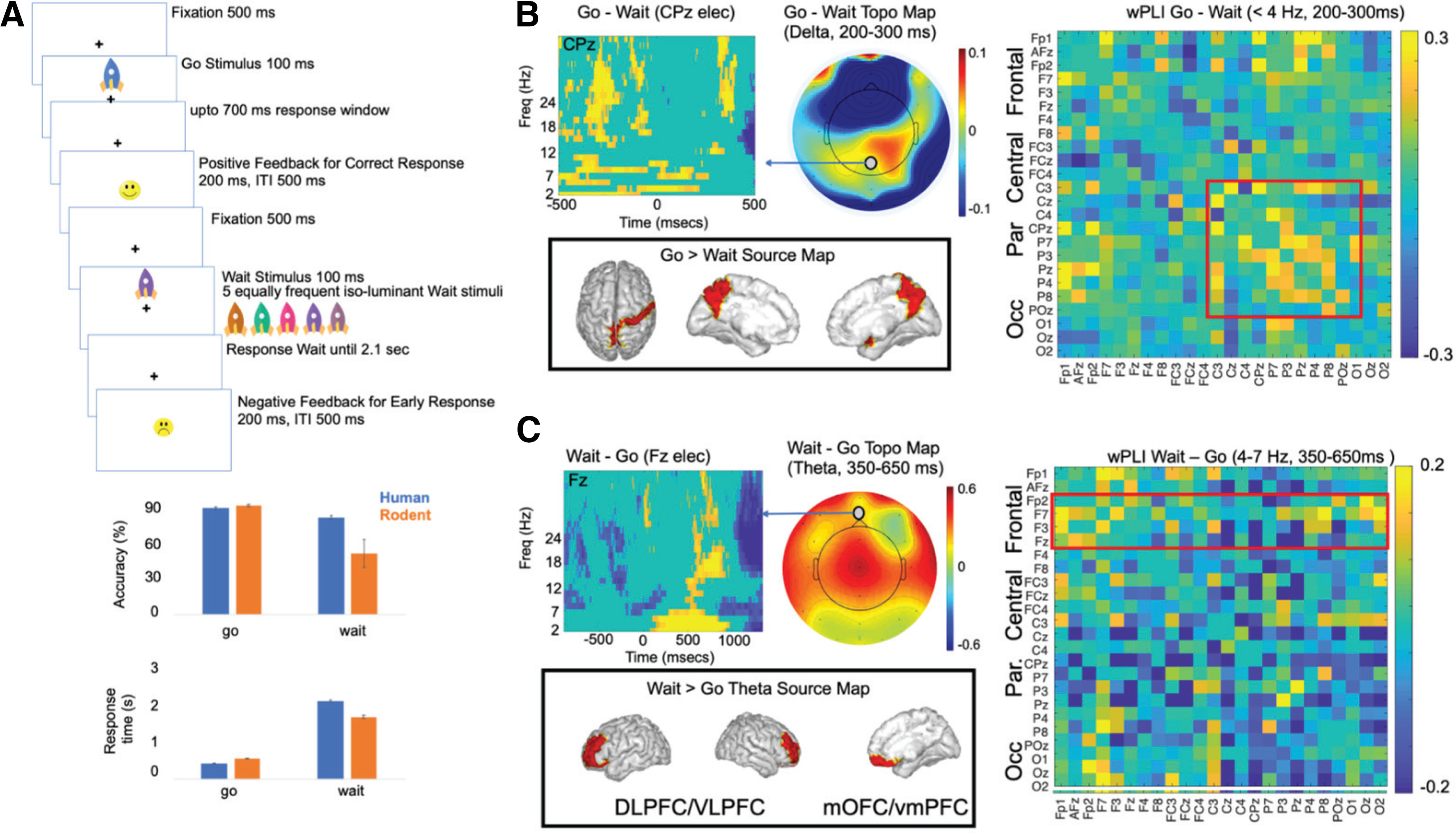
Go wait task in humans. ***A***, The human task was designed based on the rodent task, although with a more complicated set of stimuli. Go trials were indicated by a blue rocket, while any other color indicated a wait stimulus. Humans performed similarly to rats on go trials but were much better on wait trials. ***B***, Action-related activity (contrast of [go–wait]) could be observed at δ frequencies in centro-parietal electrodes (average 200–300 ms poststimulation). The outline of the head in the scalp topography maps is not to scale and only shows head orientation. This activity source localizes to precuneus and postcentral gyrus. wPLI showed significant effects for this contrast primarily between centro-parietal electrode locations (*p* < 0.05). ***C***, Activity linked with inhibition [wait–go] could be seen in frontal electrodes, particularly in θ frequencies (average 350–650 ms poststimulation). θ Activity source-localized to ventral and OFC areas, similar to that observed in rodents. Significant wPLI activity at θ frequencies could be observed between frontal electrode sites (*p* < 0.05).

We used a similar task design to probe whether δ and θ oscillations occurring during the task were observable in human EEG associated with sensorimotor responses and behavioral inhibition. In the human version of the task (Fig. 9*A*) subjects were exposed to a range of colored stimuli, one of which (“green rocket”) was designated as the go cue and the rest which were designated as the wait cue. On the go cue subjects had to respond quickly (within 700 ms) to get rewarded; on wait trials subjects had to wait 2100 ms before responding. Thus, on both human and animal versions of the task the basic rules were similar, although the stimuli were different. Importantly, on both animal and human versions of the task, the wait stimulus remained on during the waiting period, with the “offset” of the cue an indication that subjects should respond. On go cues human subjects performed similarly to animals in both RT and accuracy; human subjects performed far better at waiting compared with rodents (Fig. 9*A*). We analyzed the human data with a focus on identifying similar patterns of activity. Thus, we first analyzed δ activity associated with the go cue (i.e., we performed a [go–wait] contrast). We found (Fig. 9*B*), that cued-actions were associated with early, preresponse δ activity over centro-parietal electrodes. Significant pair-wise wPLI was observable between these centro-parietal electrodes at δ frequencies as well. This δ activity source-localized to postcentral gyrus and precuneus locations which overlaps with what we observed in rodents. Surprisingly (and distinct from rodents) this cued δ activity did not directly localize to M1. We next assessed whether there was greater θ activity during wait compared with go trials. We found that waiting was associated with significant mid-frontal θ activity (Fig. 9*C*). We found significantly greater wPLI in θ frequencies for wait compare to go trials predominately within frontal electrodes. This θ activity was source localized to lateral PFC (both dorsal and ventral) and medial OFC, at least partially overlapping with what we observed in rodents.

## Discussion

In this study we implanted electrodes across multiple brain areas and recorded LFP activity as animals performed a visual-based inhibition task, adapting an approach previously used to characterize slower changes in mood/affective states (Hultman et al., 2018). We identified two distinct brain networks associated with action and a distributed brain network associated with behavioral inhibition. Specific patterns of connectivity were associated with successful inhibition on this task. This study highlights several features of LFPs that make it useful for capturing activity in mesoscopic circuits related to behavior. As has been noted before, LFP can capture rapid temporal dynamics similar to EEG/magnetoencephalogram/single units, allowing for comparison across multiple scales and even species (Gulati et al., 2014; Ramanathan et al., 2018). LFP probes are highly customizable and fabricated easily to measure activity from many target regions simultaneously. The resolution of LFP data does not compare with what can be achieved with advanced single-unit probes (Juavinett et al., 2019) or advanced 2-P mesoscopic imaging (Lake et al., 2020). However, those approaches also typically require expensive equipment and training to use adequately and are difficult to perform in a high-throughput way. By contrast, measuring LFPs is easily compatible with many commonly used operant or other behavioral systems and possible for a wide variety of labs. One final advantage of the approach here is the ability to probe specific hypotheses in a few specific brain regions while also being able to discover patterns of activity/connectivity linked with behavior that would otherwise not have been found. This approach allows for the discovery of putative brain networks that can then be interrogated using more time-intensive single-unit/optogenetic approaches. A final and key advantage of LFP is general stability of signal across days. As we have shown previously, this allows us to perform such distributed measurements across days, providing the ability to track learning, short-term and long-term effects of injury, and effects of interventions (Ramanathan et al., 2018; Lemke et al., 2019).

### Distinct motor systems involved in action and sensory response mapping

We and others have previously shown that low-frequency oscillations are involved in skilled motor actions (Totah et al., 2013; Ramanathan et al., 2018; Gruber et al., 2009; Lemke et al., 2019). Similar low frequency oscillations associated with motor actions have also been observed in single unit data in primates (Churchland et al., 2012), as well as with electrocorticography and EEG recordings in humans and track recovery after stroke injury (Ramanathan et al., 2018; Bönstrup et al., 2019). These findings recapitulate that prior work. The role of these lower-frequency oscillations in mediating longer-range coordination of motor systems is still being established (Lemke et al., 2019). Here, we have identified several distinct aspects of how these low-frequency oscillations coordinate distributed neural circuits involved in motor decisions in rodents. First, we show that cued-actions are associated with a distributed low-frequency network that includes visual, parietal, reward, hippocampal circuits along with ventral portions of M2 (Erlich et al., 2011; Barthas and Kwan 2017).. This distributed circuit likely plays a key role in sensory-response mapping (Erlich et al., 2011; Hill et al., 2011; Kopec et al., 2015; Duan et al., 2015; Barthas and Kwan, 2017), which would presumably require vision, reward-anticipation and memory. This sensory-response mapping network is connected to a distinct motor subnetwork (comprising anterior and posterior portions of M1) at least in part through parietal cortex. We found similar low-frequency oscillations in precuneus (a visual-attention brain region) and postcentral gyrus (sensorimotor region) in humans in related to cued-actions. These results help to elaborate on prior theories regarding the FOF (Vogt and Paxinos, 2014; Fillinger et al., 2017), and show, in a new way, how this brain region is situated in a larger network mediating sensory-response mapping functions. Anterior secondary motor target (here labeled as M2), by contrast, showed involvement primarily with motor inhibition and not cued action which is also consistent with prior work suggesting involvement in this region in timing and impulsivity (Narayanan and Laubach, 2006; Narayanan et al., 2013; Narayanan, 2016).

### θ Activity in ventral striatum/OFC involved in behavioral inhibition

Impulsivity (the opposite of behavioral inhibition) has been characterized using a number of behavioral tasks (Dalley et al., 2011; Dalley and Robbins, 2017), including action-postponement (waiting; Navarra et al., 2008; Narayanan et al., 2013; Bari et al., 2008; Hardung et al., 2017), stop signal reaction time tasks (SSRT; Eagle et al., 2009; Bari and Robbins, 2013), and standard go/no-go tasks (Masaki et al., 2006). Impulsivity can also be measured as the ability to choose a delayed larger reward rather than a smaller immediate reward (Rokosik and Napier, 2012; Jo et al., 2013). Ventral striatum and OFC have been consistently implicated in many of these various forms of impulsivity (Basar et al., 2010; Burton et al., 2015; Dalley and Robbins, 2017). How and why it does so is less clear. Our study provides further evidence that vOFC and ventral striatum/nucleus accumbens form a functional inhibition network with M1 that is directly linked with successful inhibition during the waiting period. Interestingly, we also show here that the nucleus accumbens core is a key part of this inhibition network, while NAcS is more closely linked with action. This functional dissociation has been observed in other ways (Basar et al., 2010; Besson et al., 2010; Dalley and Robbins, 2017), but this is the first clear illustration of a network-level dissociation measurable using LFP between these two parts of accumbens.

As with all measurements of electrophysiology, our data are correlative and does not demonstrate a causal (or even specific) mechanism by which connectivity between M1 OFC/ventral striatum contributes to successful inhibition. Additional experiments, involving single-unit measurements, optogenetics and behavioral pharmacology, will be required to determine a more mechanistic framework for how these brain circuits mediate an improved ability to wait. However, our findings establish a clear platform in which to explore specific hypotheses and questions. For example, one area of interest is in exploring whether pharmacologic treatments that improve inhibition and diminish impulsivity do so via modulation of θ oscillations or functional connectivity. Prior work has identified a 4-Hz oscillation in medial frontal cortex, observed in both humans and animals, during a waiting/timing task (Narayanan et al., 2013; Emmons et al., 2016, 2017). This oscillation, demonstrated at the level of single units as well as LFP/EEG, seems to be modulated by prefrontal D1 receptors (Emmons et al., 2017), and is attenuated in the absence of dopamine in animals and humans (Parker et al., 2015). These studies suggest dopamine may be involved in our circuit as well. Additionally, however, there are a number of studies suggesting that serotonin can improve waiting in part by modulating OFC (Eagle et al., 2009; Miyazaki et al., 2012, 2014; Worbe et al., 2014; Lottem et al., 2018) and, thus, is likewise a worthy area of investigation.

### Comparison with human EEG data

One of the theoretical advantages of LFP (compared with single-unit investigations or calcium imaging, for example), is the closer parallel to oscillatory activity measured with EEG. Here, by running a similar action postponement task in humans, we identified δ activity linked with sensory-cued motor responses (go trial contrast) and θ activity in OFC and ventral PFC associated with behavioral inhibition (wait trial contrast). It is important to note that this activity contrast is similar but not identical to that observed in rodents. In humans, the δ activity associated with action source-localized to parietal and visual cortical regions but not (unlike in rodents) directly to M1. The inhibition-related θ activity source localized to OFC and lateral PFC, but not to many of the other brain regions (including dorsomedial PFC) in which we observe such activity in rodents. There are a number of reasons why these differences in anatomic localization may occur. First is the fundamental issue that rodent and human PFC are very different (Laubach et al., 2018), and thus comparisons between the two are always going to challenging. Additional factors precluding easy comparison between species include differential sensitivity of scalp EEG and LFPs; inaccuracies of source-localization, differences in volume conduction between two recording types, etc. Moreover, even finding similar oscillatory activity on similar tasks localized to similar brain regions may not reflect the same neural circuits across species. Our findings of similar electrophysiological frequencies during the task associated with action and inhibition are interesting but must not be overinterpreted.

### Limitations and future studies

There are several limitations to this approach of characterizing brain activity. First, while we use standard stereotactic methods to implant electrode bundles, deeper sites are invariably less reliably targeted than superficial sites. We localized electrode bundles in a subset of animals (4), and found they are within a few mm of targeted sites. In general, less precision of anatomic location will diminish the precision by which we can localize signals to any particular brain area. This problem is further exacerbated by volume conduction of LFP more generally (Buzsáki et al., 2012; Pesaran et al., 2018). In addition to spatial imprecision, interpreting LFPs from different brain regions (particularly non-laminar ones) can be difficult. While certain brain regions have well-documented oscillations linked clearly with phase-related spiking activity (Buzsáki and Draguhn, 2004; DeCoteau et al., 2007; Sirota et al., 2008; Cardin et al., 2009; Sohal et al., 2009; Adhikari et al., 2010; Howells et al., 2012; Leventhal et al., 2012), in many non-laminar structures there is not a simple theoretical basis to interpret LFP signals (Buzsáki et al., 2012; Pesaran et al., 2018). In this study, we have focused on characterizing empirical observations regarding LFP, however, follow-up studies will be required to link identified oscillations with underlying spiking activity in relevant brain regions. Generalizing our findings will require examining activity in these brain circuits (using a similar whole-brain LFP approach) in a variety of other tasks in which we parametrically manipulate various aspects of attention, task-difficulty and reward/reward-feedback to better understand the specific relationships between activity and behavior, much as has been performed in conjunction with single unit studies.

In summary, despite some limitations of LFP recordings, here we show that LFP can be used to map multiregion brain activity in rodents performing a cognitive task. We have used this to define specific brain networks involved in action and inhibitory processes, that provides a clear basis for many further investigations into underlying mechanistic processes subserving this important behavior.

## Synthesis

Reviewing Editor: Mark Laubach, American University Decisions are customarily a result of the Reviewing Editor and the peer reviewers coming together and discussing their recommendations until a consensus is reached. When revisions are invited, a fact-based synthesis statement explaining their decision and outlining what is needed to prepare a revision will be listed below.

With regards to advances to the field, one reviewer wrote “This manuscript performs distributed LFP recordings during a task involving behavioral inhibition and considerably advances our knowledge of low-frequency dynamic.” The other wrote “The study advances the field by providing the large-scale LFP recordings from 31 regions simultaneously; however, the analyses chosen and they way they are reported seriously compromise the conclusions that can be drawn from the data set.” Based on the reviews and correspondence in the review forum, the consensus was that the study is very much worthy of publication. However, the reviewers felt the need for major revisions. The second reader said that this is the longest review that they have ever submitted, and hope that the comments are useful for improving the manuscript.

Please be sure to address each point in your rebuttal letter and “redlined” revised manuscript. We look forward to your revised manuscript.

Reviewer 1

I found this to be a technical and conceptual tour-de-force establishing the brain-wide dynamics of low-frequency activity during inhibitory control. The experiments and analysis are well-done.

I had some minor questions that might help readers understand the work better:

1) Would it be clearer to put Figure 9 as Figure 1? While reading the manuscript and figures, I wondered if the data came from rodents or humans, but only the current Figure 8 is humans, and all other figures are rodent. I also wondered quite a bit about the anatomy while reading the paper, so having the anatomy up front may have helped readers interpret these data. The text and figures could better indicate where the data came from and in how many animals and electrodes to get a sense of effect size.

2) Similarly, with so many LFP sites, I wonder if a 3D model or a space-filling reconstruction would help readers understand the scope of the recordings.

3) Figure 1B - are these the actual stimuli used? Making things as close as possible to the rodent task would be critical - and the human image in Fig 1B confused me (was this humans or rodents?). Are Fig 1A and B referred to in the text?

4) Should Fig 1E be much bigger - with separate colors from other panels?

5) A33 is featured prominently - but this terminology is not very common - some clarification in the text and some anatomy might help.

6) Would readers understand Fig 7? Would they be convinced that it wasn’t overfitted (was there shuffled data? Is there 100% classification in 7C?) Did logistic regression consider all possible models? What criteria for model selection wereWith regards to advances to the field, one reviewer wrote “This manuscript performs distributed LFP recordings during a task involving behavioral inhibition and considerably advances our knowledge of low-frequency dynamic.” The other wrote “The study advances the field by providing the large-scale LFP recordings from 31 regions simultaneously; however, the analyses chosen and they way they are reported seriously compromise the conclusions that can be drawn from the data set.” Based on the reviews and correspondence in the review forum, the consensus was that the study is very much worthy of publicaton. However, the reviewers felt the need for major revisions. The second reader said that this is the longest review that they have ever submitted, and hope that the comments are useful for improving the manuscript.

Please be sure to address each point in your rebuttal letter and “redlined” revised manuscript. We look forward to your revised manuscript.

Reviewer 1

I found this to be a technical and conceptual tour-de-force establishing the brain-wide dynamics of low-frequency activity during inhibitory control. The experiments and analysis are well-done.

I had some minor questions that might help readers understand the work better:

1) Would it be clearer to put Figure 9 as Figure 1? While reading the manuscript and figures, I wondered if the data came from rodents or humans, but only the current Figure 8 is humans, and all other figures are rodent. I also wondered quite a bit about the anatomy while reading the paper, so having the anatomy up front may have helped readers interpret these data. The text and figures could better indicate where the data came from and in how many animals and electrodes to get a sense of effect size.

2) Similarly, with so many LFP sites, I wonder if a 3D model or a space-filling reconstruction would help readers understand the scope of the recordings.

3) Figure 1B - are these the actual stimuli used? Making things as close as possible to the rodent task would be critical - and the human image in Fig 1B confused me (was this humans or rodents?). Are Fig 1A and B referred to in the text?

4) Should Fig 1E be much bigger - with separate colors from other panels?

5) A33 is featured prominently - but this terminology is not very common - some clarification in the text and some anatomy might help.

6) Would readers understand Fig 7? Would they be convinced that it wasn’t overfitted (was there shuffled data? Is there 100% classification in 7C?) Did logistic regression consider all possible models? What criteria for model selection were used? I might suggest that this adds little to the manuscript - as theta appears to be distributed across the network, and pairwise analyses do not capture these distributed dynamics. I might suggest removing this figure.

7) Fig 1C - did all rodents in this figure have physiological data?

8) These low-frequency rhythms depend on dopamine in humans and rodents: PMID 26133799 & 27989675 and are reliably disrupted in Parkinson’s disease: PMID: 29876277- I wonder if these studies would further add to this study. These rhythms are also disrupted in schizophrenia: PMID: 11009074

9) Some might expect Fig 8C to be more prominent and include midfrontal rather than motor electrodes. A plot behavior in Fig 8B might help readers understand the main effects.

10) There are very few y-axis labels? A list:

Fig 1D - y-axis labels

Fig 2 - all need y-axis labels, 2D needs a z-label

Fig 3: all need y-axis labels

Fig 4: all need y-axis labels, and A needs a z-label

Fig 6: needs both y and z labels

Minor text:

Intro: ramping activity has been shown to be a common theme in cortical activity well beyond the Mainen paper

Methods - page 5 - I found coordinates hard to follow - replace with a table?

What were the parameters for newtimef?

Before 12ua needs a space

The paragraph before Neural analysis needs a space

Results - I wonder if the long stats blocks i.e. after Fig 2E would go better in a table?

Reviewer 2

The authors recorded simultaneously from 31 brain regions during a “waiting” task. Some of these regions have been implicated in impulsivity and action control (i.e., moving or stopping/waiting/inhibition) by prior studies that recorded EEG, LFP, or single unit spiking. The main advance of the present study is to record from these previously-implicated brain regions, as well as a broader set of regions, simultaneously. The authors recorded LFP instead of single unit spiking activity and could therefore also study the characteristics of the frequency spectrum in these regions which are related to action control. Naturally, LFP offered the advance over prior EEG work of being more localized and targeted to sub-cortical regions. Finally, the authors compare their results to the results from human EEG. In order to study these data, the authors examined both spectral power and pairwise (region-pair, not electrode-pair) phase locking using a weighed phase synchrony index (wPSI) from Vinck, et al. 2011 (doi: 10.1016/j.neuroimage.2011.01.055.). The authors conclude that different “networks” of regions are involved in carrying out an action versus inhibition an action (waiting), that the networks are related to cross-region phase-locking in different frequency bands (delta - action, theta - inhibition), and that these networks are anatomically similar in rats and humans.

Novelty: the data set (31 brain regions) is novel and is a laudable effort to collect such large-scale cortical and sub-cortical LFP data during a decision-making task. Prior work on EEG and LFP has already shown that theta oscillations are involved in action inhibition (e.g., studies cited by the author and also studies on stop signal tasks and conflict tasks, and Vinck et al 2011 NeuroImage) and that delta oscillations are involved in actions (e.g., studies cited by the author, and also Gruber et al 2009 PlosOne, Totah et al 2013 J Neurosci). Given these prior findings focused on a singular brain region or two brain regions recorded simultaneously, the main strength of the present large-scale multi-region dataset is to look at cross-region interactions. There are numerous methods to examine the authors’ data, such as graph theoretic analyses and frequency-resolved causal measures such as Granger causality and information transfer. The authors chose to examine network activity using one narrow analysis: changes in wPSI between region-pairs. With the large data-set of 31 regions, it would be beneficial to see further characterization using graph theory and/or granger causality, if not in the present study then in future work.

I have a number of major concerns. The broadly include:

(1) how the data are presented and communicated to the reader

(2) how statistics and analysis are explained

(3) how the data are analyzed and used to support the authors’ conclusions

(4) poor grammar and unprofessional writing style.

The data set is an excellent one and I hope that my comments will help the authors to communicate findings based on this data set more clearly in order to advance the field.

Below, I give a point-by-point list of major and minor concerns with accompanying discussion of these points and I offer some helpful suggestions to address these concerns. The concerns are not listed in any particular order or priority.

1. Page 5, first sentence - says “25% wait/75% go trials” was the next stage. And the first stage (training) on page 4 says “a distribution of 75% go and 25% wait”. These appear to be identical, so what exactly changed between the training stage and testing stage?

2. Please state in the methods the number of trials per session, which I could not find in the manuscript. The reason this is important is that the data were averaged across-trials within each session and those session-averaged were used as the individual data points in the study. The reader needs to be able to judge if a sufficient number of trials were used in each session to obtain this average.

3. Surgery sub-section of methods - how often were drugs administered? All a single dose? What was dose of lidocaine? What was dose of buprenorphine SR? What was the metal and insulation for the electrode? What was electrode wire size? What were the impedances of the electrodes (e.g., a range)?

4. 2nd paragraph of the Surgery sub-section of the methods - This long list of cannulae and regions in the text is very dense and it makes it difficult to obtain information and assess implant location. I have a suggestion. The authors could make a figure showing various coronal sections with the implanted regions shaded in different colors. A legend could show region name and the AP, ML, DV coordinate. “AP", “ML", and “DV” could be written once at the top of the legend, such that the coordinates did not have to be repeated each time. This would greatly help the reader assess the regions implanted, which is critical given that the large-scale recordings from 31 regions is the major advance in this study.

5. Methods - an electrophysiology section is missing. It needs to explain the sampling rate and all filters used on the LFP signal.

6. Methods, analysis sub-section - “Mean TF data from each session was z-scored prior to performing average or statistical tests to account for....” First, there is a typo: “averaging” should be written. Second, it is not clear what was used to calculate z-score. A mean spectrogram (“TF data” is the name used by the authors) aligned to an event (e.g., stimulus onset) was calculated for each event and then averaged across the events (trials) in a session. This mean spectrogram was now z-scored to the mean and standard deviation or something. What was used to calculate mean and SD? In general, the paper would benefit from a clearer explanation of these steps of analysis and statistics - perhaps a graphical example showing each step would be beneficial because a lack of clarity in how analyses and statistics were carried out comes up repeatedly in the manuscript.

7. Methods, analysis sub-section - “we performed a one-sample, two-sided t-test at each time-frequency point for each electrode, separately for each trial type (go correct, wait correct, wait incorrect).” Note that no comma is needed before separately. What mean was being tested against? It should be clarified: were these spectrogram values already trial-averaged and then z-scored? So each point in the t-test was a session (multiple sessions per rat)? From that, it seems that the authors were likely testing for an increased or decreased z-score in comparison to no change (mean Z=0). Is this the case? It should be explained clearly in the methods in order to understand the results.

8. Methods, analysis sub-section - “delta (1-4Hz(); theta (4-7Hz)....” Typo after 1-4Hz and also commas instead of semi-colons because it is a list of items.

9. Methods, statistical analyses sub-section - ML and SVM should be spelled out the first time they are used so that readers unfamiliar with the topic can understand the methods used in the paper

10. Methods, statistical analyses sub-section - “Data was mostly confirmed and significant at the level of animals a well (showed in Data Tables)....” Typographical mistakes: “as well” and “as shown in Data Tables"

11. Methods, sub-section EEG - “...device with a semi-dry and wireless electrode layout in .” Typo / incomplete sentence.

12. Methods, neural analysis sub-section - was theta 3-7 Hz or 4 - 7Hz as stated later?

13. What type of wavelet was used and what were the parameters?

14. Results - response times, Fig 1 and associated main text - There must be a lot of variability across response times (RT) on wait trials within a session given that 50% of the wait trials were correct (meaning the rat waited at least 2000 msec) and 50% of the wait trials were incorrect and likely responded closer to the mean of the Go stimulus RT (500 msec). Figure 1C and the mean/SEM’s reported in the text would be improved by presenting trial-wise data from all sessions and rats, rather that session averaging which removes all of this variability. The authors could also separate RTs on Fig1C according to whether it was correct wait or incorrect wait trial.

15. What is the definition of RT? Is it the time from stimulus onset to target poke? Or stimulus onset to withdrawing middle poke? Or time from withdrawal of middle poke to engaging target poke? This should be stated clearly somewhere in the manuscript. Stating in results section would help clarify rational for time windows for electrophysiology data analysis.

16. In all Figures until Figure 7, the contrast between Go and Wait included correct and incorrect wait trials? Or only correct wait trials? If the authors wish to highlight differences in brain activity between moving (Go) and inhibition (Wait), then it would be best to exclude incorrect wait trials from this contrast. The time window chosen for the analysis appears to be correct for examination of this behavioral contrast.

17. Page 10, Fig 2B and 2C have “coordinates: , p<0.05”. Coordinates are not listed, nor are they listed in this format later in the paper. This appears to be a typo.

18. Fig 2 - The authors state that delta activity was strongest in sensorimotor regions. In Figure 2D it looks to be wide-spread and similar strength in other regions compared to sensorimotor ones. Fig. 2D does not provide a convincing evidence for the authors statement that it was stronger in sensorimotor. In Figure 2E, the values across regions also look highly similar. The p values reported in the text related to Fig 2E are I assume, one-way t-tests against the mean of 0. This test type needs to be reported where the p-value is reported so that it can be understood what these p-values are related to and the mean/SEMs in the results text could be removed for readability because one can see those values in the Fig 2E.) Most critically, the statistical test does not support the authors’ statement that delta activity was strongest in sensorimotor regions. For that, the authors should do a one-way ANOVA (or non-parametric test depending on how their data are distributed) with region as a factor and then post-hoc tests to compare specific regions to assess strength differences. For any post-hoc tests that are significant, the authors should report the power of the test and they should report an effect size between the two groups.

19. Fig 3 / wPLI results text - the authors write from one region “to” another. However, wPLI has no directionality. It only shows phase locking “of” or “between” two electrodes, not a putative causal influence of one signal to another. (The authors can add transfer entropy, Granger causality, or use values from wPLI to created a directed graph and analyze it for clustering to make further use of their unique data set and support claims about directional network interactions).

20. Fig 3A - It is not explained what the titles are in the plots in comparison to the x-axis. For example, is the plot labeled with the title “M1” an analysis which used M1 signal as signal A and ALM on the x-axis is signal B? Given that wPLI is not directed, there should be no difference between M1 (signal A) and ALM (signal B) versus ALM (signal A) and M1 (Signal B). Moreover, if there is phase synchrony between A24b and M1, as well as A24b and ALM, the ALM and M1 should have phase synchrony. Can the authors explain this? ALM and M1 are nearby and my have close to zero-lag phase locking which will not increase wPLI (see Vinck et al 2011). Therefore, wPLI may not be appropriate to use as the one single measure of network activity. Using additional measures (granger, transfer entropy) and looking for convergent findings across multiple methods would yield a more evidence and enable the authors to make more robust conclusions based on their data.

21. Page 11 - “global” connectivity. The authors need to clarify what this measure was and how it was calculated. It could not be understood from the manuscript in its current form.

22. Figure 6C - comparing strength across regions should use appropriate statistical test and the text can be modified where describing the results (see item 18 above).

23. Page 12 - the authors report no difference in power from 500-900ms post stimulus and then look at wPLI. However, for wPLI, they use a different window of 500-900ms post response. Why does the time window of interest change?

24. Page 12 - the authors report no difference in power from 500-900ms post stimulus. It is important to show the data instead of only stating the finding without reporting any numerical results in a text or figure that support this statement.

25. Page 12, logistic regression - the authors mention a few times “simple logistic regression”. Is there something different than a logistic regression?

26. Page 12 - the authors state that they “performed a simple logistic regression between connectivity and response time for each pair-wise interaction.” The exact procedure needs to be explained because, as written, the analysis is not clear. For example, was the regression looking at the relationship between the wPLI value for a pair of regions and the response time (a numeric value) or the trial type (a binary value)? Was the wPLI values first averaged across electrodes/sessions/rats within a brain region? What data points went into this logistic regression?

27. Results sub-section on “translational relevance” - The authors cannot translate directly to human “networks”. The brain regions are too different. This is not to say that some anatomy does not overlap or that there is no translational value in the data. However, I think translation could be made on the basis of finding that there are two networks (one with go-specific activity and one with wait-specific activity) in both species and/or that the relevant spectral frequencies overlap between species (go-delta, wait-theta). However, I do not think that the translation should be made on the basis of a few regions overlapping partly because the anatomy is too different and partly because the methods are different (human cortical EEG vs rat cortical and subcortical LFP).

28. However, if the authors were to make a translational link based on number of networks or frequency content (as explained above in comment #27), there is the larger issue that Figure 8 provides little evidence to support either claim. If the authors want to support this claim, it will be necessary to take the human data through the same steps as the rat data. What did response times look like in the human task and how does that justify time windows for EEG analysis? Analyze the power and WPLI in the same was in humans and rats. Show the same color plots from Figure 2A, B, C, D and Figure 5A, B, C for human data analyzed in the same way. The data need to be studied and presented in the same way in order to make a translational link.

29. How were the values plotted in Figure 2 (and later figures) for power? Is the color bar a z-score that has been session and rat averaged? As mentioned earlier, it would be helpful to have a clear explanation for how data were analyzed in the results. I understand from reading entire manuscript that the following steps occurred: (1) subtract baseline activity (defined as mean over time before stimulus) on each trial, (2) average across events within electrode (one electrode per region), (3) z-score (as mentioned above, unclear how z-score was calculated), (4) t-test each time-frequency point using a one-sided test against some mean (as mentioned above, it is not clear what this mean for the one-sided test was) with each data point being a session, (5) set all points in the spectrogram with non-significant values to a Z-score of 0 (what was the p-value threshold for this thresholding?), (6) average the data across sessions, (7) plot the data for example Figure 2A. Is this accurate? A graphical flow chart or sentence stating this succinctly in the results would greatly aid in the readability of the manuscript.

30. What is further unclear is how the go-wait contrast was calculated and how the “conjunction analysis” was done. How is a significant increase in Go determined? If significance was determined using one-sided t-tests for each time-frequency bin, then how does this translate into significant increase in Go trials? Was this done on each time-frequency bin or in bands?

31. How was the go-wait contrast calculated? Was the Z-scored spectral power in wait subtracted from the Z-scored spectral power in Go trials? Was this done on trial-averaged data? And then significance was determined for each time-frequency pair by one-sided t-test?

32. How was the conjunction of these two tests performed? A significance value for both Go and Go-wait? Was this conjunction used to set Z-score values to 0 in the plots in Figure 2A, B, C, D and similar plots in Figure 5?

33. I strongly disagree with changing values to 0 based on a statistical test. There are numerous reasons that a p-value could be above or below an arbitrary threshold that are unrelated to the question being studied. To then use that arbitrary threshold to zero-out data for plotting seems like an unnecessary step that radically affects the data. Can the authors just plot z-scores without this mask and change of values? Bands of interest in the spectrogram could still be visualized and then, for the purpose of statistical testing, the authors could get band-limited means like the line plots in Fig 2A, B, and C right panel and do ANOVA (time repeated measure) on those followed by post-hoc tests. An alternative would be to make a separate plot in which non-significant values are changed to NaN before getting mean and SEM. However, I think it is critical to show data without changing the values to NaN or 0, too. The same goes for wPLI analyses: I do not think it is apporpiate to change data to 0 and then plot it as the result.

34. Figure 2 - legend has a typo: “(significant differences between the two denoted with *. Using FDR-correction across....).” Also, I think that throughout the text, reading FDR-correction over and over is distracting and that stating it once in the methods is enough.

35. Figure 3 - title says 0-400msec but main text suggests 300-500msec

36. Figure 5 - there is in both A and B an increase in beta and gamma that seems to be higher in wait correct than in go. Can the authors examine this further?

37. Figure 5D - is the y-axis Z-scores? What are the *’s? A significant one-sided t-test against a mean of z-score = 0? A Z-score >2 is taken as 2 s.d. from the mean. A “significant” deviation in z-score from 0 with an effect size of only 0.6 seems to be an extremely small effect and the various significant findings may only be due to variability and high sample size (many sessions), whereas the actual change in the data is extremely small. Can the authors discuss this?

38. Figure 8C and D - what is -ve delta? This is not explained in the legend or results text. As mentioned above, it is critical to have Figure 8 follow same format as the rest of the manuscript and analyses of rat data to compare species.

39. Were any source localization (e.g., beam forming, etc.) measures performed on the human EEG to make it more localized to a region?

40. Discussion section, 1st paragraph - a novel way for characterizing rapid temporal dynamics was not established in this work. These methods are all previously established. The data set itself (31 regions from rat) is novel and the effort to obtain it is commendable.

41. “adapting an approach previously to characterize slower...” - I think the word “used” is missing. There are also instances of commas mis-used and was/were changes throughout this section

42. “specific patterns of connectivity was [were] associated with overall levels of behavioral inhibition” - I think overall level was not measured (e.g., relation between connectivity and how many trials, overall, were correctly inhibited)

43. “local-field-potentials” has dashes in discussion section and it did not before

44. “scalable, data-driven solution” - it was not clear what this phrase means

45. “more comprehensive approach” - more comprehensive in comparison to what?

46. “reversal learning paradigms, measuring the tendency” - “,which measure the tendency...” would be better. The rather long sentence has to be read a few times to understand it. And the next sentence uses commas and then switches to semi-colons part way through the sentence (in this case, commas are grammatically correct).

47. “It is well know that OFC contains a large diversity of neurons...” - Can phase relations with another brain region LFP or EEG somehow index different cell types and the relationship between their activity and different aspects of cognition?

48. “Here, by running the same task in humans...” - The task was not identical

49. “interpreting the LFPs without spiking activity is always tricky”. This statement reads as opinionated and non-scientific

50. “LFP can be used to map whole brain circuit physiology....” - I think it is nicely large-scale or multi-region but it is not whole brain like fMRI for example. Also, I think brain activity - behavior relationship was studied but not “circuit physiology”. The task was not particularly “complex” cognitive task.

51. Figure 1A has does not add anything to the paper. It would be useful to see the layout of the screen and nose poke holes, but the 3-d rendering occludes that useful information.

52. Raw data should be shown (some LFP traces during the task showing raw LFP and then overlaid filtered delta or theta during the various trial types and during windows of interest) used? I might suggest that this adds little to the manuscript - as theta appears to be distributed across the network, and pairwise analyses do not capture these distributed dynamics. I might suggest removing this figure.

7) Fig 1C - did all rodents in this figure have physiological data?

8) These low-frequency rhythms depend on dopamine in humans and rodents: PMID 26133799 & 27989675 and are reliably disrupted in Parkinson’s disease: PMID: 29876277- I wonder if these studies would further add to this study. These rhythms are also disrupted in schizophrenia: PMID: 11009074

9) Some might expect Fig 8C to be more prominent and include midfrontal rather than motor electrodes. A plot behavior in Fig 8B might help readers understand the main effects.

10) There are very few y-axis labels? A list:

Fig 1D - y-axis labels

Fig 2 - all need y-axis labels, 2D needs a z-label

Fig 3: all need y-axis labels

Fig 4: all need y-axis labels, and A needs a z-label

Fig 6: needs both y and z labels

Minor text:

Intro: ramping activity has been shown to be a common theme in cortical activity well beyond the Mainen paper

Methods - page 5 - I found coordinates hard to follow - replace with a table?

What were the parameters for newtimef?

Before 12ua needs a space

The paragraph before Neural analysis needs a space

Results - I wonder if the long stats blocks i.e. after Fig 2E would go better in a table?

Reviewer 2

The authors recorded simultaneously from 31 brain regions during a “waiting” task. Some of these regions have been implicated in impulsivity and action control (i.e., moving or stopping/waiting/inhibition) by prior studies that recorded EEG, LFP, or single unit spiking. The main advance of the present study is to record from these previously-implicated brain regions, as well as a broader set of regions, simultaneously. The authors recorded LFP instead of single unit spiking activity and could therefore also study the characteristics of the frequency spectrum in these regions which are related to action control. Naturally, LFP offered the advance over prior EEG work of being more localized and targeted to sub-cortical regions. Finally, the authors compare their results to the results from human EEG. In order to study these data, the authors examined both spectral power and pairwise (region-pair, not electrode-pair) phase locking using a weighed phase synchrony index (wPSI) from Vinck, et al. 2011 (doi: 10.1016/j.neuroimage.2011.01.055.). The authors conclude that different “networks” of regions are involved in carrying out an action versus inhibition an action (waiting), that the networks are related to cross-region phase-locking in different frequency bands (delta - action, theta - inhibition), and that these networks are anatomically similar in rats and humans.

Novelty: the data set (31 brain regions) is novel and is a laudable effort to collect such large-scale cortical and sub-cortical LFP data during a decision-making task. Prior work on EEG and LFP has already shown that theta oscillations are involved in action inhibition (e.g., studies cited by the author and also studies on stop signal tasks and conflict tasks, and Vinck et al 2011 NeuroImage) and that delta oscillations are involved in actions (e.g., studies cited by the author, and also Gruber et al 2009 PlosOne, Totah et al 2013 J Neurosci). Given these prior findings focused on a singular brain region or two brain regions recorded simultaneously, the main strength of the present large-scale multi-region dataset is to look at cross-region interactions. There are numerous methods to examine the authors’ data, such as graph theoretic analyses and frequency-resolved causal measures such as Granger causality and information transfer. The authors chose to examine network activity using one narrow analysis: changes in wPSI between region-pairs. With the large data-set of 31 regions, it would be beneficial to see further characterization using graph theory and/or granger causality, if not in the present study then in future work.

I have a number of major concerns. The broadly include:

(1) how the data are presented and communicated to the reader

(2) how statistics and analysis are explained

(3) how the data are analyzed and used to support the authors’ conclusions

(4) poor grammar and unprofessional writing style.

The data set is an excellent one and I hope that my comments will help the authors to communicate findings based on this data set more clearly in order to advance the field.

Below, I give a point-by-point list of major and minor concerns with accompanying discussion of these points and I offer some helpful suggestions to address these concerns. The concerns are not listed in any particular order or priority.

1. Page 5, first sentence - says “25% wait/75% go trials” was the next stage. And the first stage (training) on page 4 says “a distribution of 75% go and 25% wait”. These appear to be identical, so what exactly changed between the training stage and testing stage?

2. Please state in the methods the number of trials per session, which I could not find in the manuscript. The reason this is important is that the data were averaged across-trials within each session and those session-averaged were used as the individual data points in the study. The reader needs to be able to judge if a sufficient number of trials were used in each session to obtain this average.

3. Surgery sub-section of methods - how often were drugs administered? All a single dose? What was dose of lidocaine? What was dose of buprenorphine SR? What was the metal and insulation for the electrode? What was electrode wire size? What were the impedances of the electrodes (e.g., a range)?

4. 2nd paragraph of the Surgery sub-section of the methods - This long list of cannulae and regions in the text is very dense and it makes it difficult to obtain information and assess implant location. I have a suggestion. The authors could make a figure showing various coronal sections with the implanted regions shaded in different colors. A legend could show region name and the AP, ML, DV coordinate. “AP", “ML", and “DV” could be written once at the top of the legend, such that the coordinates did not have to be repeated each time. This would greatly help the reader assess the regions implanted, which is critical given that the large-scale recordings from 31 regions is the major advance in this study.

5. Methods - an electrophysiology section is missing. It needs to explain the sampling rate and all filters used on the LFP signal.

6. Methods, analysis sub-section - “Mean TF data from each session was z-scored prior to performing average or statistical tests to account for....” First, there is a typo: “averaging” should be written. Second, it is not clear what was used to calculate z-score. A mean spectrogram (“TF data” is the name used by the authors) aligned to an event (e.g., stimulus onset) was calculated for each event and then averaged across the events (trials) in a session. This mean spectrogram was now z-scored to the mean and standard deviation or something. What was used to calculate mean and SD? In general, the paper would benefit from a clearer explanation of these steps of analysis and statistics - perhaps a graphical example showing each step would be beneficial because a lack of clarity in how analyses and statistics were carried out comes up repeatedly in the manuscript.

7. Methods, analysis sub-section - “we performed a one-sample, two-sided t-test at each time-frequency point for each electrode, separately for each trial type (go correct, wait correct, wait incorrect).” Note that no comma is needed before separately. What mean was being tested against? It should be clarified: were these spectrogram values already trial-averaged and then z-scored? So each point in the t-test was a session (multiple sessions per rat)? From that, it seems that the authors were likely testing for an increased or decreased z-score in comparison to no change (mean Z=0). Is this the case? It should be explained clearly in the methods in order to understand the results.

8. Methods, analysis sub-section - “delta (1-4Hz(); theta (4-7Hz)....” Typo after 1-4Hz and also commas instead of semi-colons because it is a list of items.

9. Methods, statistical analyses sub-section - ML and SVM should be spelled out the first time they are used so that readers unfamiliar with the topic can understand the methods used in the paper

10. Methods, statistical analyses sub-section - “Data was mostly confirmed and significant at the level of animals a well (showed in Data Tables)....” Typographical mistakes: “as well” and “as shown in Data Tables"

11. Methods, sub-section EEG - “...device with a semi-dry and wireless electrode layout in .” Typo / incomplete sentence.

12. Methods, neural analysis sub-section - was theta 3-7 Hz or 4 - 7Hz as stated later?

13. What type of wavelet was used and what were the parameters?

14. Results - response times, Fig 1 and associated main text - There must be a lot of variability across response times (RT) on wait trials within a session given that 50% of the wait trials were correct (meaning the rat waited at least 2000 msec) and 50% of the wait trials were incorrect and likely responded closer to the mean of the Go stimulus RT (500 msec). Figure 1C and the mean/SEM’s reported in the text would be improved by presenting trial-wise data from all sessions and rats, rather that session averaging which removes all of this variability. The authors could also separate RTs on Fig1C according to whether it was correct wait or incorrect wait trial.

15. What is the definition of RT? Is it the time from stimulus onset to target poke? Or stimulus onset to withdrawing middle poke? Or time from withdrawal of middle poke to engaging target poke? This should be stated clearly somewhere in the manuscript. Stating in results section would help clarify rational for time windows for electrophysiology data analysis.

16. In all Figures until Figure 7, the contrast between Go and Wait included correct and incorrect wait trials? Or only correct wait trials? If the authors wish to highlight differences in brain activity between moving (Go) and inhibition (Wait), then it would be best to exclude incorrect wait trials from this contrast. The time window chosen for the analysis appears to be correct for examination of this behavioral contrast.

17. Page 10, Fig 2B and 2C have “coordinates: , p<0.05”. Coordinates are not listed, nor are they listed in this format later in the paper. This appears to be a typo.

18. Fig 2 - The authors state that delta activity was strongest in sensorimotor regions. In Figure 2D it looks to be wide-spread and similar strength in other regions compared to sensorimotor ones. Fig. 2D does not provide a convincing evidence for the authors statement that it was stronger in sensorimotor. In Figure 2E, the values across regions also look highly similar. The p values reported in the text related to Fig 2E are I assume, one-way t-tests against the mean of 0. This test type needs to be reported where the p-value is reported so that it can be understood what these p-values are related to and the mean/SEMs in the results text could be removed for readability because one can see those values in the Fig 2E.) Most critically, the statistical test does not support the authors’ statement that delta activity was strongest in sensorimotor regions. For that, the authors should do a one-way ANOVA (or non-parametric test depending on how their data are distributed) with region as a factor and then post-hoc tests to compare specific regions to assess strength differences. For any post-hoc tests that are significant, the authors should report the power of the test and they should report an effect size between the two groups.

19. Fig 3 / wPLI results text - the authors write from one region “to” another. However, wPLI has no directionality. It only shows phase locking “of” or “between” two electrodes, not a putative causal influence of one signal to another. (The authors can add transfer entropy, Granger causality, or use values from wPLI to created a directed graph and analyze it for clustering to make further use of their unique data set and support claims about directional network interactions).

20. Fig 3A - It is not explained what the titles are in the plots in comparison to the x-axis. For example, is the plot labeled with the title “M1” an analysis which used M1 signal as signal A and ALM on the x-axis is signal B? Given that wPLI is not directed, there should be no difference between M1 (signal A) and ALM (signal B) versus ALM (signal A) and M1 (Signal B). Moreover, if there is phase synchrony between A24b and M1, as well as A24b and ALM, the ALM and M1 should have phase synchrony. Can the authors explain this? ALM and M1 are nearby and my have close to zero-lag phase locking which will not increase wPLI (see Vinck et al 2011). Therefore, wPLI may not be appropriate to use as the one single measure of network activity. Using additional measures (granger, transfer entropy) and looking for convergent findings across multiple methods would yield a more evidence and enable the authors to make more robust conclusions based on their data.

21. Page 11 - “global” connectivity. The authors need to clarify what this measure was and how it was calculated. It could not be understood from the manuscript in its current form.

22. Figure 6C - comparing strength across regions should use appropriate statistical test and the text can be modified where describing the results (see item 18 above).

23. Page 12 - the authors report no difference in power from 500-900ms post stimulus and then look at wPLI. However, for wPLI, they use a different window of 500-900ms post response. Why does the time window of interest change?

24. Page 12 - the authors report no difference in power from 500-900ms post stimulus. It is important to show the data instead of only stating the finding without reporting any numerical results in a text or figure that support this statement.

25. Page 12, logistic regression - the authors mention a few times “simple logistic regression”. Is there something different than a logistic regression?

26. Page 12 - the authors state that they “performed a simple logistic regression between connectivity and response time for each pair-wise interaction.” The exact procedure needs to be explained because, as written, the analysis is not clear. For example, was the regression looking at the relationship between the wPLI value for a pair of regions and the response time (a numeric value) or the trial type (a binary value)? Was the wPLI values first averaged across electrodes/sessions/rats within a brain region? What data points went into this logistic regression?

27. Results sub-section on “translational relevance” - The authors cannot translate directly to human “networks”. The brain regions are too different. This is not to say that some anatomy does not overlap or that there is no translational value in the data. However, I think translation could be made on the basis of finding that there are two networks (one with go-specific activity and one with wait-specific activity) in both species and/or that the relevant spectral frequencies overlap between species (go-delta, wait-theta). However, I do not think that the translation should be made on the basis of a few regions overlapping partly because the anatomy is too different and partly because the methods are different (human cortical EEG vs rat cortical and subcortical LFP).

28. However, if the authors were to make a translational link based on number of networks or frequency content (as explained above in comment #27), there is the larger issue that Figure 8 provides little evidence to support either claim. If the authors want to support this claim, it will be necessary to take the human data through the same steps as the rat data. What did response times look like in the human task and how does that justify time windows for EEG analysis? Analyze the power and WPLI in the same was in humans and rats. Show the same color plots from Figure 2A, B, C, D and Figure 5A, B, C for human data analyzed in the same way. The data need to be studied and presented in the same way in order to make a translational link.

29. How were the values plotted in Figure 2 (and later figures) for power? Is the color bar a z-score that has been session and rat averaged? As mentioned earlier, it would be helpful to have a clear explanation for how data were analyzed in the results. I understand from reading entire manuscript that the following steps occurred: (1) subtract baseline activity (defined as mean over time before stimulus) on each trial, (2) average across events within electrode (one electrode per region), (3) z-score (as mentioned above, unclear how z-score was calculated), (4) t-test each time-frequency point using a one-sided test against some mean (as mentioned above, it is not clear what this mean for the one-sided test was) with each data point being a session, (5) set all points in the spectrogram with non-significant values to a Z-score of 0 (what was the p-value threshold for this thresholding?), (6) average the data across sessions, (7) plot the data for example Figure 2A. Is this accurate? A graphical flow chart or sentence stating this succinctly in the results would greatly aid in the readability of the manuscript.

30. What is further unclear is how the go-wait contrast was calculated and how the “conjunction analysis” was done. How is a significant increase in Go determined? If significance was determined using one-sided t-tests for each time-frequency bin, then how does this translate into significant increase in Go trials? Was this done on each time-frequency bin or in bands?

31. How was the go-wait contrast calculated? Was the Z-scored spectral power in wait subtracted from the Z-scored spectral power in Go trials? Was this done on trial-averaged data? And then significance was determined for each time-frequency pair by one-sided t-test?

32. How was the conjunction of these two tests performed? A significance value for both Go and Go-wait? Was this conjunction used to set Z-score values to 0 in the plots in Figure 2A, B, C, D and similar plots in Figure 5?

33. I strongly disagree with changing values to 0 based on a statistical test. There are numerous reasons that a p-value could be above or below an arbitrary threshold that are unrelated to the question being studied. To then use that arbitrary threshold to zero-out data for plotting seems like an unnecessary step that radically affects the data. Can the authors just plot z-scores without this mask and change of values? Bands of interest in the spectrogram could still be visualized and then, for the purpose of statistical testing, the authors could get band-limited means like the line plots in Fig 2A, B, and C right panel and do ANOVA (time repeated measure) on those followed by post-hoc tests. An alternative would be to make a separate plot in which non-significant values are changed to NaN before getting mean and SEM. However, I think it is critical to show data without changing the values to NaN or 0, too. The same goes for wPLI analyses: I do not think it is apporpiate to change data to 0 and then plot it as the result.

34. Figure 2 - legend has a typo: “(significant differences between the two denoted with *. Using FDR-correction across....).” Also, I think that throughout the text, reading FDR-correction over and over is distracting and that stating it once in the methods is enough.

35. Figure 3 - title says 0-400msec but main text suggests 300-500msec

36. Figure 5 - there is in both A and B an increase in beta and gamma that seems to be higher in wait correct than in go. Can the authors examine this further?

37. Figure 5D - is the y-axis Z-scores? What are the *’s? A significant one-sided t-test against a mean of z-score = 0? A Z-score >2 is taken as 2 s.d. from the mean. A “significant” deviation in z-score from 0 with an effect size of only 0.6 seems to be an extremely small effect and the various significant findings may only be due to variability and high sample size (many sessions), whereas the actual change in the data is extremely small. Can the authors discuss this?

38. Figure 8C and D - what is -ve delta? This is not explained in the legend or results text. As mentioned above, it is critical to have Figure 8 follow same format as the rest of the manuscript and analyses of rat data to compare species.

39. Were any source localization (e.g., beam forming, etc.) measures performed on the human EEG to make it more localized to a region?

40. Discussion section, 1st paragraph - a novel way for characterizing rapid temporal dynamics was not established in this work. These methods are all previously established. The data set itself (31 regions from rat) is novel and the effort to obtain it is commendable.

41. “adapting an approach previously to characterize slower...” - I think the word “used” is missing. There are also instances of commas mis-used and was/were changes throughout this section

42. “specific patterns of connectivity was [were] associated with overall levels of behavioral inhibition” - I think overall level was not measured (e.g., relation between connectivity and how many trials, overall, were correctly inhibited)

43. “local-field-potentials” has dashes in discussion section and it did not before

44. “scalable, data-driven solution” - it was not clear what this phrase means

45. “more comprehensive approach” - more comprehensive in comparison to what?

46. “reversal learning paradigms, measuring the tendency” - “,which measure the tendency...” would be better. The rather long sentence has to be read a few times to understand it. And the next sentence uses commas and then switches to semi-colons part way through the sentence (in this case, commas are grammatically correct).

47. “It is well know that OFC contains a large diversity of neurons...” - Can phase relations with another brain region LFP or EEG somehow index different cell types and the relationship between their activity and different aspects of cognition?

48. “Here, by running the same task in humans...” - The task was not identical

49. “interpreting the LFPs without spiking activity is always tricky”. This statement reads as opinionated and non-scientific

50. “LFP can be used to map whole brain circuit physiology....” - I think it is nicely large-scale or multi-region but it is not whole brain like fMRI for example. Also, I think brain activity - behavior relationship was studied but not “circuit physiology”. The task was not particularly “complex” cognitive task.

51. Figure 1A has does not add anything to the paper. It would be useful to see the layout of the screen and nose poke holes, but the 3-d rendering occludes that useful information.

52. Raw data should be shown (some LFP traces during the task showing raw LFP and then overlaid filtered delta or theta during the various trial types and during windows of interest)
