# Supplementary material for: Mapping Large-Scale Networks Associated with Action, Behavioral Inhibition and Impulsivity
Source: eNeuro. 2021 Feb 23;8(1):ENEURO.0406-20.2021. doi: 10.1523/ENEURO.0406-20.2021 (PMC7920541; doi:10.1523/ENEURO.0406-20.2021)
Supplement: Extended Data Figure 3-1 — Mean activity from all brain regions filtered in delta frequencies (1-4 Hz) within the time window from 300-500ms post stimulus. p-values listed came from a two-sided, one-sample t-test, with null hypothesis (0), followed by Bonferonni adjustment of p-values (32 regions). Adjusted p-values > 1 were automatically set to 1. Data was estimated at the level of sessions (60). Download Figure 3-1, DOCX file. [file enu-eN-NWR-0406-20-s01.docx]

| **Delta Power (Go Correct)** | | | | **Delta Power (Go Cor - Wait Cor)** | | |
| --- | --- | --- | --- | --- | --- | --- |
| Electrodes | mean | SEM | p(FWE adj x 32) | mean | SEM | p(FWE adj x 32) |
| 'A32V' | 0.919 | 0.134 | 1.84E-07 | 0.092 | 0.169 | 1 |
| 'A32D' | 0.928 | 0.163 | 1.56E-05 | 0.196 | 0.184 | 1 |
| 'DMS' | 0.544 | 0.143 | 0.011984 | 0.102 | 0.147 | 1 |
| **'MDT'** | **0.662** | **0.154** | **0.002325** | **0.786** | **0.166** | **0.000519** |
| **'CMT'** | **0.882** | **0.151** | **9.32E-06** | **1.118** | **0.160** | **1.23E-07** |
| 'M2' | 0.906 | 0.188 | 0.000374 | 0.399 | 0.235 | 1 |
| 'LFC' | 0.941 | 0.143 | 5.16E-07 | 0.170 | 0.157 | 1 |
| **'ALM'** | **1.105** | **0.156** | **7.27E-08** | **0.784** | **0.170** | **0.000832** |
| **'M1'** | **1.631** | **0.165** | **1.89E-12** | **1.110** | **0.168** | **4.75E-07** |
| **'A33'** | **1.101** | **0.153** | **5.53E-08** | **0.829** | **0.158** | **7.91E-05** |
| **'A24a'** | **0.988** | **0.170** | **1.03E-05** | **0.688** | **0.179** | **0.010575** |
| **'A24b'** | **1.426** | **0.158** | **4.44E-11** | **0.747** | **0.190** | **0.008155** |
| 'STN' | 0.526 | 0.157 | 0.047882 | 0.392 | 0.154 | 0.446820 |
| 'DLS' | -0.078 | 0.132 | 1 | -0.155 | 0.155 | 1 |
| 'DLS'2 | -0.326 | 0.148 | 1 | -0.320 | 0.128 | 0.523765 |
| 'vOFC' | 0.562 | 0.146 | 0.010279 | 0.096 | 0.158 | 1 |
| 'L OFC' | 0.613 | 0.131 | 0.000658 | 0.038 | 0.155 | 1 |
| 'AIns' | 1.002 | 0.098 | 5.86E-13 | -0.206 | 0.130 | 1 |
| **'NAcS'** | **0.881** | **0.144** | **3.02E-06** | **0.786** | **0.130** | **4.20E-06** |
| 'NAcC' | 0.820 | 0.158 | 9.64E-05 | 0.547 | 0.164 | 0.052632 |
| 'VMS' | 0.845 | 0.162 | 9.05E-05 | 0.467 | 0.163 | 0.195210 |
| 'CEA' | -0.472 | 0.161 | 0.169104 | -0.204 | 0.174 | 1 |
| 'BLA' | -0.133 | 0.185 | 1 | 0.199 | 0.170 | 1 |
| **'V1'** | **0.762** | **0.142** | **5.28E-05** | **0.780** | **0.162** | **0.000389** |
| 'V1d’ | 0.233 | 0.128 | 1 | 0.729 | 0.139 | 8.76E-05 |
| **'PPCx'** | **0.488** | **0.144** | **0.044993** | **0.702** | **0.158** | **0.001391** |
| 'DS' | -0.198 | 0.126 | 1 | 0.676 | 0.144 | 0.000594 |
| 'DG' | 0.383 | 0.143 | 0.329724 | 0.661 | 0.156 | 0.002901 |
| 'CA1' | -0.016 | 0.109 | 1 | 0.559 | 0.099 | 1.80E-05 |
| 'CA3' | 0.243 | 0.104 | 0.779629 | 0.613 | 0.138 | 0.001503 |
| 'A30c' | 0.382 | 0.137 | 0.241039 | 0.637 | 0.156 | 0.004976 |
| 'A29c' | 0.392 | 0.120 | 0.063620 | 0.633 | 0.144 | 0.001634 |

**Figure 3-1:** Mean activity from all brain regions filtered in delta frequencies (1-4 Hz) within the time window from 300-500ms post stimulus. p-values listed came from a two-sided, one-sample t-test, with null hypothesis (0), followed by Bonferonni adjustment of p-values (32 regions). Adjusted p-values > 1 were automatically set to 1. Data was estimated at the level of sessions (60).
