# Supplementary material for: Mapping Large-Scale Networks Associated with Action, Behavioral Inhibition and Impulsivity
Source: eNeuro. 2021 Feb 23;8(1):ENEURO.0406-20.2021. doi: 10.1523/ENEURO.0406-20.2021 (PMC7920541; doi:10.1523/ENEURO.0406-20.2021)
Supplement: Extended Data Figure 3-2 — Mean activity from all brain regions filtered in delta frequencies (1-4 Hz) within the time window from 300-500ms post stimulus. p-values listed came from a two-sided, one-sample t-test, with null hypothesis (0). Data was estimated at the level of animals (n=11). Download Figure 3-2, DOCX file. [file enu-eN-NWR-0406-20-s02.docx]

| **Delta Power (Go Correct)** | | | |  | **Delta Power (Go Cor- Wait Cor)** | | |
| --- | --- | --- | --- | --- | --- | --- | --- |
| Electrodes | mean | SEM | p | | mean | SEM | p |
| 'A32V' | 1.218 | 0.314 | 0.004 | | 0.257 | 0.350 | 0.504 |
| 'A32D' | 1.142 | 0.315 | 0.006 | | -0.201 | 0.276 | 0.506 |
| 'DMS' | 0.675 | 0.448 | 0.182 | | 0.204 | 0.337 | 0.580 |
| **'MDT'** | **1.164** | **0.362** | **0.012** | | **1.431** | **0.449** | **0.014** |
| **'CMT'** | **1.125** | **0.368** | **0.015** | | **1.324** | **0.345** | **0.005** |
| 'M2' | 1.167 | 0.419 | 0.024 | | -0.020 | 0.435 | 0.966 |
| 'LFC' | 1.217 | 0.337 | 0.006 | | 0.157 | 0.372 | 0.698 |
| 'ALM' | 1.131 | 0.477 | 0.047 | | 0.464 | 0.455 | 0.358 |
| 'M1' | 1.650 | 0.371 | 0.002 | | 0.717 | 0.395 | 0.120 |
| **'A33'** | **1.386** | **0.313** | **0.002** | | **0.789** | **0.313** | **0.040** |
| **'A24a'** | **1.426** | **0.352** | **0.003** | | **0.747** | **0.303** | **0.044** |
| 'A24b' | 1.600 | 0.354 | 0.002 | | 0.353 | 0.342 | 0.353 |
| 'STN' | 0.800 | 0.440 | 0.113 | | 0.285 | 0.294 | 0.382 |
| 'DLS' | 0.131 | 0.325 | 0.708 | | 0.094 | 0.201 | 0.666 |
| 'DLS' | -0.314 | 0.384 | 0.454 | | -0.295 | 0.223 | 0.242 |
| 'vOFC' | 0.579 | 0.435 | 0.233 | | 0.118 | 0.355 | 0.760 |
| 'L OFC' | 0.775 | 0.367 | 0.072 | | 0.015 | 0.360 | 0.969 |
| 'AIns' | 1.270 | 0.241 | 0.001 | | -0.393 | 0.212 | 0.113 |
| **'NAcS'** | **0.996** | **0.343** | **0.020** | | **0.617** | **0.223** | **0.028** |
| 'NAcC' | 1.092 | 0.427 | 0.035 | | 0.622 | 0.403 | 0.177 |
| 'VMS' | 1.040 | 0.432 | 0.044 | | 0.636 | 0.380 | 0.147 |
| 'CEA' | -0.657 | 0.383 | 0.133 | | -0.191 | 0.307 | 0.569 |
| 'BLA' | -0.229 | 0.433 | 0.626 | | 0.167 | 0.345 | 0.656 |
| **'V1'** | **0.935** | **0.325** | **0.021** | | **1.026** | **0.403** | **0.039** |
| 'V1' | 0.886 | 0.416 | 0.070 | | 1.583 | 0.525 | 0.019 |
| 'PPCx' | 0.658 | 0.421 | 0.167 | | 1.085 | 0.330 | 0.012 |
| 'DS' | -0.336 | 0.443 | 0.486 | | 1.011 | 0.445 | 0.060 |
| 'DG' | 0.726 | 0.481 | 0.181 | | 1.019 | 0.317 | 0.014 |
| 'CA1' | 0.258 | 0.334 | 0.478 | | 0.941 | 0.192 | 0.001 |
| 'CA3' | 0.462 | 0.266 | 0.129 | | 0.853 | 0.312 | 0.029 |
| 'A30c' | 0.540 | 0.275 | 0.090 | | 0.860 | 0.269 | 0.014 |
| 'A29c' | 0.450 | 0.267 | 0.139 | | 0.821 | 0.291 | 0.026 |

**Figure 3-2:** Mean activity from all brain regions filtered in delta frequencies (1-4 Hz) within the time window from 300-500ms post stimulus. p-values listed came from a two-sided, one-sample t-test, with null hypothesis (0). Data was estimated at the level of animals (n=11).
