# Supplementary material for: Mapping Large-Scale Networks Associated with Action, Behavioral Inhibition and Impulsivity
Source: eNeuro. 2021 Feb 23;8(1):ENEURO.0406-20.2021. doi: 10.1523/ENEURO.0406-20.2021 (PMC7920541; doi:10.1523/ENEURO.0406-20.2021)
Supplement: Extended Data Figure 4-1 — We calculated the pair-wise weighted phase-lagged-index from the above regions on at a session by session level for both correct wait and correct go trials. We then estimated the mean/SEM of the pair-wise wPLI difference at delta frequencies (1 -4 Hz) from 300-500ms post-stimulus onset. p-values were calculated using a one-sample two-tailed t-test on this difference (null hypothesis is that there was no difference). P-values were then adjusted using an FDR-correction. Download Figure 4-1, DOCX file. [file enu-eN-NWR-0406-20-s03.docx]

| **Mean** | | | |  |  |  |  | |  | |  | |  | |  | |  | |  |
| --- | --- | --- | --- | --- | --- | --- | --- | --- | --- | --- | --- | --- | --- | --- | --- | --- | --- | --- | --- |
|  | **M1** | **ALM** | **A24b** | **'24a** | **A33** | **V1** | | **MDT** | | **CMT** | | **NAcS** | | **PPCx** | | **DG** | | **A30c** | |
| **M1** | NaN | 0.074 | 0.112 | 0.005 | 0.030 | 0.020 | | -0.020 | | -0.007 | | -0.012 | | 0.063 | | -0.011 | | 0.017 | |
| **ALM** | 0.074 | NaN | 0.086 | -0.050 | -0.095 | 0.015 | | 0.046 | | 0.041 | | -0.007 | | -0.026 | | 0.014 | | 0.008 | |
| **A24b** | 0.112 | 0.086 | NaN | -0.047 | 0.023 | 0.027 | | 0.043 | | -0.016 | | 0.053 | | -0.076 | | -0.017 | | -0.008 | |
| **A24a** | 0.005 | -0.050 | -0.047 | NaN | 0.045 | 0.073 | | 0.049 | | -0.022 | | 0.118 | | 0.028 | | 0.022 | | 0.075 | |
| **A33** | 0.030 | -0.095 | 0.023 | 0.045 | NaN | 0.052 | | 0.065 | | 0.077 | | 0.079 | | 0.053 | | 0.047 | | 0.107 | |
| **V1** | 0.020 | 0.015 | 0.027 | 0.073 | 0.052 | NaN | | 0.023 | | 0.090 | | 0.111 | | 0.124 | | 0.123 | | 0.040 | |
| **MDT** | -0.020 | 0.046 | 0.043 | 0.049 | 0.065 | 0.023 | | NaN | | 0.139 | | 0.114 | | 0.063 | | 0.068 | | 0.087 | |
| **CMT** | -0.007 | 0.041 | -0.016 | -0.022 | 0.077 | 0.090 | | 0.139 | | NaN | | 0.086 | | 0.021 | | 0.043 | | 0.077 | |
| **NAcS** | -0.012 | -0.007 | 0.053 | 0.118 | 0.079 | 0.111 | | 0.114 | | 0.086 | | NaN | | 0.068 | | 0.115 | | 0.065 | |
| **PPCx** | 0.063 | -0.026 | -0.076 | 0.028 | 0.053 | 0.124 | | 0.063 | | 0.021 | | 0.068 | | NaN | | 0.088 | | -0.021 | |
| **DG** | -0.011 | 0.014 | -0.017 | 0.022 | 0.047 | 0.123 | | 0.068 | | 0.043 | | 0.115 | | 0.088 | | NaN | | 0.054 | |
| **A30c** | 0.017 | 0.008 | -0.008 | 0.075 | 0.107 | 0.040 | | 0.087 | | 0.077 | | 0.065 | | -0.021 | | 0.054 | | NaN | |
| **A29c** | -0.025 | 0.028 | -0.032 | 0.027 | 0.047 | 0.080 | | -0.009 | | 0.054 | | -0.014 | | 0.010 | | 0.030 | | 0.086 | |
| **SEM** | | |  |  |  |  |  | |  | |  | |  | |  | |  | |  |
|  | **M1** | **ALM** | **A24b** | **A24a** | **A33** | **V1** | | **MDT** | | **CMT** | | **NAcS** | | **PPCx** | | **DG** | | **A30c** | |
| **M1** | NaN | 0.022 | 0.020 | 0.023 | 0.018 | 0.027 | | 0.024 | | 0.013 | | 0.021 | | 0.023 | | 0.024 | | 0.019 | |
| **ALM** | 0.022 | NaN | 0.020 | 0.022 | 0.033 | 0.017 | | 0.029 | | 0.022 | | 0.027 | | 0.019 | | 0.021 | | 0.025 | |
| **A24b** | 0.020 | 0.020 | NaN | 0.020 | 0.023 | 0.020 | | 0.027 | | 0.016 | | 0.030 | | 0.025 | | 0.025 | | 0.015 | |
| **A24a** | 0.023 | 0.022 | 0.020 | NaN | 0.028 | 0.021 | | 0.023 | | 0.029 | | 0.027 | | 0.018 | | 0.017 | | 0.021 | |
| **A33** | 0.018 | 0.033 | 0.023 | 0.028 | NaN | 0.021 | | 0.024 | | 0.031 | | 0.026 | | 0.022 | | 0.019 | | 0.024 | |
| **V1** | 0.027 | 0.017 | 0.020 | 0.021 | 0.021 | NaN | | 0.021 | | 0.021 | | 0.025 | | 0.022 | | 0.024 | | 0.012 | |
| **MDT** | 0.024 | 0.029 | 0.027 | 0.023 | 0.024 | 0.021 | | NaN | | 0.036 | | 0.033 | | 0.023 | | 0.024 | | 0.028 | |
| **CMT** | 0.013 | 0.022 | 0.016 | 0.029 | 0.031 | 0.021 | | 0.036 | | NaN | | 0.024 | | 0.026 | | 0.019 | | 0.029 | |
| **NAcS** | 0.021 | 0.027 | 0.030 | 0.027 | 0.026 | 0.025 | | 0.033 | | 0.024 | | NaN | | 0.022 | | 0.028 | | 0.025 | |
| **PPCx** | 0.023 | 0.019 | 0.025 | 0.018 | 0.022 | 0.022 | | 0.023 | | 0.026 | | 0.022 | | NaN | | 0.022 | | 0.016 | |
| **DG** | 0.024 | 0.021 | 0.025 | 0.017 | 0.019 | 0.024 | | 0.024 | | 0.019 | | 0.028 | | 0.022 | | NaN | | 0.016 | |
| **A30c** | 0.019 | 0.025 | 0.015 | 0.021 | 0.024 | 0.012 | | 0.028 | | 0.029 | | 0.025 | | 0.016 | | 0.016 | | NaN | |
| **A29c** | 0.013 | 0.024 | 0.017 | 0.019 | 0.028 | 0.017 | | 0.038 | | 0.031 | | 0.030 | | 0.020 | | 0.020 | | 0.021 | |
| **p-values** | |  |  |  |  |  | |  | |  | |  | |  | |  | |  | |
|  | **M1** | **ALM** | **A24b** | **A24a** | **A33** | **V1** | | **MDT** | | **CMT** | | **NAcS** | | **PPCx** | | **DG** | | **A30c** | |
| **M1** | NaN | **0.008** | **0.000** | 0.897 | 0.175 | 0.602 | | 0.541 | | 0.717 | | 0.707 | | **0.024** | | 0.757 | | 0.526 | |
| **ALM** | **0.008** | NaN | **0.001** | 0.066 | **0.020** | 0.526 | | 0.210 | | 0.139 | | 0.890 | | 0.299 | | 0.629 | | 0.851 | |
| **A24b** | 0.000 | **0.001** | NaN | 0.062 | 0.447 | 0.299 | | 0.213 | | 0.447 | | 0.164 | | **0.014** | | 0.620 | | 0.707 | |
| **A24a** | 0.897 | 0.066 | 0.062 | NaN | 0.213 | **0.006** | | 0.090 | | 0.591 | | **0.001** | | 0.233 | | 0.320 | | **0.004** | |
| **A33** | 0.175 | **0.020** | 0.447 | 0.213 | NaN | **0.040** | | **0.028** | | **0.040** | | **0.014** | | **0.044** | | **0.040** | | **0.001** | |
| **V1** | 0.602 | 0.526 | 0.299 | **0.006** | **0.040** | NaN | | 0.407 | | **0.001** | | **0.001** | | **0.000** | | **0.000** | | **0.008** | |
| **MDT** | 0.541 | 0.210 | 0.213 | 0.090 | **0.028** | 0.407 | | NaN | | **0.002** | | **0.006** | | **0.027** | | **0.020** | | **0.011** | |
| **CMT** | 0.717 | 0.139 | 0.447 | 0.591 | **0.040** | **0.001** | | **0.002** | | NaN | | **0.005** | | 0.576 | | 0.066 | | **0.029** | |
| **NAcS** | 0.707 | 0.890 | 0.164 | **0.001** | **0.014** | **0.001** | | **0.006** | | **0.005** | | NaN | | **0.011** | | **0.001** | | **0.030** | |
| **PPCx** | **0.024** | 0.299 | **0.014** | 0.233 | **0.044** | **0.000** | | **0.027** | | 0.576 | | **0.011** | | NaN | | **0.002** | | 0.312 | |
| **DG** | 0.757 | 0.629 | 0.620 | 0.320 | 0.040 | 0.000 | | 0.020 | | 0.066 | | 0.001 | | 0.002 | | NaN | | 0.006 | |
| **A30c** | 0.526 | 0.851 | 0.707 | **0.004** | **0.001** | **0.008** | | **0.011** | | **0.029** | | **0.030** | | 0.312 | | **0.006** | | NaN | |
| **A29c** | 0.125 | 0.361 | 0.125 | 0.261 | 0.175 | **0.000** | | 0.890 | | 0.175 | | 0.738 | | 0.717 | | 0.249 | | **0.001** | |

**Figure 4_1:** We calculated the pair-wise weighted phase-lagged-index from the above regions on at a session by session level for both correct wait and correct go trials. We then estimated the mean/SEM of the pair-wise wPLI difference at delta frequencies (1 -4 Hz) from 300-500ms post-stimulus onset. p-values were calculated using a one-sample two-tailed t-test on this difference (null hypothesis is that there was no difference). P-values were then adjusted using an FDR-correction.
