# Supplementary material for: Mapping Large-Scale Networks Associated with Action, Behavioral Inhibition and Impulsivity
Source: eNeuro. 2021 Feb 23;8(1):ENEURO.0406-20.2021. doi: 10.1523/ENEURO.0406-20.2021 (PMC7920541; doi:10.1523/ENEURO.0406-20.2021)
Supplement: Extended Data Figure 6-1 — Mean theta power from electrodes for correct wait trials alone, and from the difference (correct wait trials - correct go trials), data taken from 500-2000ms post-stimulus. Mean/SEM calculated at the level of sessions (60 sessions). We used a one-sample, two-sided t-test for both analyses (null hypothesis that power = 0). p-values were adjusted for multiple corrections using Bonferroni adjustment (32 regions). Bold names are highlighted that are significant for both analyses, suggesting involvement in inhibition. Download Figure 6-1, DOCX file. [file enu-eN-NWR-0406-20-s04.docx]

| **Theta Power (Wait Correct)** | | |  |  | **Theta Power (Wait Cor - Go Cor)** | | |
| --- | --- | --- | --- | --- | --- | --- | --- |
| Electrodes | mean | SEM | adj. p-value |  | mean | SEM | adj. p value |
| **A32V** | **0.650** | **0.103** | **1.612E-06** |  | **1.067** | **0.105** | **5.684E-13** |
| **A32D** | **0.600** | **0.095** | **1.506E-06** |  | **1.048** | **0.113** | **1.855E-11** |
| DMS | 0.381 | 0.109 | 3.242E-02 |  | 0.968 | 0.125 | 6.613E-09 |
| MDT | 0.302 | 0.113 | 3.246E-01 |  | 0.955 | 0.129 | 2.127E-08 |
| CMT | 0.298 | 0.116 | 4.390E-01 |  | 1.015 | 0.120 | 4.358E-10 |
| **M2** | **0.385** | **0.098** | **7.682E-03** |  | **0.743** | **0.131** | **1.649E-05** |
| **LFC** | **0.548** | **0.098** | **2.068E-05** |  | **0.492** | **0.120** | **4.719E-03** |
| **ALM** | **0.341** | **0.099** | **3.613E-02** |  | **0.439** | **0.126** | **3.370E-02** |
| **M1** | **0.299** | **0.084** | **2.494E-02** |  | **1.100** | **0.127** | **1.758E-10** |
| **A33** | **0.386** | **0.110** | **2.888E-02** |  | **0.785** | **0.141** | **2.721E-05** |
| A24a | 0.259 | 0.104 | 5.340E-01 |  | 1.008 | 0.119 | 3.497E-10 |
| **A24b** | **0.414** | **0.090** | **8.254E-04** |  | **1.124** | **0.124** | **3.816E-11** |
| STN | 0.306 | 0.112 | 2.802E-01 |  | 0.959 | 0.125 | 8.764E-09 |
| **DLSd** | **0.543** | **0.093** | **9.577E-06** |  | **0.806** | **0.135** | **5.162E-06** |
| **DLSv** | **0.485** | **0.082** | **6.685E-06** |  | **0.800** | **0.117** | **2.128E-07** |
| **vOFC** | **0.708** | **0.104** | **2.211E-07** |  | **1.004** | **0.102** | **2.416E-12** |
| **L OFC** | **0.495** | **0.098** | **1.753E-04** |  | **0.452** | **0.135** | **4.792E-02** |
| **AIns** | **0.790** | **0.094** | **4.207E-10** |  | **0.639** | **0.117** | **3.846E-05** |
| NAcS | 0.212 | 0.104 | 1.000E+00 |  | 0.432 | 0.111 | 9.054E-03 |
| **NAcC** | **0.371** | **0.101** | **1.780E-02** |  | **0.760** | **0.113** | **3.477E-07** |
| **VMS** | **0.497** | **0.093** | **5.520E-05** |  | **0.858** | **0.098** | **1.202E-10** |
| **CEA** | **0.395** | **0.089** | **1.395E-03** |  | **0.839** | **0.123** | **1.979E-07** |
| BLA | 0.216 | 0.100 | 1.000E+00 |  | 0.545 | 0.122 | 1.318E-03 |
| V1d | -0.218 | 0.096 | 8.818E-01 |  | 0.996 | 0.094 | 9.948E-14 |
| V1v | 0.134 | 0.096 | 1.000E+00 |  | 0.941 | 0.101 | 1.486E-11 |
| PPCx | 0.116 | 0.099 | 1.000E+00 |  | 1.021 | 0.101 | 8.704E-13 |
| DS | -0.141 | 0.087 | 1.000E+00 |  | 1.016 | 0.096 | 1.172E-13 |
| DG | 0.213 | 0.112 | 1.000E+00 |  | 0.869 | 0.114 | 8.603E-09 |
| CA1 | 0.058 | 0.090 | 1.000E+00 |  | 0.970 | 0.108 | 4.542E-11 |
| CA3 | 0.010 | 0.092 | 1.000E+00 |  | 0.956 | 0.092 | 3.162E-13 |
| A30c | 0.143 | 0.098 | 1.000E+00 |  | 0.982 | 0.095 | 3.446E-13 |
| A29c | 0.188 | 0.099 | 1.000E+00 |  | 1.030 | 0.120 | 2.265E-10 |

**Theta Activity (500 - 2000ms post-stimulus, session level stats)**

**Figure 6_1:** Mean theta power from electrodes for correct wait trials alone, and from the difference (correct wait trials – correct go trials), data taken from 500-2000ms post-stimulus. Mean/SEM calculated at the level of sessions (60 sessions). We used a one-sample, two-sided t-test for both analyses (null hypothesis that power = 0). p-values were adjusted for multiple corrections using Bonferroni adjustment (32 regions). Bold names are highlighted that are significant for both analyses, suggesting involvement in inhibition.
