# Supplementary material for: Mapping Large-Scale Networks Associated with Action, Behavioral Inhibition and Impulsivity
Source: eNeuro. 2021 Feb 23;8(1):ENEURO.0406-20.2021. doi: 10.1523/ENEURO.0406-20.2021 (PMC7920541; doi:10.1523/ENEURO.0406-20.2021)
Supplement: Extended Data Figure 6-2 — Mean theta power from electrodes for correct wait trials alone, and from the difference (correct wait trials - correct go trials), data taken from 500-2000ms post-stimulus. Mean/SEM calculated at the level of animals (11 animals). We used a one-sample, two-sided t-test for both analyses (null hypothesis that power = 0). Bold names are highlighted that are significant for both analyses, suggesting involvement in inhibition. Download Figure 6-2, DOCX file. [file enu-eN-NWR-0406-20-s05.docx]

**Theta Activity (500 - 2000ms post-stimulus, animal level stats)**

| **Theta Power (Wait Cor)** | | | |  | **Theta Power (Wait Cor - Go Cor)** | | |
| --- | --- | --- | --- | --- | --- | --- | --- |
| Electrodes | mean | SEM | adj. p val |  | mean | SEM | adj. p val |
| **A32V** | **0.623** | **0.265** | **0.046** |  | **1.241** | **0.281** | **1.418E-03** |
| **A32D** | **0.682** | **0.230** | **0.016** |  | **1.247** | **0.323** | **3.531E-03** |
| **DMS** | **0.581** | **0.237** | **0.039** |  | **1.525** | **0.219** | **3.548E-05** |
| MDT | 0.342 | 0.233 | 0.188 |  | 1.546 | 0.129 | 1.900E-07 |
| CMT | 0.282 | 0.270 | 0.339 |  | 1.240 | 0.203 | 1.089E-04 |
| **M2** | **0.486** | **0.206** | **0.045** |  | **0.929** | **0.286** | **9.936E-03** |
| **LFC** | **0.696** | **0.152** | **0.001** |  | **0.721** | **0.267** | **2.514E-02** |
| ALM | 0.338 | 0.201 | 0.136 |  | 0.521 | 0.227 | 5.072E-02 |
| M1 | 0.342 | 0.185 | 0.104 |  | 1.397 | 0.238 | 1.534E-04 |
| A33 | 0.501 | 0.226 | 0.058 |  | 0.959 | 0.304 | 1.176E-02 |
| A24a | 0.413 | 0.192 | 0.064 |  | 1.297 | 0.201 | 6.889E-05 |
| **A24b** | 0.588 | 0.160 | 0.005 |  | 1.465 | 0.247 | 1.413E-04 |
| STN | 0.327 | 0.235 | 0.210 |  | 1.355 | 0.216 | 8.987E-05 |
| **DLSd** | **0.682** | **0.212** | **0.010** |  | **1.347** | **0.284** | **8.457E-04** |
| **DLSv** | **0.734** | **0.170** | **0.002** |  | **1.292** | **0.225** | **1.859E-04** |
| **vOFC** | **0.868** | **0.205** | **0.002** |  | **1.325** | **0.262** | **5.196E-04** |
| l OFC | 0.512 | 0.234 | 0.060 |  | 0.538 | 0.344 | 1.625E-01 |
| **A Ins** | **0.883** | **0.186** | **0.001** |  | **0.779** | **0.301** | **3.073E-02** |
| NAcS | 0.242 | 0.227 | 0.330 |  | 0.539 | 0.230 | 4.663E-02 |
| **NacC** | **0.541** | **0.174** | **0.013** |  | **1.197** | **0.186** | **6.937E-05** |
| **VMS** | **0.699** | **0.182** | **0.004** |  | **1.257** | **0.161** | **1.226E-05** |
| **CEA** | **0.495** | **0.198** | **0.036** |  | **1.228** | **0.259** | **8.474E-04** |
| BLA | 0.352 | 0.222 | 0.157 |  | 0.942 | 0.209 | 1.230E-03 |
| V1d | -0.331 | 0.170 | 0.088 |  | 1.353 | 0.153 | 3.700E-06 |
| V1v | 0.188 | 0.164 | 0.296 |  | 1.577 | 0.187 | 6.080E-06 |
| PPCx | 0.112 | 0.228 | 0.648 |  | 1.353 | 0.165 | 7.895E-06 |
| DS | -0.284 | 0.181 | 0.162 |  | 1.206 | 0.239 | 5.261E-04 |
| DG | 0.331 | 0.265 | 0.257 |  | 1.399 | 0.239 | 1.615E-04 |
| CA1 | 0.060 | 0.134 | 0.676 |  | 1.258 | 0.188 | 4.965E-05 |
| CA3 | 0.034 | 0.187 | 0.865 |  | 1.424 | 0.148 | 1.703E-06 |
| A30c | 0.193 | 0.256 | 0.486 |  | 1.295 | 0.178 | 2.316E-05 |
| A29c | 0.229 | 0.198 | 0.291 |  | 1.389 | 0.214 | 6.513E-05 |

**Figure 6_2:** Mean theta power from electrodes for correct wait trials alone, and from the difference (correct wait trials – correct go trials), data taken from 500-2000ms post-stimulus. Mean/SEM calculated at the level of animals (11 animals). We used a one-sample, two-sided t-test for both analyses (null hypothesis that power = 0). Bold names are highlighted that are significant for both analyses, suggesting involvement in inhibition.
