# Supplementary material for: Mapping Large-Scale Networks Associated with Action, Behavioral Inhibition and Impulsivity
Source: eNeuro. 2021 Feb 23;8(1):ENEURO.0406-20.2021. doi: 10.1523/ENEURO.0406-20.2021 (PMC7920541; doi:10.1523/ENEURO.0406-20.2021)
Supplement: Extended Data Figure 7-1 — Data table for mean, SEM and p-values for pair-wise wPLI. Data taken at theta-frequencies between 500-2000ms post-stimulus. P-values adjusted for multiple comparisons using FDR-correction. Download Figure 7-1, DOCX file. [file enu-eN-NWR-0406-20-s06.docx]

Mean Theta wPLI (500-2000ms), Wait Trials, electrodes 1:16

| Electrodes | **A32V** | **A32D** | **DMS** | **MDT** | **CMT** | **M2** | **LFC** | **ALM** | **M1** | **A33** | **A24a** | **A24b** | **STN** | **DLSd** | **DLSv** | **vOFC** |
| --- | --- | --- | --- | --- | --- | --- | --- | --- | --- | --- | --- | --- | --- | --- | --- | --- |
| **A32V** | NaN | 0.12 | 0.07 | 0.04 | 0.03 | 0.16 | 0.22 | 0.11 | 0.07 | 0.05 | 0.02 | 0.04 | 0.03 | 0.05 | 0.05 | 0.08 |
| **A32D** | 0.12 | NaN | 0.09 | -0.01 | -0.01 | 0.06 | 0.10 | 0.01 | 0.03 | 0.08 | 0.04 | 0.00 | 0.02 | 0.01 | 0.00 | 0.08 |
| **DMS** | 0.07 | 0.09 | NaN | 0.07 | 0.11 | 0.09 | 0.12 | 0.08 | 0.15 | 0.08 | 0.08 | 0.09 | 0.10 | 0.10 | 0.09 | 0.03 |
| **MDT** | 0.04 | -0.01 | 0.07 | NaN | 0.00 | -0.01 | 0.05 | -0.01 | 0.00 | 0.04 | 0.07 | 0.04 | 0.03 | -0.02 | 0.01 | 0.06 |
| **CMT** | 0.03 | -0.01 | 0.11 | 0.00 | NaN | 0.05 | 0.06 | 0.02 | 0.02 | 0.07 | 0.07 | 0.05 | 0.05 | -0.02 | 0.01 | 0.07 |
| **M2** | 0.16 | 0.06 | 0.09 | -0.01 | 0.05 | NaN | 0.07 | 0.06 | -0.01 | 0.15 | 0.16 | 0.04 | 0.06 | 0.04 | 0.04 | 0.11 |
| **LFC** | 0.22 | 0.10 | 0.12 | 0.05 | 0.06 | 0.07 | NaN | 0.11 | 0.02 | 0.11 | 0.11 | 0.06 | 0.09 | 0.08 | 0.07 | 0.16 |
| **ALM** | 0.11 | 0.01 | 0.08 | -0.01 | 0.02 | 0.06 | 0.11 | NaN | 0.02 | 0.07 | 0.05 | 0.02 | 0.05 | 0.01 | 0.03 | 0.07 |
| **M1** | 0.07 | 0.03 | 0.15 | 0.00 | 0.02 | -0.01 | 0.02 | 0.02 | NaN | 0.11 | 0.07 | 0.03 | 0.04 | -0.01 | 0.02 | 0.11 |
| **A33** | 0.05 | 0.08 | 0.08 | 0.04 | 0.07 | 0.15 | 0.11 | 0.07 | 0.11 | NaN | 0.00 | 0.04 | 0.07 | 0.08 | 0.08 | 0.06 |
| **A24a** | 0.02 | 0.04 | 0.08 | 0.07 | 0.07 | 0.16 | 0.11 | 0.05 | 0.07 | 0.00 | NaN | 0.04 | 0.03 | 0.06 | 0.06 | 0.08 |
| **A24b** | 0.04 | 0.00 | 0.09 | 0.04 | 0.05 | 0.04 | 0.06 | 0.02 | 0.03 | 0.04 | 0.04 | NaN | 0.05 | 0.01 | 0.03 | 0.09 |
| **STN** | 0.03 | 0.02 | 0.10 | 0.03 | 0.05 | 0.06 | 0.09 | 0.05 | 0.04 | 0.07 | 0.03 | 0.05 | NaN | 0.02 | 0.07 | 0.06 |
| **DLSd** | 0.05 | 0.01 | 0.10 | -0.02 | -0.02 | 0.04 | 0.08 | 0.01 | -0.01 | 0.08 | 0.06 | 0.01 | 0.02 | NaN | 0.02 | 0.09 |
| **DLSv** | 0.05 | 0.00 | 0.09 | 0.01 | 0.01 | 0.04 | 0.07 | 0.03 | 0.02 | 0.08 | 0.06 | 0.03 | 0.07 | 0.02 | NaN | 0.09 |
| **vOFC** | 0.08 | 0.08 | 0.03 | 0.06 | 0.07 | 0.11 | 0.16 | 0.07 | 0.11 | 0.06 | 0.08 | 0.09 | 0.06 | 0.09 | 0.09 | NaN |
| **l OFC** | 0.10 | 0.11 | 0.00 | 0.08 | 0.10 | 0.14 | 0.13 | 0.09 | 0.03 | 0.03 | 0.06 | 0.04 | 0.06 | 0.11 | 0.11 | 0.07 |
| **A Ins** | 0.18 | 0.08 | 0.10 | 0.04 | 0.07 | 0.08 | 0.01 | 0.09 | 0.02 | 0.08 | 0.09 | 0.05 | 0.08 | 0.07 | 0.07 | 0.14 |
| **NAcS** | 0.03 | 0.03 | 0.00 | 0.05 | 0.05 | 0.04 | 0.07 | 0.04 | 0.02 | 0.02 | 0.03 | 0.01 | 0.07 | 0.08 | 0.07 | 0.05 |
| **NacC** | 0.06 | 0.03 | 0.05 | 0.02 | 0.03 | 0.03 | 0.08 | 0.02 | 0.09 | 0.05 | 0.06 | 0.06 | 0.05 | 0.07 | 0.06 | 0.05 |
| **VMS** | 0.13 | 0.14 | 0.07 | 0.11 | 0.14 | 0.12 | 0.20 | 0.11 | 0.17 | 0.14 | 0.14 | 0.14 | 0.16 | 0.20 | 0.16 | 0.08 |
| **CEA** | 0.06 | 0.01 | 0.08 | 0.02 | 0.03 | 0.04 | 0.04 | 0.05 | 0.04 | 0.10 | 0.08 | 0.06 | 0.08 | 0.03 | 0.03 | 0.08 |
| **BLA** | 0.00 | -0.01 | 0.04 | 0.01 | -0.03 | 0.03 | 0.00 | 0.03 | 0.03 | 0.02 | 0.04 | 0.04 | 0.05 | 0.04 | 0.05 | 0.02 |
| **V1d** | 0.01 | 0.04 | 0.09 | -0.03 | -0.01 | 0.03 | 0.07 | 0.01 | 0.07 | 0.04 | 0.03 | 0.05 | -0.01 | 0.01 | 0.04 | 0.01 |
| **V1v** | 0.00 | 0.03 | 0.06 | -0.03 | -0.01 | 0.01 | 0.04 | 0.01 | 0.02 | 0.03 | 0.04 | 0.02 | -0.01 | 0.02 | 0.04 | 0.00 |
| **PPCx** | 0.00 | 0.01 | 0.06 | -0.03 | 0.02 | 0.03 | 0.07 | 0.02 | 0.04 | 0.03 | 0.02 | 0.03 | 0.00 | 0.03 | 0.04 | 0.03 |
| **DS** | 0.01 | 0.02 | 0.08 | -0.03 | 0.00 | 0.05 | 0.06 | 0.01 | 0.01 | 0.02 | 0.00 | 0.00 | 0.02 | 0.01 | 0.04 | 0.02 |
| **DG** | 0.02 | 0.03 | 0.08 | 0.01 | 0.07 | 0.06 | 0.06 | 0.02 | 0.07 | 0.01 | 0.03 | 0.06 | 0.04 | 0.02 | 0.05 | 0.03 |
| **CA1** | 0.00 | 0.01 | 0.07 | -0.07 | 0.01 | 0.01 | 0.00 | 0.00 | 0.01 | 0.03 | 0.00 | -0.04 | 0.00 | 0.00 | 0.01 | 0.05 |
| **CA3** | 0.02 | -0.01 | 0.12 | 0.03 | 0.08 | 0.00 | 0.04 | 0.00 | 0.03 | 0.10 | 0.09 | 0.06 | 0.06 | 0.01 | 0.02 | 0.09 |
| **A30c** | 0.00 | 0.00 | 0.04 | -0.01 | -0.01 | 0.01 | 0.07 | 0.03 | 0.05 | 0.02 | 0.02 | 0.07 | 0.04 | 0.01 | 0.04 | 0.03 |
| **A29c** | 0.03 | 0.03 | 0.10 | -0.02 | 0.00 | 0.03 | 0.04 | 0.03 | 0.09 | 0.07 | 0.07 | 0.09 | 0.06 | 0.02 | 0.02 | 0.07 |

Mean Theta wPLI (500-2000ms), Wait Trials, electrodes 17:32

| Electrodes | l OFC | A Ins | NAcS | NacC | VMS | CEA | BLA | V1d | V1v | PPCx | DS | DG | CA1 | CA3 | A30c | A29c |
| --- | --- | --- | --- | --- | --- | --- | --- | --- | --- | --- | --- | --- | --- | --- | --- | --- |
| A32V | 0.10 | 0.18 | 0.03 | 0.06 | 0.13 | 0.06 | 0.00 | 0.01 | 0.00 | 0.00 | 0.01 | 0.02 | 0.00 | 0.02 | 0.00 | 0.03 |
| A32D | 0.11 | 0.08 | 0.03 | 0.03 | 0.14 | 0.01 | -0.01 | 0.04 | 0.03 | 0.01 | 0.02 | 0.03 | 0.01 | -0.01 | 0.00 | 0.03 |
| DMS | 0.00 | 0.10 | 0.00 | 0.05 | 0.07 | 0.08 | 0.04 | 0.09 | 0.06 | 0.06 | 0.08 | 0.08 | 0.07 | 0.12 | 0.04 | 0.10 |
| MDT | 0.08 | 0.04 | 0.05 | 0.02 | 0.11 | 0.02 | 0.01 | -0.03 | -0.03 | -0.03 | -0.03 | 0.01 | -0.07 | 0.03 | -0.01 | -0.02 |
| CMT | 0.10 | 0.07 | 0.05 | 0.03 | 0.14 | 0.03 | -0.03 | -0.01 | -0.01 | 0.02 | 0.00 | 0.07 | 0.01 | 0.08 | -0.01 | 0.00 |
| M2 | 0.14 | 0.08 | 0.04 | 0.03 | 0.12 | 0.04 | 0.03 | 0.03 | 0.01 | 0.03 | 0.05 | 0.06 | 0.01 | 0.00 | 0.01 | 0.03 |
| LFC | 0.13 | 0.01 | 0.07 | 0.08 | 0.20 | 0.04 | 0.00 | 0.07 | 0.04 | 0.07 | 0.06 | 0.06 | 0.00 | 0.04 | 0.07 | 0.04 |
| ALM | 0.09 | 0.09 | 0.04 | 0.02 | 0.11 | 0.05 | 0.03 | 0.01 | 0.01 | 0.02 | 0.01 | 0.02 | 0.00 | 0.00 | 0.03 | 0.03 |
| M1 | 0.03 | 0.02 | 0.02 | 0.09 | 0.17 | 0.04 | 0.03 | 0.07 | 0.02 | 0.04 | 0.01 | 0.07 | 0.01 | 0.03 | 0.05 | 0.09 |
| A33 | 0.03 | 0.08 | 0.02 | 0.05 | 0.14 | 0.10 | 0.02 | 0.04 | 0.03 | 0.03 | 0.02 | 0.01 | 0.03 | 0.10 | 0.02 | 0.07 |
| A24a | 0.06 | 0.09 | 0.03 | 0.06 | 0.14 | 0.08 | 0.04 | 0.03 | 0.04 | 0.02 | 0.00 | 0.03 | 0.00 | 0.09 | 0.02 | 0.07 |
| A24b | 0.04 | 0.05 | 0.01 | 0.06 | 0.14 | 0.06 | 0.04 | 0.05 | 0.02 | 0.03 | 0.00 | 0.06 | -0.04 | 0.06 | 0.07 | 0.09 |
| STN | 0.06 | 0.08 | 0.07 | 0.05 | 0.16 | 0.08 | 0.05 | -0.01 | -0.01 | 0.00 | 0.02 | 0.04 | 0.00 | 0.06 | 0.04 | 0.06 |
| DLSd | 0.11 | 0.07 | 0.08 | 0.07 | 0.20 | 0.03 | 0.04 | 0.01 | 0.02 | 0.03 | 0.01 | 0.02 | 0.00 | 0.01 | 0.01 | 0.02 |
| DLSv | 0.11 | 0.07 | 0.07 | 0.06 | 0.16 | 0.03 | 0.05 | 0.04 | 0.04 | 0.04 | 0.04 | 0.05 | 0.01 | 0.02 | 0.04 | 0.02 |
| vOFC | 0.07 | 0.14 | 0.05 | 0.05 | 0.08 | 0.08 | 0.02 | 0.01 | 0.00 | 0.03 | 0.02 | 0.03 | 0.05 | 0.09 | 0.03 | 0.07 |
| l OFC | NaN | 0.13 | 0.04 | 0.05 | 0.06 | 0.10 | 0.04 | 0.00 | 0.00 | 0.02 | 0.01 | 0.01 | 0.00 | 0.05 | 0.01 | 0.04 |
| A Ins | 0.13 | NaN | 0.07 | 0.08 | 0.18 | 0.05 | 0.02 | 0.06 | 0.04 | 0.05 | 0.05 | 0.07 | 0.00 | 0.03 | 0.06 | 0.03 |
| NAcS | 0.04 | 0.07 | NaN | 0.01 | 0.06 | 0.06 | 0.03 | -0.02 | 0.01 | 0.00 | 0.04 | 0.04 | 0.02 | 0.05 | 0.03 | 0.04 |
| NacC | 0.05 | 0.08 | 0.01 | NaN | 0.09 | 0.03 | 0.03 | -0.01 | -0.01 | 0.03 | 0.04 | 0.03 | 0.04 | 0.05 | 0.01 | 0.03 |
| VMS | 0.06 | 0.18 | 0.06 | 0.09 | NaN | 0.12 | 0.04 | 0.06 | 0.05 | 0.07 | 0.07 | 0.11 | 0.06 | 0.15 | 0.06 | 0.10 |
| CEA | 0.10 | 0.05 | 0.06 | 0.03 | 0.12 | NaN | 0.02 | 0.02 | 0.03 | 0.02 | 0.05 | 0.06 | 0.02 | 0.03 | 0.03 | 0.03 |
| BLA | 0.04 | 0.02 | 0.03 | 0.03 | 0.04 | 0.02 | NaN | -0.02 | 0.00 | 0.00 | 0.01 | 0.01 | 0.02 | 0.06 | 0.01 | 0.02 |
| V1d | 0.00 | 0.06 | -0.02 | -0.01 | 0.06 | 0.02 | -0.02 | NaN | 0.04 | 0.02 | 0.07 | 0.04 | 0.10 | -0.01 | 0.01 | 0.05 |
| V1v | 0.00 | 0.04 | 0.01 | -0.01 | 0.05 | 0.03 | 0.00 | 0.04 | NaN | 0.00 | 0.05 | 0.04 | 0.11 | 0.00 | 0.02 | 0.11 |
| PPCx | 0.02 | 0.05 | 0.00 | 0.03 | 0.07 | 0.02 | 0.00 | 0.02 | 0.00 | NaN | 0.02 | 0.00 | -0.01 | 0.03 | 0.10 | 0.12 |
| DS | 0.01 | 0.05 | 0.04 | 0.04 | 0.07 | 0.05 | 0.01 | 0.07 | 0.05 | 0.02 | NaN | 0.01 | 0.02 | 0.08 | 0.01 | 0.08 |
| DG | 0.01 | 0.07 | 0.04 | 0.03 | 0.11 | 0.06 | 0.01 | 0.04 | 0.04 | 0.00 | 0.01 | NaN | 0.06 | 0.06 | 0.01 | 0.07 |
| CA1 | 0.00 | 0.00 | 0.02 | 0.04 | 0.06 | 0.02 | 0.02 | 0.10 | 0.11 | -0.01 | 0.02 | 0.06 | NaN | 0.01 | 0.03 | 0.10 |
| CA3 | 0.05 | 0.03 | 0.05 | 0.05 | 0.15 | 0.03 | 0.06 | -0.01 | 0.00 | 0.03 | 0.08 | 0.06 | 0.01 | NaN | 0.06 | 0.10 |
| A30c | 0.01 | 0.06 | 0.03 | 0.01 | 0.06 | 0.03 | 0.01 | 0.01 | 0.02 | 0.10 | 0.01 | 0.01 | 0.03 | 0.06 | NaN | 0.06 |
| A29c | 0.04 | 0.03 | 0.04 | 0.03 | 0.10 | 0.03 | 0.02 | 0.05 | 0.11 | 0.12 | 0.08 | 0.07 | 0.10 | 0.10 | 0.06 | NaN |

SEM Theta wPLI (500-2000ms), Wait Trials, electrodes 1:16

| **Electrodes** | **A32V** | **A32D** | **DMS** | **MDT** | **CMT** | **M2** | **LFC** | **ALM** | **M1** | **A33** | **A24a** | **A24b** | **STN** | **DLSd** | **DLSv** | **vOFC** |
| --- | --- | --- | --- | --- | --- | --- | --- | --- | --- | --- | --- | --- | --- | --- | --- | --- |
| A32V | NaN | 0.15 | 0.14 | 0.08 | 0.07 | 0.14 | 0.20 | 0.13 | 0.08 | 0.09 | 0.06 | 0.07 | 0.06 | 0.07 | 0.09 | 0.13 |
| A32D | 0.15 | NaN | 0.16 | 0.06 | 0.06 | 0.08 | 0.13 | 0.10 | 0.07 | 0.15 | 0.08 | 0.07 | 0.06 | 0.04 | 0.05 | 0.14 |
| DMS | 0.14 | 0.16 | NaN | 0.15 | 0.15 | 0.15 | 0.17 | 0.14 | 0.13 | 0.13 | 0.16 | 0.11 | 0.17 | 0.14 | 0.14 | 0.10 |
| MDT | 0.08 | 0.06 | 0.15 | NaN | 0.13 | 0.06 | 0.08 | 0.12 | 0.08 | 0.14 | 0.14 | 0.10 | 0.10 | 0.08 | 0.08 | 0.10 |
| CMT | 0.07 | 0.06 | 0.15 | 0.13 | NaN | 0.07 | 0.12 | 0.09 | 0.05 | 0.15 | 0.13 | 0.09 | 0.11 | 0.07 | 0.09 | 0.10 |
| M2 | 0.14 | 0.08 | 0.15 | 0.06 | 0.07 | NaN | 0.13 | 0.06 | 0.07 | 0.13 | 0.12 | 0.06 | 0.08 | 0.06 | 0.08 | 0.12 |
| LFC | 0.20 | 0.13 | 0.17 | 0.08 | 0.12 | 0.13 | NaN | 0.09 | 0.09 | 0.20 | 0.15 | 0.08 | 0.12 | 0.09 | 0.10 | 0.13 |
| ALM | 0.13 | 0.10 | 0.14 | 0.12 | 0.09 | 0.06 | 0.09 | NaN | 0.05 | 0.11 | 0.11 | 0.06 | 0.09 | 0.06 | 0.07 | 0.16 |
| M1 | 0.08 | 0.07 | 0.13 | 0.08 | 0.05 | 0.07 | 0.09 | 0.05 | NaN | 0.09 | 0.07 | 0.05 | 0.05 | 0.06 | 0.05 | 0.10 |
| A33 | 0.09 | 0.15 | 0.13 | 0.14 | 0.15 | 0.13 | 0.20 | 0.11 | 0.09 | NaN | 0.15 | 0.08 | 0.10 | 0.11 | 0.12 | 0.08 |
| A24a | 0.06 | 0.08 | 0.16 | 0.14 | 0.13 | 0.12 | 0.15 | 0.11 | 0.07 | 0.15 | NaN | 0.07 | 0.08 | 0.08 | 0.09 | 0.08 |
| A24b | 0.07 | 0.07 | 0.11 | 0.10 | 0.09 | 0.06 | 0.08 | 0.06 | 0.05 | 0.08 | 0.07 | NaN | 0.06 | 0.06 | 0.07 | 0.06 |
| STN | 0.06 | 0.06 | 0.17 | 0.10 | 0.11 | 0.08 | 0.12 | 0.09 | 0.05 | 0.10 | 0.08 | 0.06 | NaN | 0.07 | 0.11 | 0.09 |
| DLSd | 0.07 | 0.04 | 0.14 | 0.08 | 0.07 | 0.06 | 0.09 | 0.06 | 0.06 | 0.11 | 0.08 | 0.06 | 0.07 | NaN | 0.05 | 0.10 |
| DLSv | 0.09 | 0.05 | 0.14 | 0.08 | 0.09 | 0.08 | 0.10 | 0.07 | 0.05 | 0.12 | 0.09 | 0.07 | 0.11 | 0.05 | NaN | 0.12 |
| vOFC | 0.13 | 0.14 | 0.10 | 0.10 | 0.10 | 0.12 | 0.13 | 0.16 | 0.10 | 0.08 | 0.08 | 0.06 | 0.09 | 0.10 | 0.12 | NaN |
| l OFC | 0.13 | 0.16 | 0.06 | 0.14 | 0.18 | 0.19 | 0.21 | 0.13 | 0.12 | 0.12 | 0.08 | 0.06 | 0.10 | 0.13 | 0.12 | 0.09 |
| A Ins | 0.22 | 0.15 | 0.18 | 0.10 | 0.11 | 0.12 | 0.07 | 0.08 | 0.08 | 0.21 | 0.17 | 0.08 | 0.12 | 0.10 | 0.11 | 0.14 |
| NAcS | 0.09 | 0.09 | 0.06 | 0.11 | 0.12 | 0.10 | 0.09 | 0.10 | 0.04 | 0.11 | 0.06 | 0.06 | 0.10 | 0.09 | 0.11 | 0.08 |
| NacC | 0.12 | 0.12 | 0.07 | 0.10 | 0.08 | 0.09 | 0.08 | 0.10 | 0.08 | 0.11 | 0.09 | 0.07 | 0.11 | 0.10 | 0.12 | 0.09 |
| VMS | 0.21 | 0.23 | 0.10 | 0.15 | 0.18 | 0.17 | 0.21 | 0.18 | 0.14 | 0.18 | 0.18 | 0.12 | 0.18 | 0.16 | 0.19 | 0.14 |
| CEA | 0.14 | 0.10 | 0.14 | 0.08 | 0.11 | 0.06 | 0.08 | 0.07 | 0.06 | 0.12 | 0.10 | 0.06 | 0.13 | 0.07 | 0.07 | 0.12 |
| BLA | 0.10 | 0.10 | 0.09 | 0.09 | 0.08 | 0.05 | 0.05 | 0.09 | 0.06 | 0.09 | 0.07 | 0.06 | 0.14 | 0.08 | 0.08 | 0.06 |
| V1d | 0.04 | 0.06 | 0.07 | 0.07 | 0.05 | 0.07 | 0.09 | 0.07 | 0.07 | 0.08 | 0.07 | 0.08 | 0.05 | 0.09 | 0.07 | 0.06 |
| V1v | 0.04 | 0.07 | 0.08 | 0.14 | 0.08 | 0.08 | 0.08 | 0.08 | 0.08 | 0.09 | 0.09 | 0.12 | 0.11 | 0.10 | 0.09 | 0.06 |
| PPCx | 0.04 | 0.04 | 0.08 | 0.10 | 0.10 | 0.05 | 0.07 | 0.08 | 0.04 | 0.09 | 0.09 | 0.06 | 0.09 | 0.06 | 0.09 | 0.05 |
| DS | 0.05 | 0.07 | 0.10 | 0.10 | 0.12 | 0.08 | 0.09 | 0.06 | 0.07 | 0.08 | 0.09 | 0.07 | 0.10 | 0.09 | 0.08 | 0.05 |
| DG | 0.05 | 0.06 | 0.10 | 0.09 | 0.11 | 0.08 | 0.10 | 0.06 | 0.08 | 0.07 | 0.07 | 0.07 | 0.12 | 0.10 | 0.11 | 0.05 |
| CA1 | 0.07 | 0.05 | 0.09 | 0.11 | 0.10 | 0.06 | 0.04 | 0.06 | 0.06 | 0.07 | 0.07 | 0.10 | 0.11 | 0.08 | 0.05 | 0.06 |
| CA3 | 0.06 | 0.05 | 0.12 | 0.09 | 0.07 | 0.06 | 0.07 | 0.09 | 0.06 | 0.12 | 0.13 | 0.11 | 0.11 | 0.07 | 0.04 | 0.09 |
| A30c | 0.04 | 0.07 | 0.08 | 0.09 | 0.09 | 0.06 | 0.06 | 0.11 | 0.07 | 0.08 | 0.08 | 0.07 | 0.14 | 0.07 | 0.11 | 0.05 |
| A29c | 0.06 | 0.06 | 0.12 | 0.12 | 0.10 | 0.06 | 0.05 | 0.10 | 0.10 | 0.09 | 0.10 | 0.10 | 0.07 | 0.07 | 0.08 | 0.06 |

SEM Theta wPLI (500-2000ms), Wait Trials, electrodes 17:32

|  | **l OFC** | **A Ins** | **NAcS** | **NacC** | **VMS** | **CEA** | **BLA** | **V1d** | **V1v** | **PPCx** | **DS** | **DG** | **CA1** | **CA3** | **A30c** | **A29c** |
| --- | --- | --- | --- | --- | --- | --- | --- | --- | --- | --- | --- | --- | --- | --- | --- | --- |
| A32V | 0.13 | 0.22 | 0.09 | 0.12 | 0.21 | 0.14 | 0.10 | 0.04 | 0.04 | 0.04 | 0.05 | 0.05 | 0.07 | 0.06 | 0.04 | 0.06 |
| A32D | 0.16 | 0.15 | 0.09 | 0.12 | 0.23 | 0.10 | 0.10 | 0.06 | 0.07 | 0.04 | 0.07 | 0.06 | 0.05 | 0.05 | 0.07 | 0.06 |
| DMS | 0.06 | 0.18 | 0.06 | 0.07 | 0.10 | 0.14 | 0.09 | 0.07 | 0.08 | 0.08 | 0.10 | 0.10 | 0.09 | 0.12 | 0.08 | 0.12 |
| MDT | 0.14 | 0.10 | 0.11 | 0.10 | 0.15 | 0.08 | 0.09 | 0.07 | 0.14 | 0.10 | 0.10 | 0.09 | 0.11 | 0.09 | 0.09 | 0.12 |
| CMT | 0.18 | 0.11 | 0.12 | 0.08 | 0.18 | 0.11 | 0.08 | 0.05 | 0.08 | 0.10 | 0.12 | 0.11 | 0.10 | 0.07 | 0.09 | 0.10 |
| M2 | 0.19 | 0.12 | 0.10 | 0.09 | 0.17 | 0.06 | 0.05 | 0.07 | 0.08 | 0.05 | 0.08 | 0.08 | 0.06 | 0.06 | 0.06 | 0.06 |
| LFC | 0.21 | 0.07 | 0.09 | 0.08 | 0.21 | 0.08 | 0.05 | 0.09 | 0.08 | 0.07 | 0.09 | 0.10 | 0.04 | 0.07 | 0.06 | 0.05 |
| ALM | 0.13 | 0.08 | 0.10 | 0.10 | 0.18 | 0.07 | 0.09 | 0.07 | 0.08 | 0.08 | 0.06 | 0.06 | 0.06 | 0.09 | 0.11 | 0.10 |
| M1 | 0.12 | 0.08 | 0.04 | 0.08 | 0.14 | 0.06 | 0.06 | 0.07 | 0.08 | 0.04 | 0.07 | 0.08 | 0.06 | 0.06 | 0.07 | 0.10 |
| A33 | 0.12 | 0.21 | 0.11 | 0.11 | 0.18 | 0.12 | 0.09 | 0.08 | 0.09 | 0.09 | 0.08 | 0.07 | 0.07 | 0.12 | 0.08 | 0.09 |
| A24a | 0.08 | 0.17 | 0.06 | 0.09 | 0.18 | 0.10 | 0.07 | 0.07 | 0.09 | 0.09 | 0.09 | 0.07 | 0.07 | 0.13 | 0.08 | 0.10 |
| A24b | 0.06 | 0.08 | 0.06 | 0.07 | 0.12 | 0.06 | 0.06 | 0.08 | 0.12 | 0.06 | 0.07 | 0.07 | 0.10 | 0.11 | 0.07 | 0.10 |
| STN | 0.10 | 0.12 | 0.10 | 0.11 | 0.18 | 0.13 | 0.14 | 0.05 | 0.11 | 0.09 | 0.10 | 0.12 | 0.11 | 0.11 | 0.14 | 0.07 |
| DLSd | 0.13 | 0.10 | 0.09 | 0.10 | 0.16 | 0.07 | 0.08 | 0.09 | 0.10 | 0.06 | 0.09 | 0.10 | 0.08 | 0.07 | 0.07 | 0.07 |
| DLSv | 0.12 | 0.11 | 0.11 | 0.12 | 0.19 | 0.07 | 0.08 | 0.07 | 0.09 | 0.09 | 0.08 | 0.11 | 0.05 | 0.04 | 0.11 | 0.08 |
| vOFC | 0.09 | 0.14 | 0.08 | 0.09 | 0.14 | 0.12 | 0.06 | 0.06 | 0.06 | 0.05 | 0.05 | 0.05 | 0.06 | 0.09 | 0.05 | 0.06 |
| l OFC | NaN | 0.20 | 0.12 | 0.11 | 0.15 | 0.13 | 0.07 | 0.06 | 0.05 | 0.07 | 0.09 | 0.07 | 0.06 | 0.08 | 0.06 | 0.08 |
| A Ins | 0.20 | NaN | 0.08 | 0.09 | 0.22 | 0.08 | 0.06 | 0.10 | 0.08 | 0.08 | 0.08 | 0.09 | 0.04 | 0.05 | 0.07 | 0.06 |
| NAcS | 0.12 | 0.08 | NaN | 0.08 | 0.13 | 0.09 | 0.12 | 0.07 | 0.08 | 0.08 | 0.11 | 0.08 | 0.06 | 0.07 | 0.08 | 0.07 |
| NacC | 0.11 | 0.09 | 0.08 | NaN | 0.10 | 0.12 | 0.10 | 0.11 | 0.09 | 0.06 | 0.06 | 0.06 | 0.09 | 0.08 | 0.08 | 0.07 |
| VMS | 0.15 | 0.22 | 0.13 | 0.10 | NaN | 0.18 | 0.10 | 0.07 | 0.07 | 0.06 | 0.13 | 0.13 | 0.07 | 0.10 | 0.06 | 0.09 |
| CEA | 0.13 | 0.08 | 0.09 | 0.12 | 0.18 | NaN | 0.07 | 0.07 | 0.08 | 0.08 | 0.09 | 0.13 | 0.06 | 0.05 | 0.07 | 0.07 |
| BLA | 0.07 | 0.06 | 0.12 | 0.10 | 0.10 | 0.07 | NaN | 0.06 | 0.06 | 0.08 | 0.06 | 0.07 | 0.06 | 0.11 | 0.07 | 0.06 |
| V1d | 0.06 | 0.10 | 0.07 | 0.11 | 0.07 | 0.07 | 0.06 | NaN | 0.09 | 0.09 | 0.11 | 0.10 | 0.10 | 0.06 | 0.10 | 0.10 |
| V1v | 0.05 | 0.08 | 0.08 | 0.09 | 0.07 | 0.08 | 0.06 | 0.09 | NaN | 0.11 | 0.11 | 0.10 | 0.11 | 0.10 | 0.10 | 0.14 |
| PPCx | 0.07 | 0.08 | 0.08 | 0.06 | 0.06 | 0.08 | 0.08 | 0.09 | 0.11 | NaN | 0.06 | 0.04 | 0.13 | 0.16 | 0.12 | 0.12 |
| DS | 0.09 | 0.08 | 0.11 | 0.06 | 0.13 | 0.09 | 0.06 | 0.11 | 0.11 | 0.06 | NaN | 0.08 | 0.15 | 0.12 | 0.08 | 0.11 |
| DG | 0.07 | 0.09 | 0.08 | 0.06 | 0.13 | 0.13 | 0.07 | 0.10 | 0.10 | 0.04 | 0.08 | NaN | 0.13 | 0.11 | 0.06 | 0.13 |
| CA1 | 0.06 | 0.04 | 0.06 | 0.09 | 0.07 | 0.06 | 0.06 | 0.10 | 0.11 | 0.13 | 0.15 | 0.13 | NaN | 0.10 | 0.12 | 0.23 |
| CA3 | 0.08 | 0.05 | 0.07 | 0.08 | 0.10 | 0.05 | 0.11 | 0.06 | 0.10 | 0.16 | 0.12 | 0.11 | 0.10 | NaN | 0.13 | 0.14 |
| A30c | 0.06 | 0.07 | 0.08 | 0.08 | 0.06 | 0.07 | 0.07 | 0.10 | 0.10 | 0.12 | 0.08 | 0.06 | 0.12 | 0.13 | NaN | 0.11 |
| A29c | 0.08 | 0.06 | 0.07 | 0.07 | 0.09 | 0.07 | 0.06 | 0.10 | 0.14 | 0.12 | 0.11 | 0.13 | 0.23 | 0.14 | 0.11 | NaN |

Adj. p-values Theta wPLI (500-2000ms), Wait Trials, electrodes 1:16

| **Electrodes** | **A32V** | **A32D** | **DMS** | **MDT** | **CMT** | **M2** | **LFC** | **ALM** | **M1** | **A33** | **A24a** | **A24b** | **STN** | **DLSd** | **DLSv** | **vOFC** |
| --- | --- | --- | --- | --- | --- | --- | --- | --- | --- | --- | --- | --- | --- | --- | --- | --- |
| A32V | NaN | 0.00 | 0.10 | 0.20 | 1.00 | 0.00 | 0.00 | 0.00 | 0.00 | 0.24 | 1.00 | 0.05 | 0.08 | 0.00 | 0.03 | 0.01 |
| A32D | 0.00 | NaN | 0.03 | 1.00 | 1.00 | 0.00 | 0.00 | 1.00 | 1.00 | 0.08 | 0.94 | 1.00 | 1.00 | 1.00 | 1.00 | 0.01 |
| DMS | 0.10 | 0.03 | NaN | 0.18 | 0.00 | 0.00 | 0.00 | 0.02 | 0.00 | 0.01 | 0.10 | 0.00 | 0.02 | 0.00 | 0.01 | 1.00 |
| MDT | 0.20 | 1.00 | 0.18 | NaN | 1.00 | 1.00 | 0.01 | 1.00 | 1.00 | 1.00 | 0.26 | 1.00 | 1.00 | 1.00 | 1.00 | 0.02 |
| CMT | 1.00 | 1.00 | 0.00 | 1.00 | NaN | 0.00 | 0.25 | 1.00 | 1.00 | 0.22 | 0.09 | 0.02 | 0.68 | 1.00 | 1.00 | 0.00 |
| M2 | 0.00 | 0.00 | 0.00 | 1.00 | 0.00 | NaN | 0.05 | 0.00 | 1.00 | 0.00 | 0.00 | 0.02 | 0.00 | 0.00 | 0.04 | 0.00 |
| LFC | 0.00 | 0.00 | 0.00 | 0.01 | 0.25 | 0.05 | NaN | 0.00 | 1.00 | 0.06 | 0.00 | 0.00 | 0.00 | 0.00 | 0.00 | 0.00 |
| ALM | 0.00 | 1.00 | 0.02 | 1.00 | 1.00 | 0.00 | 0.00 | NaN | 0.42 | 0.01 | 0.86 | 1.00 | 0.06 | 1.00 | 1.00 | 1.00 |
| M1 | 0.00 | 1.00 | 0.00 | 1.00 | 1.00 | 1.00 | 1.00 | 0.42 | NaN | 0.00 | 0.00 | 0.33 | 0.00 | 1.00 | 1.00 | 0.00 |
| A33 | 0.24 | 0.08 | 0.01 | 1.00 | 0.22 | 0.00 | 0.06 | 0.01 | 0.00 | NaN | 1.00 | 0.02 | 0.00 | 0.00 | 0.00 | 0.00 |
| A24a | 1.00 | 0.94 | 0.10 | 0.26 | 0.09 | 0.00 | 0.00 | 0.86 | 0.00 | 1.00 | NaN | 0.00 | 1.00 | 0.00 | 0.00 | 0.00 |
| A24b | 0.05 | 1.00 | 0.00 | 1.00 | 0.02 | 0.02 | 0.00 | 1.00 | 0.33 | 0.02 | 0.00 | NaN | 0.00 | 1.00 | 1.00 | 0.00 |
| STN | 0.08 | 1.00 | 0.02 | 1.00 | 0.68 | 0.00 | 0.00 | 0.06 | 0.00 | 0.00 | 1.00 | 0.00 | NaN | 1.00 | 0.00 | 0.00 |
| DLSd | 0.00 | 1.00 | 0.00 | 1.00 | 1.00 | 0.00 | 0.00 | 1.00 | 1.00 | 0.00 | 0.00 | 1.00 | 1.00 | NaN | 1.00 | 0.00 |
| DLSv | 0.03 | 1.00 | 0.01 | 1.00 | 1.00 | 0.04 | 0.00 | 1.00 | 1.00 | 0.00 | 0.00 | 1.00 | 0.00 | 1.00 | NaN | 0.00 |
| vOFC | 0.01 | 0.01 | 1.00 | 0.02 | 0.00 | 0.00 | 0.00 | 1.00 | 0.00 | 0.00 | 0.00 | 0.00 | 0.00 | 0.00 | 0.00 | NaN |
| l OFC | 0.00 | 0.00 | 1.00 | 0.05 | 0.04 | 0.00 | 0.01 | 0.00 | 1.00 | 1.00 | 0.00 | 0.00 | 0.00 | 0.00 | 0.00 | 0.00 |
| A Ins | 0.00 | 0.07 | 0.03 | 0.88 | 0.01 | 0.00 | 1.00 | 0.00 | 1.00 | 1.00 | 0.21 | 0.00 | 0.00 | 0.00 | 0.01 | 0.00 |
| NAcS | 1.00 | 1.00 | 1.00 | 0.96 | 1.00 | 1.00 | 0.00 | 1.00 | 0.52 | 1.00 | 0.03 | 1.00 | 0.00 | 0.00 | 0.01 | 0.01 |
| NacC | 0.40 | 1.00 | 0.00 | 1.00 | 1.00 | 1.00 | 0.00 | 1.00 | 0.00 | 0.25 | 0.00 | 0.00 | 0.41 | 0.00 | 0.36 | 0.12 |
| VMS | 0.01 | 0.01 | 0.00 | 0.00 | 0.00 | 0.00 | 0.00 | 0.01 | 0.00 | 0.00 | 0.00 | 0.00 | 0.00 | 0.00 | 0.00 | 0.01 |
| CEA | 0.59 | 1.00 | 0.01 | 1.00 | 1.00 | 0.00 | 0.83 | 0.00 | 0.00 | 0.00 | 0.00 | 0.00 | 0.00 | 0.96 | 0.92 | 0.00 |
| BLA | 1.00 | 1.00 | 0.70 | 1.00 | 1.00 | 0.00 | 1.00 | 1.00 | 0.06 | 1.00 | 0.05 | 0.00 | 1.00 | 0.03 | 0.00 | 1.00 |
| V1d | 1.00 | 0.00 | 0.00 | 1.00 | 1.00 | 0.95 | 0.00 | 1.00 | 0.00 | 0.18 | 0.45 | 0.01 | 1.00 | 1.00 | 0.05 | 1.00 |
| V1v | 1.00 | 0.93 | 0.00 | 1.00 | 1.00 | 1.00 | 0.05 | 1.00 | 1.00 | 1.00 | 1.00 | 1.00 | 1.00 | 1.00 | 1.00 | 1.00 |
| PPCx | 1.00 | 1.00 | 0.00 | 1.00 | 1.00 | 0.00 | 0.00 | 1.00 | 0.00 | 1.00 | 1.00 | 1.00 | 1.00 | 0.10 | 0.28 | 0.00 |
| DS | 1.00 | 1.00 | 0.00 | 1.00 | 1.00 | 0.00 | 0.00 | 1.00 | 1.00 | 1.00 | 1.00 | 1.00 | 1.00 | 1.00 | 1.00 | 1.00 |
| DG | 1.00 | 0.07 | 0.00 | 1.00 | 0.01 | 0.00 | 0.00 | 1.00 | 0.00 | 1.00 | 0.15 | 0.00 | 1.00 | 1.00 | 1.00 | 0.00 |
| CA1 | 1.00 | 1.00 | 0.00 | 0.00 | 1.00 | 1.00 | 1.00 | 1.00 | 1.00 | 1.00 | 1.00 | 1.00 | 1.00 | 1.00 | 1.00 | 0.00 |
| CA3 | 1.00 | 1.00 | 0.00 | 1.00 | 0.00 | 1.00 | 0.00 | 1.00 | 0.07 | 0.00 | 0.00 | 0.03 | 0.03 | 1.00 | 0.02 | 0.00 |
| A30c | 1.00 | 1.00 | 0.12 | 1.00 | 1.00 | 1.00 | 0.00 | 1.00 | 0.00 | 1.00 | 1.00 | 0.00 | 1.00 | 1.00 | 1.00 | 0.00 |
| A29c | 0.67 | 0.19 | 0.00 | 1.00 | 1.00 | 0.27 | 0.00 | 1.00 | 0.00 | 0.00 | 0.00 | 0.00 | 0.00 | 1.00 | 1.00 | 0.00 |

Adj. p-values Theta wPLI (500-2000ms), Wait Trials, electrodes 17:32

|  | l OFC | A Ins | NAcS | NacC | VMS | CEA | BLA | V1d | V1v | PPCx | DS | DG | CA1 | CA3 | A30c | A29c |
| --- | --- | --- | --- | --- | --- | --- | --- | --- | --- | --- | --- | --- | --- | --- | --- | --- |
| A32V | 0.00 | 0.00 | 1.00 | 0.40 | 0.01 | 0.59 | 1.00 | 1.00 | 1.00 | 1.00 | 1.00 | 1.00 | 1.00 | 1.00 | 1.00 | 0.67 |
| A32D | 0.00 | 0.07 | 1.00 | 1.00 | 0.01 | 1.00 | 1.00 | 0.00 | 0.93 | 1.00 | 1.00 | 0.07 | 1.00 | 1.00 | 1.00 | 0.19 |
| DMS | 1.00 | 0.03 | 1.00 | 0.00 | 0.00 | 0.01 | 0.70 | 0.00 | 0.00 | 0.00 | 0.00 | 0.00 | 0.00 | 0.00 | 0.12 | 0.00 |
| MDT | 0.05 | 0.88 | 0.96 | 1.00 | 0.00 | 1.00 | 1.00 | 1.00 | 1.00 | 1.00 | 1.00 | 1.00 | 0.00 | 1.00 | 1.00 | 1.00 |
| CMT | 0.04 | 0.01 | 1.00 | 1.00 | 0.00 | 1.00 | 1.00 | 1.00 | 1.00 | 1.00 | 1.00 | 0.01 | 1.00 | 0.00 | 1.00 | 1.00 |
| M2 | 0.00 | 0.00 | 1.00 | 1.00 | 0.00 | 0.00 | 0.00 | 0.95 | 1.00 | 0.00 | 0.00 | 0.00 | 1.00 | 1.00 | 1.00 | 0.27 |
| LFC | 0.01 | 1.00 | 0.00 | 0.00 | 0.00 | 0.83 | 1.00 | 0.00 | 0.05 | 0.00 | 0.00 | 0.00 | 1.00 | 0.00 | 0.00 | 0.00 |
| ALM | 0.00 | 0.00 | 1.00 | 1.00 | 0.01 | 0.00 | 1.00 | 1.00 | 1.00 | 1.00 | 1.00 | 1.00 | 1.00 | 1.00 | 1.00 | 1.00 |
| M1 | 1.00 | 1.00 | 0.52 | 0.00 | 0.00 | 0.00 | 0.06 | 0.00 | 1.00 | 0.00 | 1.00 | 0.00 | 1.00 | 0.07 | 0.00 | 0.00 |
| A33 | 1.00 | 1.00 | 1.00 | 0.25 | 0.00 | 0.00 | 1.00 | 0.18 | 1.00 | 1.00 | 1.00 | 1.00 | 1.00 | 0.00 | 1.00 | 0.00 |
| A24a | 0.00 | 0.21 | 0.03 | 0.00 | 0.00 | 0.00 | 0.05 | 0.45 | 1.00 | 1.00 | 1.00 | 0.15 | 1.00 | 0.00 | 1.00 | 0.00 |
| A24b | 0.00 | 0.00 | 1.00 | 0.00 | 0.00 | 0.00 | 0.00 | 0.01 | 1.00 | 1.00 | 1.00 | 0.00 | 1.00 | 0.03 | 0.00 | 0.00 |
| STN | 0.00 | 0.00 | 0.00 | 0.41 | 0.00 | 0.00 | 1.00 | 1.00 | 1.00 | 1.00 | 1.00 | 1.00 | 1.00 | 0.03 | 1.00 | 0.00 |
| DLSd | 0.00 | 0.00 | 0.00 | 0.00 | 0.00 | 0.96 | 0.03 | 1.00 | 1.00 | 0.10 | 1.00 | 1.00 | 1.00 | 1.00 | 1.00 | 1.00 |
| DLSv | 0.00 | 0.01 | 0.01 | 0.36 | 0.00 | 0.92 | 0.00 | 0.05 | 1.00 | 0.28 | 1.00 | 1.00 | 1.00 | 0.02 | 1.00 | 1.00 |
| vOFC | 0.00 | 0.00 | 0.01 | 0.12 | 0.01 | 0.00 | 1.00 | 1.00 | 1.00 | 0.00 | 1.00 | 0.00 | 0.00 | 0.00 | 0.00 | 0.00 |
| l OFC | NaN | 0.00 | 1.00 | 1.00 | 1.00 | 0.00 | 0.04 | 1.00 | 1.00 | 1.00 | 1.00 | 1.00 | 1.00 | 0.01 | 1.00 | 0.48 |
| A Ins | 0.00 | NaN | 0.00 | 0.00 | 0.00 | 0.01 | 1.00 | 0.01 | 0.62 | 0.00 | 0.01 | 0.00 | 1.00 | 0.01 | 0.00 | 0.09 |
| NAcS | 1.00 | 0.00 | NaN | 1.00 | 0.61 | 0.00 | 1.00 | 1.00 | 1.00 | 1.00 | 1.00 | 0.21 | 1.00 | 0.00 | 1.00 | 0.01 |
| NacC | 1.00 | 0.00 | 1.00 | NaN | 0.00 | 1.00 | 1.00 | 1.00 | 1.00 | 0.50 | 0.01 | 0.01 | 1.00 | 0.00 | 1.00 | 0.14 |
| VMS | 1.00 | 0.00 | 0.61 | 0.00 | NaN | 0.00 | 1.00 | 0.00 | 0.00 | 0.00 | 0.11 | 0.00 | 0.00 | 0.00 | 0.00 | 0.00 |
| CEA | 0.00 | 0.01 | 0.00 | 1.00 | 0.00 | NaN | 1.00 | 1.00 | 1.00 | 1.00 | 0.04 | 0.17 | 1.00 | 0.01 | 1.00 | 1.00 |
| BLA | 0.04 | 1.00 | 1.00 | 1.00 | 1.00 | 1.00 | NaN | 1.00 | 1.00 | 1.00 | 1.00 | 1.00 | 1.00 | 0.25 | 1.00 | 1.00 |
| V1d | 1.00 | 0.01 | 1.00 | 1.00 | 0.00 | 1.00 | 1.00 | NaN | 0.57 | 1.00 | 0.01 | 1.00 | 0.00 | 1.00 | 1.00 | 0.10 |
| V1v | 1.00 | 0.62 | 1.00 | 1.00 | 0.00 | 1.00 | 1.00 | 0.57 | NaN | 1.00 | 0.34 | 1.00 | 0.00 | 1.00 | 1.00 | 0.00 |
| PPCx | 1.00 | 0.00 | 1.00 | 0.50 | 0.00 | 1.00 | 1.00 | 1.00 | 1.00 | NaN | 1.00 | 1.00 | 1.00 | 1.00 | 0.00 | 0.00 |
| DS | 1.00 | 0.01 | 1.00 | 0.01 | 0.11 | 0.04 | 1.00 | 0.01 | 0.34 | 1.00 | NaN | 1.00 | 1.00 | 0.00 | 1.00 | 0.00 |
| DG | 1.00 | 0.00 | 0.21 | 0.01 | 0.00 | 0.17 | 1.00 | 1.00 | 1.00 | 1.00 | 1.00 | NaN | 1.00 | 0.06 | 1.00 | 0.16 |
| CA1 | 1.00 | 1.00 | 1.00 | 1.00 | 0.00 | 1.00 | 1.00 | 0.00 | 0.00 | 1.00 | 1.00 | 1.00 | NaN | 1.00 | 1.00 | 0.84 |
| CA3 | 0.01 | 0.01 | 0.00 | 0.00 | 0.00 | 0.01 | 0.25 | 1.00 | 1.00 | 1.00 | 0.00 | 0.06 | 1.00 | NaN | 0.21 | 0.00 |
| A30c | 1.00 | 0.00 | 1.00 | 1.00 | 0.00 | 1.00 | 1.00 | 1.00 | 1.00 | 0.00 | 1.00 | 1.00 | 1.00 | 0.21 | NaN | 0.13 |
| A29c | 0.48 | 0.09 | 0.01 | 0.14 | 0.00 | 1.00 | 1.00 | 0.10 | 0.00 | 0.00 | 0.00 | 0.16 | 0.84 | 0.00 | 0.13 | NaN |
|  |  |  |  |  |  |  |  |  |  |  |  |  |  |  |  |  |

**Figure 7_1:** Data table for mean, SEM and p-values for pair-wise wPLI. Data taken at theta-frequencies between 500-2000ms post-stimulus. P-values adjusted for multiple comparisons using FDR-correction
