# Supplementary material for: Mapping Large-Scale Networks Associated with Action, Behavioral Inhibition and Impulsivity
Source: eNeuro. 2021 Feb 23;8(1):ENEURO.0406-20.2021. doi: 10.1523/ENEURO.0406-20.2021 (PMC7920541; doi:10.1523/ENEURO.0406-20.2021)
Supplement: Extended Data Figure 7-2 — Data table for mean, SEM and p-values for pair-wise wPLI for the [wait-go] contrast. Statistical test used was a one-sample t-test, mean Data averaged at theta-frequencies between 500-2000ms post-stimulus. P-values adjusted for multiple comparisons using FDR-correction. Download Figure 7-2, DOCX file. [file enu-eN-NWR-0406-20-s07.docx]

**Theta wPLI for [Wait – Go Contrast]**

Mean Theta wPLI (500-2000ms), [Wait – Go], electrodes 1:16

|  | **A32V** | **A32D** | **DMS** | **MDT** | **CMT** | **M2** | **LFC** | **ALM** | **M1** | **A33** | **A24a** | **A24b** | **STN** | **DLSd** | **DLSv** | **vOFC** |
| --- | --- | --- | --- | --- | --- | --- | --- | --- | --- | --- | --- | --- | --- | --- | --- | --- |
| A32V | NaN | 0.153 | 0.096 | 0.020 | 0.003 | 0.218 | 0.232 | 0.141 | 0.070 | 0.041 | 0.039 | 0.081 | 0.066 | 0.090 | 0.137 | 0.098 |
| A32D | 0.153 | NaN | 0.105 | -0.029 | -0.016 | 0.090 | 0.093 | 0.010 | 0.059 | 0.114 | 0.046 | 0.022 | 0.020 | -0.010 | 0.013 | 0.160 |
| DMS | 0.096 | 0.105 | NaN | 0.156 | 0.207 | 0.137 | 0.147 | 0.119 | 0.227 | 0.124 | 0.140 | 0.160 | 0.153 | 0.166 | 0.150 | 0.057 |
| MDT | 0.020 | -0.029 | 0.156 | NaN | -0.025 | -0.012 | 0.039 | -0.001 | 0.007 | 0.059 | 0.071 | 0.020 | 0.016 | 0.003 | 0.027 | 0.060 |
| CMT | 0.003 | -0.016 | 0.207 | -0.025 | NaN | 0.040 | 0.071 | 0.032 | 0.046 | 0.114 | 0.088 | 0.026 | 0.011 | 0.007 | 0.062 | 0.071 |
| M2 | 0.218 | 0.090 | 0.137 | -0.012 | 0.040 | NaN | 0.036 | 0.040 | -0.024 | 0.208 | 0.180 | 0.011 | 0.055 | -0.002 | -0.019 | 0.161 |
| LFC | 0.232 | 0.093 | 0.147 | 0.039 | 0.071 | 0.036 | NaN | 0.094 | -0.001 | 0.142 | 0.117 | 0.050 | 0.101 | 0.050 | 0.030 | 0.202 |
| ALM | 0.141 | 0.010 | 0.119 | -0.001 | 0.032 | 0.040 | 0.094 | NaN | 0.016 | 0.100 | 0.064 | 0.010 | 0.055 | -0.006 | 0.019 | 0.109 |
| M1 | 0.070 | 0.059 | 0.227 | 0.007 | 0.046 | -0.024 | -0.001 | 0.016 | NaN | 0.139 | 0.089 | 0.022 | 0.049 | -0.006 | 0.030 | 0.123 |
| A33 | 0.041 | 0.114 | 0.124 | 0.059 | 0.114 | 0.208 | 0.142 | 0.100 | 0.139 | NaN | -0.001 | 0.067 | 0.086 | 0.108 | 0.117 | 0.076 |
| A24a | 0.039 | 0.046 | 0.140 | 0.071 | 0.088 | 0.180 | 0.117 | 0.064 | 0.089 | -0.001 | NaN | 0.072 | 0.051 | 0.084 | 0.115 | 0.137 |
| A24b | 0.081 | 0.022 | 0.160 | 0.020 | 0.026 | 0.011 | 0.050 | 0.010 | 0.022 | 0.067 | 0.072 | NaN | 0.035 | 0.014 | 0.038 | 0.144 |
| STN | 0.066 | 0.020 | 0.153 | 0.016 | 0.011 | 0.055 | 0.101 | 0.055 | 0.049 | 0.086 | 0.051 | 0.035 | NaN | 0.088 | 0.169 | 0.091 |
| DLSd | 0.090 | -0.010 | 0.166 | 0.003 | 0.007 | -0.002 | 0.050 | -0.006 | -0.006 | 0.108 | 0.084 | 0.014 | 0.088 | NaN | 0.023 | 0.113 |
| DLSv | 0.137 | 0.013 | 0.150 | 0.027 | 0.062 | -0.019 | 0.030 | 0.019 | 0.030 | 0.117 | 0.115 | 0.038 | 0.169 | 0.023 | NaN | 0.133 |
| vOFC | 0.098 | 0.160 | 0.057 | 0.060 | 0.071 | 0.161 | 0.202 | 0.109 | 0.123 | 0.076 | 0.137 | 0.144 | 0.091 | 0.113 | 0.133 | NaN |
| l OFC | 0.124 | 0.222 | 0.005 | 0.101 | 0.151 | 0.167 | 0.127 | 0.104 | 0.032 | 0.052 | 0.080 | 0.035 | 0.094 | 0.119 | 0.109 | 0.074 |
| A Ins | 0.199 | 0.083 | 0.102 | 0.051 | 0.083 | 0.057 | -0.010 | 0.089 | -0.003 | 0.116 | 0.104 | 0.021 | 0.114 | 0.054 | 0.008 | 0.189 |
| NAcS | 0.050 | 0.036 | 0.025 | 0.020 | 0.019 | 0.000 | 0.059 | 0.031 | 0.002 | 0.039 | 0.036 | -0.009 | 0.054 | 0.068 | 0.060 | 0.024 |
| NacC | 0.152 | 0.162 | 0.086 | 0.049 | 0.047 | 0.100 | 0.147 | 0.043 | 0.133 | 0.127 | 0.125 | 0.104 | 0.075 | 0.132 | 0.131 | 0.067 |
| VMS | 0.191 | 0.262 | 0.126 | 0.143 | 0.201 | 0.169 | 0.270 | 0.155 | 0.235 | 0.221 | 0.231 | 0.227 | 0.209 | 0.259 | 0.212 | 0.098 |
| CEA | 0.160 | 0.060 | 0.154 | 0.041 | 0.060 | -0.019 | 0.008 | 0.033 | 0.061 | 0.154 | 0.133 | 0.069 | 0.136 | 0.003 | 0.024 | 0.142 |
| BLA | 0.040 | 0.020 | 0.078 | 0.021 | -0.014 | 0.003 | -0.029 | 0.018 | 0.040 | 0.032 | 0.058 | 0.041 | 0.074 | 0.022 | 0.034 | 0.044 |
| V1d | 0.034 | 0.078 | 0.114 | -0.016 | 0.006 | 0.080 | 0.090 | 0.030 | 0.146 | 0.087 | 0.087 | 0.115 | -0.015 | -0.026 | 0.021 | 0.049 |
| V1v | 0.002 | 0.046 | 0.103 | -0.022 | 0.013 | 0.077 | 0.068 | 0.056 | 0.130 | 0.080 | 0.083 | 0.079 | -0.015 | -0.003 | 0.022 | 0.026 |
| PPCx | 0.002 | 0.053 | 0.123 | 0.024 | 0.059 | 0.100 | 0.113 | 0.062 | 0.152 | 0.101 | 0.092 | 0.101 | 0.033 | 0.014 | 0.042 | 0.038 |
| DS | 0.024 | 0.031 | 0.084 | 0.009 | 0.040 | 0.080 | 0.076 | 0.054 | 0.069 | 0.027 | 0.014 | 0.031 | -0.007 | -0.026 | 0.019 | 0.035 |
| DG | 0.034 | 0.013 | 0.079 | 0.064 | 0.122 | 0.077 | 0.071 | 0.040 | 0.127 | 0.018 | 0.041 | 0.064 | 0.005 | 0.023 | 0.035 | 0.071 |
| CA1 | -0.012 | 0.002 | 0.094 | -0.062 | -0.030 | -0.003 | 0.006 | 0.000 | 0.027 | 0.020 | -0.016 | -0.027 | -0.026 | -0.050 | -0.022 | 0.056 |
| CA3 | 0.019 | 0.014 | 0.151 | 0.029 | 0.072 | 0.019 | 0.042 | 0.019 | 0.064 | 0.119 | 0.098 | 0.083 | 0.088 | 0.024 | 0.047 | 0.082 |
| A30c | -0.001 | 0.043 | 0.094 | 0.039 | 0.055 | 0.095 | 0.087 | 0.052 | 0.153 | 0.078 | 0.095 | 0.137 | 0.087 | -0.007 | 0.045 | 0.029 |
| A29c | 0.017 | 0.025 | 0.148 | -0.011 | 0.009 | 0.046 | 0.044 | 0.028 | 0.102 | 0.064 | 0.061 | 0.081 | 0.052 | 0.004 | 0.025 | 0.069 |

Mean Theta wPLI (500-2000ms), [Wait – Go], electrodes 17:32

|  | **l OFC** | **A Ins** | **NAcS** | **NacC** | **VMS** | **CEA** | **BLA** | **V1d** | **V1v** | **PPCx** | **DS** | **DG** | **CA1** | **CA3** | **A30c** | **A29c** |
| --- | --- | --- | --- | --- | --- | --- | --- | --- | --- | --- | --- | --- | --- | --- | --- | --- |
| A32V | 0.124 | 0.199 | 0.050 | 0.152 | 0.191 | 0.160 | 0.040 | 0.034 | 0.002 | 0.002 | 0.024 | 0.034 | -0.012 | 0.019 | -0.001 | 0.017 |
| A32D | 0.222 | 0.083 | 0.036 | 0.162 | 0.262 | 0.060 | 0.020 | 0.078 | 0.046 | 0.053 | 0.031 | 0.013 | 0.002 | 0.014 | 0.043 | 0.025 |
| DMS | 0.005 | 0.102 | 0.025 | 0.086 | 0.126 | 0.154 | 0.078 | 0.114 | 0.103 | 0.123 | 0.084 | 0.079 | 0.094 | 0.151 | 0.094 | 0.148 |
| MDT | 0.101 | 0.051 | 0.020 | 0.049 | 0.143 | 0.041 | 0.021 | -0.016 | -0.022 | 0.024 | 0.009 | 0.064 | -0.062 | 0.029 | 0.039 | -0.011 |
| CMT | 0.151 | 0.083 | 0.019 | 0.047 | 0.201 | 0.060 | -0.014 | 0.006 | 0.013 | 0.059 | 0.040 | 0.122 | -0.030 | 0.072 | 0.055 | 0.009 |
| M2 | 0.167 | 0.057 | 0.000 | 0.100 | 0.169 | -0.019 | 0.003 | 0.080 | 0.077 | 0.100 | 0.080 | 0.077 | -0.003 | 0.019 | 0.095 | 0.046 |
| LFC | 0.127 | -0.010 | 0.059 | 0.147 | 0.270 | 0.008 | -0.029 | 0.090 | 0.068 | 0.113 | 0.076 | 0.071 | 0.006 | 0.042 | 0.087 | 0.044 |
| ALM | 0.104 | 0.089 | 0.031 | 0.043 | 0.155 | 0.033 | 0.018 | 0.030 | 0.056 | 0.062 | 0.054 | 0.040 | 0.000 | 0.019 | 0.052 | 0.028 |
| M1 | 0.032 | -0.003 | 0.002 | 0.133 | 0.235 | 0.061 | 0.040 | 0.146 | 0.130 | 0.152 | 0.069 | 0.127 | 0.027 | 0.064 | 0.153 | 0.102 |
| A33 | 0.052 | 0.116 | 0.039 | 0.127 | 0.221 | 0.154 | 0.032 | 0.087 | 0.080 | 0.101 | 0.027 | 0.018 | 0.020 | 0.119 | 0.078 | 0.064 |
| A24a | 0.080 | 0.104 | 0.036 | 0.125 | 0.231 | 0.133 | 0.058 | 0.087 | 0.083 | 0.092 | 0.014 | 0.041 | -0.016 | 0.098 | 0.095 | 0.061 |
| A24b | 0.035 | 0.021 | -0.009 | 0.104 | 0.227 | 0.069 | 0.041 | 0.115 | 0.079 | 0.101 | 0.031 | 0.064 | -0.027 | 0.083 | 0.137 | 0.081 |
| STN | 0.094 | 0.114 | 0.054 | 0.075 | 0.209 | 0.136 | 0.074 | -0.015 | -0.015 | 0.033 | -0.007 | 0.005 | -0.026 | 0.088 | 0.087 | 0.052 |
| DLSd | 0.119 | 0.054 | 0.068 | 0.132 | 0.259 | 0.003 | 0.022 | -0.026 | -0.003 | 0.014 | -0.026 | 0.023 | -0.050 | 0.024 | -0.007 | 0.004 |
| DLSv | 0.109 | 0.008 | 0.060 | 0.131 | 0.212 | 0.024 | 0.034 | 0.021 | 0.022 | 0.042 | 0.019 | 0.035 | -0.022 | 0.047 | 0.045 | 0.025 |
| vOFC | 0.074 | 0.189 | 0.024 | 0.067 | 0.098 | 0.142 | 0.044 | 0.049 | 0.026 | 0.038 | 0.035 | 0.071 | 0.056 | 0.082 | 0.029 | 0.069 |
| l OFC | NaN | 0.167 | -0.015 | 0.079 | 0.057 | 0.100 | 0.014 | -0.003 | -0.004 | 0.045 | -0.007 | 0.006 | 0.021 | 0.038 | 0.028 | 0.066 |
| A Ins | 0.167 | NaN | 0.036 | 0.168 | 0.272 | 0.005 | -0.012 | 0.066 | 0.039 | 0.078 | 0.038 | 0.087 | 0.007 | 0.021 | 0.073 | 0.050 |
| NAcS | -0.015 | 0.036 | NaN | 0.016 | 0.053 | 0.030 | 0.013 | -0.017 | 0.011 | 0.010 | 0.022 | 0.017 | 0.016 | 0.000 | 0.015 | 0.002 |
| NacC | 0.079 | 0.168 | 0.016 | NaN | 0.074 | 0.080 | 0.066 | 0.035 | 0.014 | 0.040 | 0.049 | 0.064 | 0.046 | 0.033 | 0.022 | 0.041 |
| VMS | 0.057 | 0.272 | 0.053 | 0.074 | NaN | 0.190 | 0.050 | 0.110 | 0.076 | 0.080 | 0.068 | 0.124 | 0.065 | 0.128 | 0.068 | 0.121 |
| CEA | 0.100 | 0.005 | 0.030 | 0.080 | 0.190 | NaN | 0.020 | 0.019 | 0.016 | 0.024 | 0.058 | 0.064 | 0.020 | 0.051 | 0.024 | 0.032 |
| BLA | 0.014 | -0.012 | 0.013 | 0.066 | 0.050 | 0.020 | NaN | -0.023 | -0.001 | 0.010 | 0.010 | -0.011 | 0.008 | 0.066 | 0.011 | 0.033 |
| V1d | -0.003 | 0.066 | -0.017 | 0.035 | 0.110 | 0.019 | -0.023 | NaN | -0.010 | -0.022 | 0.055 | 0.043 | 0.066 | -0.003 | -0.101 | -0.015 |
| V1v | -0.004 | 0.039 | 0.011 | 0.014 | 0.076 | 0.016 | -0.001 | -0.010 | NaN | -0.032 | 0.100 | 0.063 | 0.119 | 0.027 | -0.039 | 0.066 |
| PPCx | 0.045 | 0.078 | 0.010 | 0.040 | 0.080 | 0.024 | 0.010 | -0.022 | -0.032 | NaN | 0.045 | 0.042 | 0.016 | 0.047 | 0.024 | 0.119 |
| DS | -0.007 | 0.038 | 0.022 | 0.049 | 0.068 | 0.058 | 0.010 | 0.055 | 0.100 | 0.045 | NaN | 0.018 | -0.004 | 0.066 | 0.010 | 0.064 |
| DG | 0.006 | 0.087 | 0.017 | 0.064 | 0.124 | 0.064 | -0.011 | 0.043 | 0.063 | 0.042 | 0.018 | NaN | 0.036 | 0.050 | 0.041 | 0.072 |
| CA1 | 0.021 | 0.007 | 0.016 | 0.046 | 0.065 | 0.020 | 0.008 | 0.066 | 0.119 | 0.016 | -0.004 | 0.036 | NaN | -0.037 | -0.031 | 0.013 |
| CA3 | 0.038 | 0.021 | 0.000 | 0.033 | 0.128 | 0.051 | 0.066 | -0.003 | 0.027 | 0.047 | 0.066 | 0.050 | -0.037 | NaN | 0.069 | 0.126 |
| A30c | 0.028 | 0.073 | 0.015 | 0.022 | 0.068 | 0.024 | 0.011 | -0.101 | -0.039 | 0.024 | 0.010 | 0.041 | -0.031 | 0.069 | NaN | 0.091 |
| A29c | 0.066 | 0.050 | 0.002 | 0.041 | 0.121 | 0.032 | 0.033 | -0.015 | 0.066 | 0.119 | 0.064 | 0.072 | 0.013 | 0.126 | 0.091 | NaN |

SEM Theta wPLI (500-2000ms), [Wait – Go], electrodes 1:16

|  | **A32V** | **A32D** | **DMS** | **MDT** | **CMT** | **M2** | **LFC** | **ALM** | **M1** | **A33** | **A24a** | **A24b** | **STN** | **DLSd** | **DLSv** | **vOFC** |
| --- | --- | --- | --- | --- | --- | --- | --- | --- | --- | --- | --- | --- | --- | --- | --- | --- |
| A32V | NaN | 0.214 | 0.190 | 0.092 | 0.107 | 0.169 | 0.236 | 0.155 | 0.088 | 0.099 | 0.081 | 0.082 | 0.084 | 0.100 | 0.158 | 0.164 |
| A32D | 0.214 | NaN | 0.212 | 0.068 | 0.090 | 0.147 | 0.144 | 0.127 | 0.097 | 0.142 | 0.089 | 0.107 | 0.101 | 0.082 | 0.087 | 0.163 |
| DMS | 0.190 | 0.212 | NaN | 0.188 | 0.187 | 0.175 | 0.218 | 0.183 | 0.167 | 0.193 | 0.230 | 0.143 | 0.197 | 0.173 | 0.165 | 0.119 |
| MDT | 0.092 | 0.068 | 0.188 | NaN | 0.146 | 0.093 | 0.117 | 0.146 | 0.092 | 0.140 | 0.155 | 0.129 | 0.132 | 0.126 | 0.125 | 0.139 |
| CMT | 0.107 | 0.090 | 0.187 | 0.146 | NaN | 0.086 | 0.152 | 0.113 | 0.063 | 0.120 | 0.137 | 0.109 | 0.147 | 0.103 | 0.129 | 0.142 |
| M2 | 0.169 | 0.147 | 0.175 | 0.093 | 0.086 | NaN | 0.139 | 0.065 | 0.094 | 0.163 | 0.131 | 0.074 | 0.111 | 0.085 | 0.101 | 0.154 |
| LFC | 0.236 | 0.144 | 0.218 | 0.117 | 0.152 | 0.139 | NaN | 0.099 | 0.090 | 0.194 | 0.156 | 0.082 | 0.127 | 0.099 | 0.104 | 0.182 |
| ALM | 0.155 | 0.127 | 0.183 | 0.146 | 0.113 | 0.065 | 0.099 | NaN | 0.079 | 0.124 | 0.128 | 0.083 | 0.105 | 0.071 | 0.083 | 0.196 |
| M1 | 0.088 | 0.097 | 0.167 | 0.092 | 0.063 | 0.094 | 0.090 | 0.079 | NaN | 0.098 | 0.095 | 0.066 | 0.070 | 0.070 | 0.077 | 0.131 |
| A33 | 0.099 | 0.142 | 0.193 | 0.140 | 0.120 | 0.163 | 0.194 | 0.124 | 0.098 | NaN | 0.147 | 0.096 | 0.130 | 0.132 | 0.161 | 0.116 |
| A24a | 0.081 | 0.089 | 0.230 | 0.155 | 0.137 | 0.131 | 0.156 | 0.128 | 0.095 | 0.147 | NaN | 0.071 | 0.088 | 0.102 | 0.119 | 0.099 |
| A24b | 0.082 | 0.107 | 0.143 | 0.129 | 0.109 | 0.074 | 0.082 | 0.083 | 0.066 | 0.096 | 0.071 | NaN | 0.075 | 0.097 | 0.094 | 0.118 |
| STN | 0.084 | 0.101 | 0.197 | 0.132 | 0.147 | 0.111 | 0.127 | 0.105 | 0.070 | 0.130 | 0.088 | 0.075 | NaN | 0.099 | 0.145 | 0.110 |
| DLSd | 0.100 | 0.082 | 0.173 | 0.126 | 0.103 | 0.085 | 0.099 | 0.071 | 0.070 | 0.132 | 0.102 | 0.097 | 0.099 | NaN | 0.087 | 0.133 |
| DLSv | 0.158 | 0.087 | 0.165 | 0.125 | 0.129 | 0.101 | 0.104 | 0.083 | 0.077 | 0.161 | 0.119 | 0.094 | 0.145 | 0.087 | NaN | 0.180 |
| vOFC | 0.164 | 0.163 | 0.119 | 0.139 | 0.142 | 0.154 | 0.182 | 0.196 | 0.131 | 0.116 | 0.099 | 0.118 | 0.110 | 0.133 | 0.180 | NaN |
| l OFC | 0.172 | 0.194 | 0.093 | 0.213 | 0.265 | 0.212 | 0.253 | 0.170 | 0.155 | 0.158 | 0.144 | 0.083 | 0.147 | 0.194 | 0.146 | 0.155 |
| A Ins | 0.238 | 0.174 | 0.217 | 0.143 | 0.178 | 0.137 | 0.084 | 0.084 | 0.102 | 0.223 | 0.190 | 0.088 | 0.136 | 0.105 | 0.107 | 0.175 |
| NAcS | 0.126 | 0.131 | 0.073 | 0.106 | 0.111 | 0.148 | 0.120 | 0.104 | 0.072 | 0.141 | 0.090 | 0.079 | 0.098 | 0.118 | 0.144 | 0.088 |
| NacC | 0.183 | 0.157 | 0.081 | 0.151 | 0.127 | 0.089 | 0.128 | 0.106 | 0.134 | 0.132 | 0.105 | 0.122 | 0.116 | 0.127 | 0.146 | 0.145 |
| VMS | 0.225 | 0.242 | 0.112 | 0.213 | 0.235 | 0.206 | 0.223 | 0.216 | 0.197 | 0.219 | 0.207 | 0.165 | 0.215 | 0.187 | 0.190 | 0.186 |
| CEA | 0.188 | 0.086 | 0.170 | 0.118 | 0.133 | 0.073 | 0.097 | 0.090 | 0.101 | 0.159 | 0.125 | 0.090 | 0.165 | 0.084 | 0.069 | 0.200 |
| BLA | 0.138 | 0.130 | 0.126 | 0.115 | 0.128 | 0.067 | 0.106 | 0.105 | 0.102 | 0.146 | 0.096 | 0.092 | 0.169 | 0.096 | 0.127 | 0.113 |
| V1d | 0.061 | 0.110 | 0.107 | 0.092 | 0.068 | 0.118 | 0.109 | 0.088 | 0.122 | 0.087 | 0.108 | 0.092 | 0.069 | 0.089 | 0.073 | 0.086 |
| V1v | 0.068 | 0.122 | 0.100 | 0.192 | 0.121 | 0.125 | 0.101 | 0.120 | 0.139 | 0.103 | 0.106 | 0.113 | 0.116 | 0.101 | 0.076 | 0.082 |
| PPCx | 0.042 | 0.099 | 0.085 | 0.109 | 0.130 | 0.119 | 0.090 | 0.111 | 0.108 | 0.107 | 0.117 | 0.100 | 0.101 | 0.071 | 0.112 | 0.057 |
| DS | 0.060 | 0.065 | 0.114 | 0.135 | 0.125 | 0.083 | 0.105 | 0.076 | 0.093 | 0.107 | 0.107 | 0.075 | 0.086 | 0.105 | 0.085 | 0.072 |
| DG | 0.084 | 0.085 | 0.111 | 0.132 | 0.135 | 0.096 | 0.102 | 0.078 | 0.131 | 0.100 | 0.092 | 0.099 | 0.114 | 0.094 | 0.105 | 0.115 |
| CA1 | 0.076 | 0.072 | 0.124 | 0.108 | 0.110 | 0.066 | 0.053 | 0.071 | 0.082 | 0.087 | 0.079 | 0.120 | 0.093 | 0.084 | 0.081 | 0.074 |
| CA3 | 0.079 | 0.085 | 0.115 | 0.095 | 0.114 | 0.085 | 0.086 | 0.098 | 0.086 | 0.133 | 0.157 | 0.140 | 0.152 | 0.080 | 0.068 | 0.102 |
| A30c | 0.056 | 0.117 | 0.092 | 0.112 | 0.103 | 0.099 | 0.089 | 0.136 | 0.091 | 0.093 | 0.098 | 0.120 | 0.157 | 0.078 | 0.132 | 0.051 |
| A29c | 0.071 | 0.087 | 0.172 | 0.129 | 0.110 | 0.086 | 0.093 | 0.148 | 0.130 | 0.119 | 0.153 | 0.130 | 0.081 | 0.097 | 0.116 | 0.073 |

SEM Theta wPLI (500-2000ms), [Wait – Go], electrodes 17:32

|  | **l OFC** | **A Ins** | **NAcS** | **NacC** | **VMS** | **CEA** | **BLA** | **V1d** | **V1v** | **PPCx** | **DS** | **DG** | **CA1** | **CA3** | **A30c** | **A29c** |
| --- | --- | --- | --- | --- | --- | --- | --- | --- | --- | --- | --- | --- | --- | --- | --- | --- |
| A32V | 0.172 | 0.238 | 0.126 | 0.183 | 0.225 | 0.188 | 0.138 | 0.061 | 0.068 | 0.042 | 0.060 | 0.084 | 0.076 | 0.079 | 0.056 | 0.071 |
| A32D | 0.194 | 0.174 | 0.131 | 0.157 | 0.242 | 0.086 | 0.130 | 0.110 | 0.122 | 0.099 | 0.065 | 0.085 | 0.072 | 0.085 | 0.117 | 0.087 |
| DMS | 0.093 | 0.217 | 0.073 | 0.081 | 0.112 | 0.170 | 0.126 | 0.107 | 0.100 | 0.085 | 0.114 | 0.111 | 0.124 | 0.115 | 0.092 | 0.172 |
| MDT | 0.213 | 0.143 | 0.106 | 0.151 | 0.213 | 0.118 | 0.115 | 0.092 | 0.192 | 0.109 | 0.135 | 0.132 | 0.108 | 0.095 | 0.112 | 0.129 |
| CMT | 0.265 | 0.178 | 0.111 | 0.127 | 0.235 | 0.133 | 0.128 | 0.068 | 0.121 | 0.130 | 0.125 | 0.135 | 0.110 | 0.114 | 0.103 | 0.110 |
| M2 | 0.212 | 0.137 | 0.148 | 0.089 | 0.206 | 0.073 | 0.067 | 0.118 | 0.125 | 0.119 | 0.083 | 0.096 | 0.066 | 0.085 | 0.099 | 0.086 |
| LFC | 0.253 | 0.084 | 0.120 | 0.128 | 0.223 | 0.097 | 0.106 | 0.109 | 0.101 | 0.090 | 0.105 | 0.102 | 0.053 | 0.086 | 0.089 | 0.093 |
| ALM | 0.170 | 0.084 | 0.104 | 0.106 | 0.216 | 0.090 | 0.105 | 0.088 | 0.120 | 0.111 | 0.076 | 0.078 | 0.071 | 0.098 | 0.136 | 0.148 |
| M1 | 0.155 | 0.102 | 0.072 | 0.134 | 0.197 | 0.101 | 0.102 | 0.122 | 0.139 | 0.108 | 0.093 | 0.131 | 0.082 | 0.086 | 0.091 | 0.130 |
| A33 | 0.158 | 0.223 | 0.141 | 0.132 | 0.219 | 0.159 | 0.146 | 0.087 | 0.103 | 0.107 | 0.107 | 0.100 | 0.087 | 0.133 | 0.093 | 0.119 |
| A24a | 0.144 | 0.190 | 0.090 | 0.105 | 0.207 | 0.125 | 0.096 | 0.108 | 0.106 | 0.117 | 0.107 | 0.092 | 0.079 | 0.157 | 0.098 | 0.153 |
| A24b | 0.083 | 0.088 | 0.079 | 0.122 | 0.165 | 0.090 | 0.092 | 0.092 | 0.113 | 0.100 | 0.075 | 0.099 | 0.120 | 0.140 | 0.120 | 0.130 |
| STN | 0.147 | 0.136 | 0.098 | 0.116 | 0.215 | 0.165 | 0.169 | 0.069 | 0.116 | 0.101 | 0.086 | 0.114 | 0.093 | 0.152 | 0.157 | 0.081 |
| DLSd | 0.194 | 0.105 | 0.118 | 0.127 | 0.187 | 0.084 | 0.096 | 0.089 | 0.101 | 0.071 | 0.105 | 0.094 | 0.084 | 0.080 | 0.078 | 0.097 |
| DLSv | 0.146 | 0.107 | 0.144 | 0.146 | 0.190 | 0.069 | 0.127 | 0.073 | 0.076 | 0.112 | 0.085 | 0.105 | 0.081 | 0.068 | 0.132 | 0.116 |
| vOFC | 0.155 | 0.175 | 0.088 | 0.145 | 0.186 | 0.200 | 0.113 | 0.086 | 0.082 | 0.057 | 0.072 | 0.115 | 0.074 | 0.102 | 0.051 | 0.073 |
| l OFC | NaN | 0.244 | 0.159 | 0.143 | 0.181 | 0.150 | 0.096 | 0.081 | 0.081 | 0.088 | 0.095 | 0.089 | 0.085 | 0.106 | 0.089 | 0.172 |
| A Ins | 0.244 | NaN | 0.113 | 0.129 | 0.229 | 0.108 | 0.100 | 0.126 | 0.110 | 0.104 | 0.080 | 0.091 | 0.060 | 0.077 | 0.086 | 0.110 |
| NAcS | 0.159 | 0.113 | NaN | 0.114 | 0.142 | 0.120 | 0.142 | 0.076 | 0.096 | 0.088 | 0.135 | 0.096 | 0.079 | 0.079 | 0.072 | 0.093 |
| NacC | 0.143 | 0.129 | 0.114 | NaN | 0.128 | 0.137 | 0.144 | 0.088 | 0.075 | 0.073 | 0.073 | 0.084 | 0.095 | 0.094 | 0.088 | 0.093 |
| VMS | 0.181 | 0.229 | 0.142 | 0.128 | NaN | 0.203 | 0.135 | 0.094 | 0.091 | 0.085 | 0.131 | 0.125 | 0.101 | 0.115 | 0.087 | 0.151 |
| CEA | 0.150 | 0.108 | 0.120 | 0.137 | 0.203 | NaN | 0.081 | 0.077 | 0.089 | 0.085 | 0.081 | 0.135 | 0.092 | 0.063 | 0.081 | 0.094 |
| BLA | 0.096 | 0.100 | 0.142 | 0.144 | 0.135 | 0.081 | NaN | 0.067 | 0.077 | 0.075 | 0.067 | 0.101 | 0.090 | 0.143 | 0.076 | 0.087 |
| V1d | 0.081 | 0.126 | 0.076 | 0.088 | 0.094 | 0.077 | 0.067 | NaN | 0.121 | 0.111 | 0.112 | 0.092 | 0.150 | 0.081 | 0.127 | 0.103 |
| V1v | 0.081 | 0.110 | 0.096 | 0.075 | 0.091 | 0.089 | 0.077 | 0.121 | NaN | 0.156 | 0.128 | 0.139 | 0.172 | 0.107 | 0.127 | 0.170 |
| PPCx | 0.088 | 0.104 | 0.088 | 0.073 | 0.085 | 0.085 | 0.075 | 0.111 | 0.156 | NaN | 0.090 | 0.092 | 0.155 | 0.145 | 0.116 | 0.107 |
| DS | 0.095 | 0.080 | 0.135 | 0.073 | 0.131 | 0.081 | 0.067 | 0.112 | 0.128 | 0.090 | NaN | 0.100 | 0.167 | 0.145 | 0.122 | 0.157 |
| DG | 0.089 | 0.091 | 0.096 | 0.084 | 0.125 | 0.135 | 0.101 | 0.092 | 0.139 | 0.092 | 0.100 | NaN | 0.156 | 0.131 | 0.119 | 0.160 |
| CA1 | 0.085 | 0.060 | 0.079 | 0.095 | 0.101 | 0.092 | 0.090 | 0.150 | 0.172 | 0.155 | 0.167 | 0.156 | NaN | 0.087 | 0.139 | 0.245 |
| CA3 | 0.106 | 0.077 | 0.079 | 0.094 | 0.115 | 0.063 | 0.143 | 0.081 | 0.107 | 0.145 | 0.145 | 0.131 | 0.087 | NaN | 0.146 | 0.149 |
| A30c | 0.089 | 0.086 | 0.072 | 0.088 | 0.087 | 0.081 | 0.076 | 0.127 | 0.127 | 0.116 | 0.122 | 0.119 | 0.139 | 0.146 | NaN | 0.098 |
| A29c | 0.172 | 0.110 | 0.093 | 0.093 | 0.151 | 0.094 | 0.087 | 0.103 | 0.170 | 0.107 | 0.157 | 0.160 | 0.245 | 0.149 | 0.098 | NaN |

Adj. p-values Theta wPLI (500-2000ms), [Wait – Go], electrodes 1:16

|  | **A32V** | **A32D** | **DMS** | **MDT** | **CMT** | **M2** | **LFC** | **ALM** | **M1** | **A33** | **A24a** | **A24b** | **STN** | **DLSd** | **DLSv** | **vOFC** |
| --- | --- | --- | --- | --- | --- | --- | --- | --- | --- | --- | --- | --- | --- | --- | --- | --- |
| A32V | NaN | 0.153 | 0.096 | 0.020 | 0.003 | 0.218 | 0.232 | 0.141 | 0.070 | 0.041 | 0.039 | 0.081 | 0.066 | 0.090 | 0.137 | 0.098 |
| A32D | 0.153 | NaN | 0.105 | -0.029 | -0.016 | 0.090 | 0.093 | 0.010 | 0.059 | 0.114 | 0.046 | 0.022 | 0.020 | -0.010 | 0.013 | 0.160 |
| DMS | 0.096 | 0.105 | NaN | 0.156 | 0.207 | 0.137 | 0.147 | 0.119 | 0.227 | 0.124 | 0.140 | 0.160 | 0.153 | 0.166 | 0.150 | 0.057 |
| MDT | 0.020 | -0.029 | 0.156 | NaN | -0.025 | -0.012 | 0.039 | -0.001 | 0.007 | 0.059 | 0.071 | 0.020 | 0.016 | 0.003 | 0.027 | 0.060 |
| CMT | 0.003 | -0.016 | 0.207 | -0.025 | NaN | 0.040 | 0.071 | 0.032 | 0.046 | 0.114 | 0.088 | 0.026 | 0.011 | 0.007 | 0.062 | 0.071 |
| M2 | 0.218 | 0.090 | 0.137 | -0.012 | 0.040 | NaN | 0.036 | 0.040 | -0.024 | 0.208 | 0.180 | 0.011 | 0.055 | -0.002 | -0.019 | 0.161 |
| LFC | 0.232 | 0.093 | 0.147 | 0.039 | 0.071 | 0.036 | NaN | 0.094 | -0.001 | 0.142 | 0.117 | 0.050 | 0.101 | 0.050 | 0.030 | 0.202 |
| ALM | 0.141 | 0.010 | 0.119 | -0.001 | 0.032 | 0.040 | 0.094 | NaN | 0.016 | 0.100 | 0.064 | 0.010 | 0.055 | -0.006 | 0.019 | 0.109 |
| M1 | 0.070 | 0.059 | 0.227 | 0.007 | 0.046 | -0.024 | -0.001 | 0.016 | NaN | 0.139 | 0.089 | 0.022 | 0.049 | -0.006 | 0.030 | 0.123 |
| A33 | 0.041 | 0.114 | 0.124 | 0.059 | 0.114 | 0.208 | 0.142 | 0.100 | 0.139 | NaN | -0.001 | 0.067 | 0.086 | 0.108 | 0.117 | 0.076 |
| A24a | 0.039 | 0.046 | 0.140 | 0.071 | 0.088 | 0.180 | 0.117 | 0.064 | 0.089 | -0.001 | NaN | 0.072 | 0.051 | 0.084 | 0.115 | 0.137 |
| A24b | 0.081 | 0.022 | 0.160 | 0.020 | 0.026 | 0.011 | 0.050 | 0.010 | 0.022 | 0.067 | 0.072 | NaN | 0.035 | 0.014 | 0.038 | 0.144 |
| STN | 0.066 | 0.020 | 0.153 | 0.016 | 0.011 | 0.055 | 0.101 | 0.055 | 0.049 | 0.086 | 0.051 | 0.035 | NaN | 0.088 | 0.169 | 0.091 |
| DLSd | 0.090 | -0.010 | 0.166 | 0.003 | 0.007 | -0.002 | 0.050 | -0.006 | -0.006 | 0.108 | 0.084 | 0.014 | 0.088 | NaN | 0.023 | 0.113 |
| DLSv | 0.137 | 0.013 | 0.150 | 0.027 | 0.062 | -0.019 | 0.030 | 0.019 | 0.030 | 0.117 | 0.115 | 0.038 | 0.169 | 0.023 | NaN | 0.133 |
| vOFC | 0.098 | 0.160 | 0.057 | 0.060 | 0.071 | 0.161 | 0.202 | 0.109 | 0.123 | 0.076 | 0.137 | 0.144 | 0.091 | 0.113 | 0.133 | NaN |
| l OFC | 0.124 | 0.222 | 0.005 | 0.101 | 0.151 | 0.167 | 0.127 | 0.104 | 0.032 | 0.052 | 0.080 | 0.035 | 0.094 | 0.119 | 0.109 | 0.074 |
| A Ins | 0.199 | 0.083 | 0.102 | 0.051 | 0.083 | 0.057 | -0.010 | 0.089 | -0.003 | 0.116 | 0.104 | 0.021 | 0.114 | 0.054 | 0.008 | 0.189 |
| NAcS | 0.050 | 0.036 | 0.025 | 0.020 | 0.019 | 0.000 | 0.059 | 0.031 | 0.002 | 0.039 | 0.036 | -0.009 | 0.054 | 0.068 | 0.060 | 0.024 |
| NacC | 0.152 | 0.162 | 0.086 | 0.049 | 0.047 | 0.100 | 0.147 | 0.043 | 0.133 | 0.127 | 0.125 | 0.104 | 0.075 | 0.132 | 0.131 | 0.067 |
| VMS | 0.191 | 0.262 | 0.126 | 0.143 | 0.201 | 0.169 | 0.270 | 0.155 | 0.235 | 0.221 | 0.231 | 0.227 | 0.209 | 0.259 | 0.212 | 0.098 |
| CEA | 0.160 | 0.060 | 0.154 | 0.041 | 0.060 | -0.019 | 0.008 | 0.033 | 0.061 | 0.154 | 0.133 | 0.069 | 0.136 | 0.003 | 0.024 | 0.142 |
| BLA | 0.040 | 0.020 | 0.078 | 0.021 | -0.014 | 0.003 | -0.029 | 0.018 | 0.040 | 0.032 | 0.058 | 0.041 | 0.074 | 0.022 | 0.034 | 0.044 |
| V1d | 0.034 | 0.078 | 0.114 | -0.016 | 0.006 | 0.080 | 0.090 | 0.030 | 0.146 | 0.087 | 0.087 | 0.115 | -0.015 | -0.026 | 0.021 | 0.049 |
| V1v | 0.002 | 0.046 | 0.103 | -0.022 | 0.013 | 0.077 | 0.068 | 0.056 | 0.130 | 0.080 | 0.083 | 0.079 | -0.015 | -0.003 | 0.022 | 0.026 |
| PPCx | 0.002 | 0.053 | 0.123 | 0.024 | 0.059 | 0.100 | 0.113 | 0.062 | 0.152 | 0.101 | 0.092 | 0.101 | 0.033 | 0.014 | 0.042 | 0.038 |
| DS | 0.024 | 0.031 | 0.084 | 0.009 | 0.040 | 0.080 | 0.076 | 0.054 | 0.069 | 0.027 | 0.014 | 0.031 | -0.007 | -0.026 | 0.019 | 0.035 |
| DG | 0.034 | 0.013 | 0.079 | 0.064 | 0.122 | 0.077 | 0.071 | 0.040 | 0.127 | 0.018 | 0.041 | 0.064 | 0.005 | 0.023 | 0.035 | 0.071 |
| CA1 | -0.012 | 0.002 | 0.094 | -0.062 | -0.030 | -0.003 | 0.006 | 0.000 | 0.027 | 0.020 | -0.016 | -0.027 | -0.026 | -0.050 | -0.022 | 0.056 |
| CA3 | 0.019 | 0.014 | 0.151 | 0.029 | 0.072 | 0.019 | 0.042 | 0.019 | 0.064 | 0.119 | 0.098 | 0.083 | 0.088 | 0.024 | 0.047 | 0.082 |
| A30c | -0.001 | 0.043 | 0.094 | 0.039 | 0.055 | 0.095 | 0.087 | 0.052 | 0.153 | 0.078 | 0.095 | 0.137 | 0.087 | -0.007 | 0.045 | 0.029 |
| A29c | 0.017 | 0.025 | 0.148 | -0.011 | 0.009 | 0.046 | 0.044 | 0.028 | 0.102 | 0.064 | 0.061 | 0.081 | 0.052 | 0.004 | 0.025 | 0.069 |

Adj. p-values Theta wPLI (500-2000ms), [Wait – Go], electrodes 17:32

|  | **l OFC** | **A Ins** | **NAcS** | **NacC** | **VMS** | **CEA** | **BLA** | **V1d** | **V1v** | **PPCx** | **DS** | **DG** | **CA1** | **CA3** | **A30c** | **A29c** |
| --- | --- | --- | --- | --- | --- | --- | --- | --- | --- | --- | --- | --- | --- | --- | --- | --- |
| A32V | 0.124 | 0.199 | 0.050 | 0.152 | 0.191 | 0.160 | 0.040 | 0.034 | 0.002 | 0.002 | 0.024 | 0.034 | -0.012 | 0.019 | -0.001 | 0.017 |
| A32D | 0.222 | 0.083 | 0.036 | 0.162 | 0.262 | 0.060 | 0.020 | 0.078 | 0.046 | 0.053 | 0.031 | 0.013 | 0.002 | 0.014 | 0.043 | 0.025 |
| DMS | 0.005 | 0.102 | 0.025 | 0.086 | 0.126 | 0.154 | 0.078 | 0.114 | 0.103 | 0.123 | 0.084 | 0.079 | 0.094 | 0.151 | 0.094 | 0.148 |
| MDT | 0.101 | 0.051 | 0.020 | 0.049 | 0.143 | 0.041 | 0.021 | -0.016 | -0.022 | 0.024 | 0.009 | 0.064 | -0.062 | 0.029 | 0.039 | -0.011 |
| CMT | 0.151 | 0.083 | 0.019 | 0.047 | 0.201 | 0.060 | -0.014 | 0.006 | 0.013 | 0.059 | 0.040 | 0.122 | -0.030 | 0.072 | 0.055 | 0.009 |
| M2 | 0.167 | 0.057 | 0.000 | 0.100 | 0.169 | -0.019 | 0.003 | 0.080 | 0.077 | 0.100 | 0.080 | 0.077 | -0.003 | 0.019 | 0.095 | 0.046 |
| LFC | 0.127 | -0.010 | 0.059 | 0.147 | 0.270 | 0.008 | -0.029 | 0.090 | 0.068 | 0.113 | 0.076 | 0.071 | 0.006 | 0.042 | 0.087 | 0.044 |
| ALM | 0.104 | 0.089 | 0.031 | 0.043 | 0.155 | 0.033 | 0.018 | 0.030 | 0.056 | 0.062 | 0.054 | 0.040 | 0.000 | 0.019 | 0.052 | 0.028 |
| M1 | 0.032 | -0.003 | 0.002 | 0.133 | 0.235 | 0.061 | 0.040 | 0.146 | 0.130 | 0.152 | 0.069 | 0.127 | 0.027 | 0.064 | 0.153 | 0.102 |
| A33 | 0.052 | 0.116 | 0.039 | 0.127 | 0.221 | 0.154 | 0.032 | 0.087 | 0.080 | 0.101 | 0.027 | 0.018 | 0.020 | 0.119 | 0.078 | 0.064 |
| A24a | 0.080 | 0.104 | 0.036 | 0.125 | 0.231 | 0.133 | 0.058 | 0.087 | 0.083 | 0.092 | 0.014 | 0.041 | -0.016 | 0.098 | 0.095 | 0.061 |
| A24b | 0.035 | 0.021 | -0.009 | 0.104 | 0.227 | 0.069 | 0.041 | 0.115 | 0.079 | 0.101 | 0.031 | 0.064 | -0.027 | 0.083 | 0.137 | 0.081 |
| STN | 0.094 | 0.114 | 0.054 | 0.075 | 0.209 | 0.136 | 0.074 | -0.015 | -0.015 | 0.033 | -0.007 | 0.005 | -0.026 | 0.088 | 0.087 | 0.052 |
| DLSd | 0.119 | 0.054 | 0.068 | 0.132 | 0.259 | 0.003 | 0.022 | -0.026 | -0.003 | 0.014 | -0.026 | 0.023 | -0.050 | 0.024 | -0.007 | 0.004 |
| DLSv | 0.109 | 0.008 | 0.060 | 0.131 | 0.212 | 0.024 | 0.034 | 0.021 | 0.022 | 0.042 | 0.019 | 0.035 | -0.022 | 0.047 | 0.045 | 0.025 |
| vOFC | 0.074 | 0.189 | 0.024 | 0.067 | 0.098 | 0.142 | 0.044 | 0.049 | 0.026 | 0.038 | 0.035 | 0.071 | 0.056 | 0.082 | 0.029 | 0.069 |
| l OFC | NaN | 0.167 | -0.015 | 0.079 | 0.057 | 0.100 | 0.014 | -0.003 | -0.004 | 0.045 | -0.007 | 0.006 | 0.021 | 0.038 | 0.028 | 0.066 |
| A Ins | 0.167 | NaN | 0.036 | 0.168 | 0.272 | 0.005 | -0.012 | 0.066 | 0.039 | 0.078 | 0.038 | 0.087 | 0.007 | 0.021 | 0.073 | 0.050 |
| NAcS | -0.015 | 0.036 | NaN | 0.016 | 0.053 | 0.030 | 0.013 | -0.017 | 0.011 | 0.010 | 0.022 | 0.017 | 0.016 | 0.000 | 0.015 | 0.002 |
| NacC | 0.079 | 0.168 | 0.016 | NaN | 0.074 | 0.080 | 0.066 | 0.035 | 0.014 | 0.040 | 0.049 | 0.064 | 0.046 | 0.033 | 0.022 | 0.041 |
| VMS | 0.057 | 0.272 | 0.053 | 0.074 | NaN | 0.190 | 0.050 | 0.110 | 0.076 | 0.080 | 0.068 | 0.124 | 0.065 | 0.128 | 0.068 | 0.121 |
| CEA | 0.100 | 0.005 | 0.030 | 0.080 | 0.190 | NaN | 0.020 | 0.019 | 0.016 | 0.024 | 0.058 | 0.064 | 0.020 | 0.051 | 0.024 | 0.032 |
| BLA | 0.014 | -0.012 | 0.013 | 0.066 | 0.050 | 0.020 | NaN | -0.023 | -0.001 | 0.010 | 0.010 | -0.011 | 0.008 | 0.066 | 0.011 | 0.033 |
| V1d | -0.003 | 0.066 | -0.017 | 0.035 | 0.110 | 0.019 | -0.023 | NaN | -0.010 | -0.022 | 0.055 | 0.043 | 0.066 | -0.003 | -0.101 | -0.015 |
| V1v | -0.004 | 0.039 | 0.011 | 0.014 | 0.076 | 0.016 | -0.001 | -0.010 | NaN | -0.032 | 0.100 | 0.063 | 0.119 | 0.027 | -0.039 | 0.066 |
| PPCx | 0.045 | 0.078 | 0.010 | 0.040 | 0.080 | 0.024 | 0.010 | -0.022 | -0.032 | NaN | 0.045 | 0.042 | 0.016 | 0.047 | 0.024 | 0.119 |
| DS | -0.007 | 0.038 | 0.022 | 0.049 | 0.068 | 0.058 | 0.010 | 0.055 | 0.100 | 0.045 | NaN | 0.018 | -0.004 | 0.066 | 0.010 | 0.064 |
| DG | 0.006 | 0.087 | 0.017 | 0.064 | 0.124 | 0.064 | -0.011 | 0.043 | 0.063 | 0.042 | 0.018 | NaN | 0.036 | 0.050 | 0.041 | 0.072 |
| CA1 | 0.021 | 0.007 | 0.016 | 0.046 | 0.065 | 0.020 | 0.008 | 0.066 | 0.119 | 0.016 | -0.004 | 0.036 | NaN | -0.037 | -0.031 | 0.013 |
| CA3 | 0.038 | 0.021 | 0.000 | 0.033 | 0.128 | 0.051 | 0.066 | -0.003 | 0.027 | 0.047 | 0.066 | 0.050 | -0.037 | NaN | 0.069 | 0.126 |
| A30c | 0.028 | 0.073 | 0.015 | 0.022 | 0.068 | 0.024 | 0.011 | -0.101 | -0.039 | 0.024 | 0.010 | 0.041 | -0.031 | 0.069 | NaN | 0.091 |
| A29c | 0.066 | 0.050 | 0.002 | 0.041 | 0.121 | 0.032 | 0.033 | -0.015 | 0.066 | 0.119 | 0.064 | 0.072 | 0.013 | 0.126 | 0.091 | NaN |

**Figure 7_2:** Data table for mean, SEM and p-values for pair-wise wPLI for the [wait-go] contrast. Statistical test used was a one-sample t-test, mean Data averaged at theta-frequencies between 500-2000ms post-stimulus. P-values adjusted for multiple comparisons using FDR-correction.
